# Enhancing the solubility of SARS-CoV-2 inhibitors to increase future prospects for clinical development

**DOI:** 10.1128/jvi.02159-24

**Published:** 2025-02-04

**Authors:** Ariel J. Kuhn, Victor K. Outlaw, Tara C. Marcink, Zhen Yu, Megan C. Mears, Maria N. Cajimat, Dale F. Kreitler, Payton R. Cleven, Jee Ching Mook, Dennis A. Bente, Matteo Porotto, Samuel H. Gellman, Anne Moscona

**Affiliations:** 1 Department of Chemistry, University of Wisconsin 201643 , Madison, Wisconsin, USA; 2 Center for Host–Pathogen Interaction, Columbia University Medical Center 21611 , New York, New York, USA; 3 Department of Pediatrics, Columbia University Medical Center 21611 , New York, New York, USA; 4 Galveston National Laboratory, University of Texas Medical Branch 12338 , Galveston, Texas, USA; 5 Center for BioMolecular Structure, NSLS-II, Brookhaven National Laboratory 8099 , Upton, New York, USA; 6 Department of Experimental Pathology, University of Texas Medical Branch 12338 , Galveston, Texas, USA; 7 Department of Experimental Medicine, University of Campania “Luigi Vanvitelli” 18994 , Caserta, Italy; 8 Department of Microbiology & Immunology, Columbia University Medical Center 21611 , New York, New York, USA; 9 Department of Physiology & Cellular Biophysics, Columbia University Medical Center 21611 , New York, New York, USA; St. Jude Children's Research Hospital, Memphis, Tennessee, USA

**Keywords:** SARS-CoV-2, lipopeptide, solubility, inhibitors, viral fusion, heptad repeats, crystal structure, peptide design, antiviral

## Abstract

SARS-CoV-2 poses an ongoing threat to human health as variants continue to emerge. Several effective vaccines are available, but a diminishing number of Americans receive the updated vaccines (only 22% received the 2023 update). Public hesitancy towards vaccines and common occurrence of “breakthrough” infections (i.e.*,* infections of vaccinated individuals) highlight the need for alternative methods to reduce viral transmission. SARS-CoV-2 enters cells by fusing its envelope with the target cell membrane in a process mediated by the viral spike protein, S. The S protein operates via a Class I fusion mechanism in which fusion between the viral envelope and host cell membrane is mediated by structural rearrangements of the S trimer. We previously reported lipopeptides derived from the C-terminal heptad repeat (HRC) domain of SARS-CoV-2 S that potently inhibit fusion by SARS-CoV-2, both *in vitro* and *in vivo*. These lipopeptides bear an attached cholesterol unit to anchor them in the membrane. Here, to improve prospects for experimental development and future clinical utility, we employed structure-guided design to incorporate charged residues at specific sites in the peptide to enhance aqueous solubility. This effort resulted in two new, potent lipopeptide inhibitors.

**IMPORTANCE:**

Despite the existence of vaccines for SARS-CoV-2, the constant evolution of new variants and the occurrence of breakthrough infections highlight the need for new and effective antiviral approaches. We have shown that lipopeptides designed to bind a conserved region on the SARS-CoV-2 spike protein can effectively block viral entry into cells and thereby block infection. To support the feasibility of using this approach in humans, we re-designed these lipopeptides to be more soluble, using information about the structure of the spike protein interacting with the peptides to modify the peptide chain. The new peptides are effective against both SARS-CoV-2 and MERS. The lipopeptides described here could serve as treatment for people who are unvaccinated or who experience breakthrough infections, and the approach to increasing solubility can be applied in a broad spectrum approach to treating infections with emerging viruses.

## INTRODUCTION

Severe acute respiratory syndrome coronavirus type 2 (SARS-CoV-2), the causative agent of the 2019 coronavirus disease (COVID-19), now appears to be established in the human population. Rapid emergence of new strains and declining interest in vaccination and other preventative measures make this pathogen an ongoing threat, especially for the elderly and those with impaired immune systems. A prophylactic antiviral strategy that prevents viral transmission (1) and maintains efficacy as SARS-CoV-2 evolves would represent a significant contribution to public health.

Infection by SARS-CoV-2 is initiated by fusion of the viral and host cell membranes, a process mediated by the SARS-CoV-2 spike (S) glycoprotein (2, 3). S is a homotrimer, with each monomer consisting of S1 and S2 subunits. A receptor-binding domain (RBD) within the S1 subunit promotes cell surface attachment and activation. Receptor-mediated activation triggers a series of structural transitions that leads to formation of a stable six-helix bundle (6HB) assembly between N- and C-terminal heptad repeat (HRN and HRC) domains within the S2 subunit, ultimately driving membrane fusion and infection.

**Fig 1:**
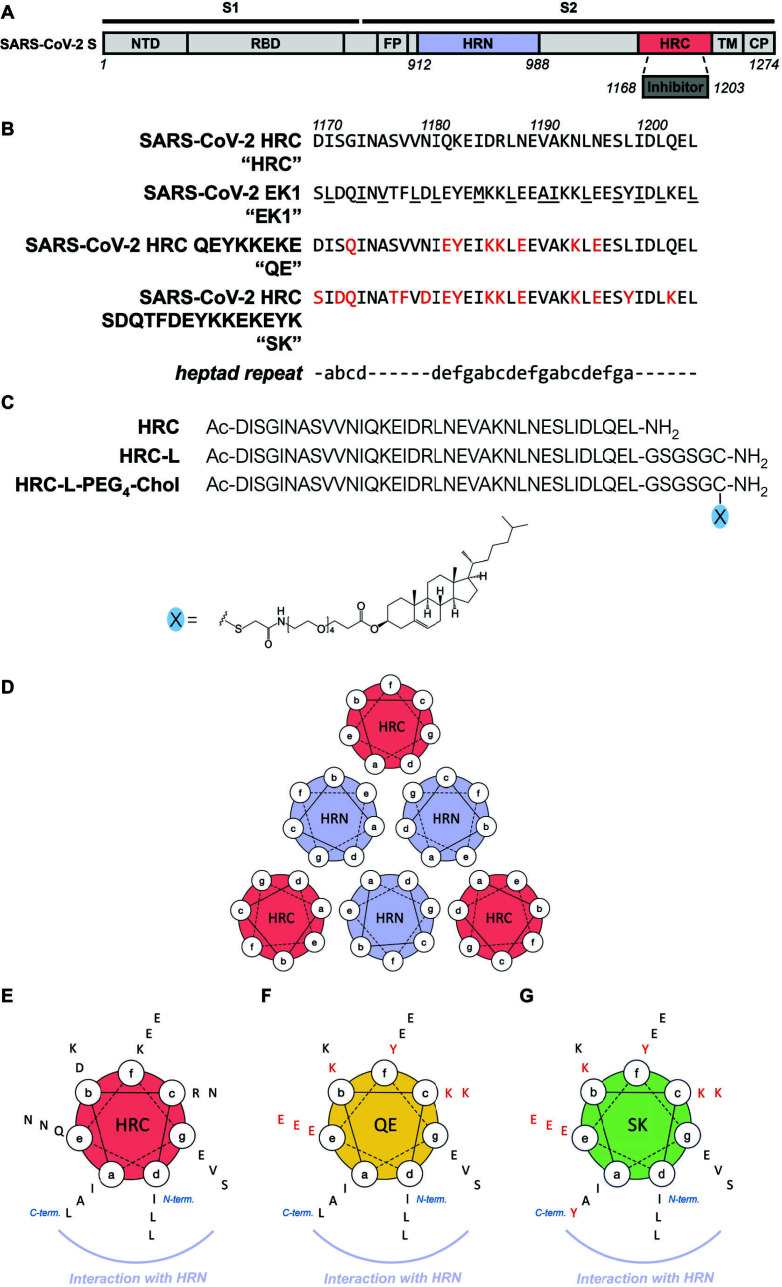
(**A**) Simplified schematic diagram of SARS-CoV-2 S. The N-terminal domain (NTD), receptor-binding domain (RBD), fusion peptide (FP), N-terminal heptad repeat (HRN, periwinkle), C-terminal heptad repeat (HRC, salmon), transmembrane (TM), and cytoplasmic tail (CP) domains are depicted. (**B**) Sequences of the native HRC, EK1, and designed variants, QE and SK. Residue replacements in SK and QE relative to the native HRC are shown in red. (**C**) Definition of peptides referred to in text and the structure of cholesterol-conjugated HRC. (**D**) Analysis of the six-helix bundle assembly formed by HRN (periwinkle) and HRC (salmon). (**E-G**) Analysis of side chain orientation within the α-helical segments of the HRC (E, salmon), QE (F, yellow), and SK (G, green) heptad repeat sequences.

We have previously reported lipopeptides that potently inhibit fusion mediated by SARS-CoV-2 S and block infection by SARS-CoV-2 S in cell monolayers (*in vitro*), human airway tissues (*ex vivo*), and animal models (*in vivo*) (1, 4). These molecules contain a peptide segment corresponding to the HRC domain of SARS-CoV-2 S. The lipopeptides are believed to engage the trimeric HRN domain assembly of SARS-CoV-2 S, thereby disrupting the structural rearrangements of S that drive membrane fusion. Analogous HRC-based lipopeptides prevent infection by several other viruses (HIV, measles, Nipah, parainfluenza, influenza) and can be administered via the airway (1, 4–27). Treatment is effective for some of these viruses even several days after exposure (8). These lipopeptides bear an appended cholesterol moiety that is intended to anchor them in the host cell membrane, thus concentrating the lipopeptides at the site of action and enhancing antiviral efficacy (7–10, 18–20). These precedents suggest that HRC-based lipopeptides could be effective prophylactic agents for protection from COVID. The first lipopeptide inhibitor we described for SARS-CoV-2, designated **HRC-L-PEG**_**4**_**-Chol** here (Fig. 1), was based on a 36-residue segment of the native SARS-CoV-2 S HRC domain (Wuhan strain) (4). This inhibitor was efficacious in cell-based assays (4), and pegylated and/or dimerized derivatives of this lipopeptide were highly effective at preventing SARS-CoV2 transmission *in vivo* (1). The 36-mer peptide and lipopeptide derivatives exhibited poor aqueous solubility and a strong propensity for gelation that hindered synthesis, purification, characterization, and evaluation.

These studies suggested that prospects for clinical development of lipopeptides directed against SARS-CoV-2 infection would be improved if we could enhance the solubility and decrease the gelation propensity of the precursor HRC peptide. Solubility is a critical parameter in drug development and is especially important for intranasal delivery. Low nasal cavity volume allows for a maximum volume of only <500 µL for intranasal delivery in humans; thus, efforts to enhance lipopeptide solubility in early-stage studies should enhance long-term prospects for clinical development (28–34). Here, we describe a rational approach to increasing aqueous solubility of the HRC-derived peptide that resulted in new lipopeptide inhibitors of SARS-CoV-2 infection.

Our design strategy was based on combining features of the native SARS-CoV-2 HRC, which is very potent but has limited solubility, and a peptide derived from the human coronavirus OC43 HRC domain designated **EK1** (Fig. 1B), which was reported by Xia et al. (35–37). **EK1** has lower antiviral potency toward SARS-CoV-2 relative to the native SARS-CoV-2 HRC (4) but displays high solubility (35–37). Xia et al. used the structure of the MERS HRC–HRN six-helix bundle in the **EK1** design process (37), since coronavirus HRC–HRN assemblies are generally similar to one another (38–41), and the OC43 six-helix bundle structure has not been determined. Residue changes that generated **EK1** from the OC43 HRC sequence were located at sites expected to project side chains from the surface of the six-helix bundle into the surrounding solvent; native side chains expected to form close contacts with the HRN trimer were preserved. Most of these changes were located in the central portion of the OC43 HRC, which is expected to adopt an α-helical conformation. For all coronaviruses, the N- and C-terminal portions of each HRC component adopt extended conformations in the six-helix bundle assembly (36, 38–41). The **EK1** design featured non-native lysine and glutamic acid residues in positions that would support formation of solvent-facing salt bridges in the helical conformation. These charged side chains were expected to enhance peptide solubility relative to the native OC43 HRC sequence (37).

**EK1** was reported to inhibit SARS-CoV-2 infection in cell-based assays with a potency comparable to that of the native SARS-CoV-2 HRC peptide (35). Subsequently, it was reported that a cholesterol-bearing derivative of **EK1**, designated EK1C4, was substantially more potent against SARS-CoV-2 infection than **EK1** itself (36), which is consistent with our prior findings that lipopeptides display enhanced antiviral potency relative to unmodified HRC peptides (4, 10). However, we found that an **EK1**-derived lipopeptide was 10- to 100-fold less potent than the native HRC-derived lipopeptide for inhibition of SARS-CoV-2 infection (4).

We reasoned that the poor performance of the **EK1**-derived lipopeptide as an inhibitor of SARS-CoV-2 infection results from the multiple differences between **EK1** and the native SARS-CoV-2 HRC at sites that form intimate contacts with the HRN trimer, which lies at the core of the six-helix bundle assembly. Crystallographic data indicate that 14 of the SARS-CoV-2 HRC residues contribute side chains to the HRC–HRN interface in the native six-helix bundle (36); seven of these interfacial residues differ between the SARS-CoV-2 HRC domain and **EK1**. We therefore hypothesized that the antiviral potency of the native SARS-CoV-2 HRC peptide could be maintained while solubility was improved by combining the solubility-promoting residues of **EK1** with the native SARS-CoV-2 interfacial residues.

## RESULTS

### Peptide design

We aimed to improve upon our previous SARS-CoV-2 peptide design (1, 4) by identifying analogs of the 36-residue SARS-CoV-2 C-terminal heptad repeat region (Fig. 1A and B) that retained the ability to form a six-helix bundle assembly with the SARS-CoV-2 HRN, but displayed higher aqueous solubility and a lower propensity for gel formation relative to native HRC peptide, which is designated as **HRC**. Our design strategy led to two 36-mer sequences, one designated **QE** and the other **SK**. Relative to the native SARS-CoV-2 peptide, **HRC**, **QE** contains eight-residue substitutions derived from **EK1** (Fig. 1B and F). **SK** contains an additional seven-residue substitutions derived from **EK1** (Fig. 1B and F). In both cases, all substitutions are expected to occur at solvent-facing positions of the HRC-derived peptide in a six-helix bundle assembly with the SARS-CoV-2 HRN. In both **QE** and **SK**, all of the native SARS-CoV-2 residues that contact the HRN in the six-helix bundle are preserved.

We used the grand average of hydropathy (GRAVY) score to predict the relative aqueous solubilities of the peptides **QE**, **SK** and **HRC**. GRAVY scores are calculated by summing the individual hydropathy scores for each residue, then dividing by the total number of residues in the peptide or protein sequence (42). The GRAVY scores, −0.197 for **QE**, −0.386 for **SK**, and −0.178 for **HRC**, suggested that our designs would display the desired improvement in solubility.

The ability of an HRC peptide to form a stable six-helix bundle (Fig. 1D) often correlates with the ability of a lipopeptide derivative to inhibit viral infection (8, 10). We have previously shown that cholesterol attachment enhances the antiviral efficacy of peptide-derived fusion inhibitors relative to peptides lacking the lipid appendage (7, 8, 18, 20, 23). Attachment of the cholesterol moiety was achieved by extending the C-terminus with the segment Gly-Ser-Gly-Ser-Gly-Cys (10), and the cysteine side chain was used to attach a tetra-ethylene glycol–cholesterol unit (PEG_4_–Chol; Fig. 1C). We envisioned that the newly designed peptide sequences could be modified in this way to maximize antiviral activity.

### Aqueous solubility of modified peptides

**Fig 2:**
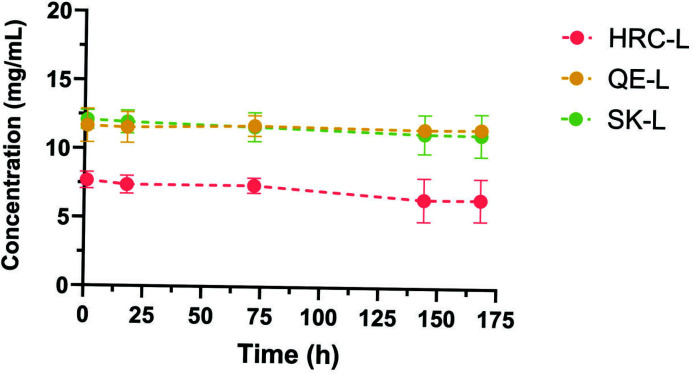
Solubility of HRC-L, QE-L, and SK-L peptides in 50 mM phosphate buffer (pH 7.4) as measured by UV absorbance at 280 (QE-L and SK-L) and 205 nm (HRC-L).

Our published SARS-CoV-2 **HRC** peptide has proved exceptionally difficult to produce and work with, with technical difficulties ranging from precipitation to gel formation (Fig. S1). For experimental comparison of aqueous solubility, we used the peptides **HRC-L**, **QE-L**, and **SK-L**, derivatives of **HRC**, **QE**, and **SK**, respectively, that contain the C-terminal Gly-Ser-Gly-Ser-Gly-Cys extension (Fig. 1C). This “linker” provides the attachment site for the cholesterol unit, as described above. In the solubility measurements, UV absorbance was used to determine peptide concentration (Fig. 2). **QE-L** contains a single residue with an aromatic side chain (Tyr), and **SK-L** contains two such residues (both Tyr). For these peptides, concentration was calculated based on absorbance at 280 nm and a well-established extinction coefficient (43). **HRC-L** does not contain any aromatic side chains, and in this case, concentration was calculated based on absorbance at 205 nm following reported protocols (44). **QE-L** and **SK-L** were ~two-fold more soluble than **HRC-L**; this difference was maintained over 7 days. When solubility was tested for analogous peptides lacking the GSGSGC linker, i.e., for **QE**, **SK,** and **HRC** (Fig. 1C), solubility was similarly reduced in each case (Fig. S2). In addition, **QE, SK**, and their derivatives were substantially less prone to gelation relative to **HRC** and its derivatives (Fig. S1).

### QE and SK form stable assemblies with the native SARS-CoV-2 HRN

We used circular dichroism (CD) data to ask whether the SARS-CoV-2 HRC-derived peptides co-assemble with SARS-CoV-2 HRN-derived peptides in solution to form six-helix bundles similar to those found in the post-fusion state of SARS-CoV-2 S. We have shown that the stability of the HRN–HRC assembly can correlate with antiviral efficacy of HRC-derived peptides from other viruses that employ a Class I fusion mechanism to enter target cells (8, 14, 23, 45). Our CD studies involved 1:1 pairings of **HRC**, **QE**, or **SK** with a 55-residue peptide derived from the native SARS-CoV-2 HRN. Although we use the term “six-helix bundle” to describe the resulting assemblies, as is standard in this field, we acknowledge that for coronaviruses the HRC components are only partially helical in the assembled state (36, 38–41).

**Fig 3:**
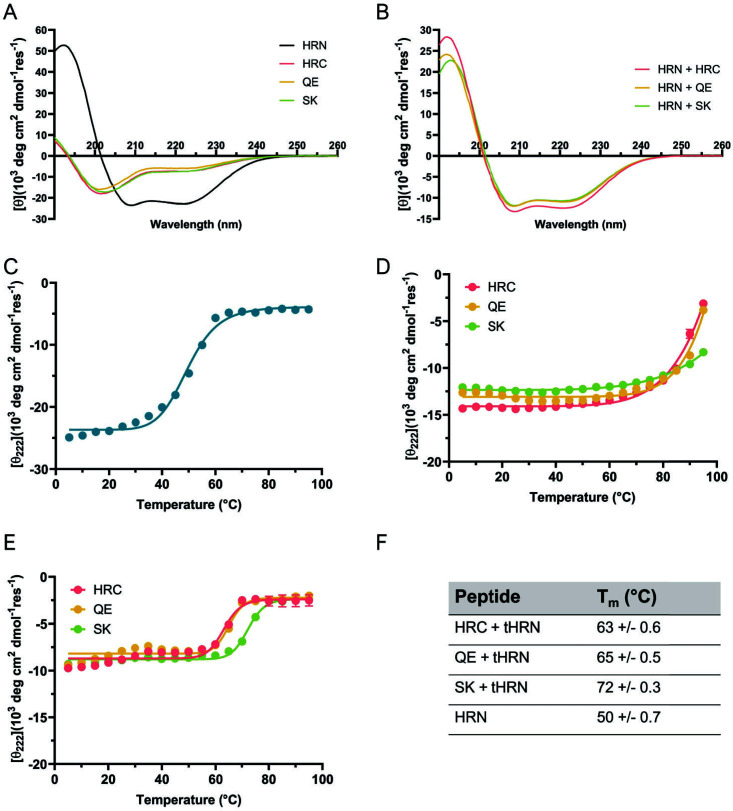
Circular dichroism studies of SARS-CoV-2 HRN + HRC hybrid co-assemblies. Full-spectrum scan at 25°C of (**A**) individual peptides and (**B**) binary peptide mixtures (HRN peptide + HRC, QE, or SK). (**C**) Variable-temperature data for the HRN peptide alone. (**D-E**) Variable–temperature data for binary mixtures of the HRN peptide (**D**) or a truncated derivative (tHRN) (**E**) with HRC, QE, or SK; 50 µM total peptide concentration in 10 mM phosphate buffer, pH 7.4. (**F**) T_m_ values based on variable–temperature data shown in C–E.

CD data in Fig. 3 are presented in terms of mean residue ellipticity, which means that the data are normalized for the number of residues. This approach enables comparisons among peptides with different numbers of residues (e.g., HRC vs HRN). Figure 3A (and Fig. S11) shows data for individual peptides. The HRN peptide displays a strong α-helix signature, with prominent minima at 208 and 222 nm. These data suggest that the HRN peptide at 50 µM spontaneously forms a helical assembly, which is presumed to be the three-helix assembly that lies at the core of the HRC + HRN six-helix bundle. In contrast, the CD data for **HRC, QE**, and **SK** indicate that each of these peptides is largely unfolded at 50 µM in the absence of the HRN peptide.

CD data for a 1:1 mixture of **HRC**, **QE**, or **SK** with the HRN peptide (Fig. 3B; Fig. S6 and S7; 25 μM each peptide) display an α-helix signature in each case, which we interpret to indicate that a six-helix bundle assembly forms in each case. This interpretation is consistent with the fact that **HRC** co-crystallizes with the HRN peptide to form a six-helix bundle (36); we report below that a very similar six-helix bundle is observed in the co-crystal structure involving **QE** and the native HRN peptide. For each of the 1:1 mixtures, the minima at 208 and 222 nm are less intense than for the HRN peptide alone. This observation presumably reflects that fact that many of the HRC residues occur in extended rather than helical conformations in the six-helix bundle assembly (36, 37, 39–41).

Variable–temperature CD (VT-CD) measurements were used to assess the stability of the helix-bundle assemblies; in these measurements, the CD signal at 220 nm was monitored (Fig. 3C through E). VT-CD data for the HRN peptide alone (Fig. 3C) display a sigmoidal transition, which indicates that the helical state present at lower temperatures is cooperatively disrupted upon heating. The midpoint of such transitions is commonly designated the ”melting temperature” (T_m_). In this case, T_m_ = 50°C.

For each 1:1 combination, involving the HRN peptide and **HRC**, **QE**, or **SK**, the helical assembly appears to be stable over much of the accessible temperature range, with disruption detectable only above ~70°C (Fig. 3D). The fully disrupted state does not appear to be reached for any of these pairs at the highest temperature (95°C) because there is no plateau in the signal. These data suggest that all three forms of the six-helix bundle are quite stable. Fully disrupted states have been achieved in related VT-CD studies shown in the SI or reported by others, but in these cases, the HRN and HRC sequences were truncated or otherwise modified relative to the peptides we describe here (Fig. S8 to S10; 36, 46–48). We tested our interpretation of the data in Fig. 3D by examining a second set of 1:1 mixtures containing **HRC**, **QE**, or **SK** (Fig. 3E). In these cases, the 55-residue native HRN peptide was replaced with a truncated variant (tHRN; 44 residues) from which the N-terminal residue and the 10 C-terminal residues of the HRN peptide had been removed (Fig. S11) (36). Based on analysis of the HRC + HRN crystal structure, we predicted that this truncation would destabilize the six-helix bundle. Indeed, all three mixtures with tHRN displayed sigmoidal transitions (Fig. 3E; Fig. S7), implying complete disruption of helix-bundle assemblies over the accessible temperature range. These data suggested that the assemblies formed by **HRC** or **QE** with tHRN were similar in stability, and that the assembly formed by **SK** was slightly more stable. This VT-CD comparison was extended to **EK1**; this 1:1 mixture with tHRN displayed a thermal transition at a substantially lower temperature range relative to the three shown in Fig. 3E, as indicated in Fig. S3.

### Structural mimicry of the native SARS-CoV-2 HRC

**Fig 4:**
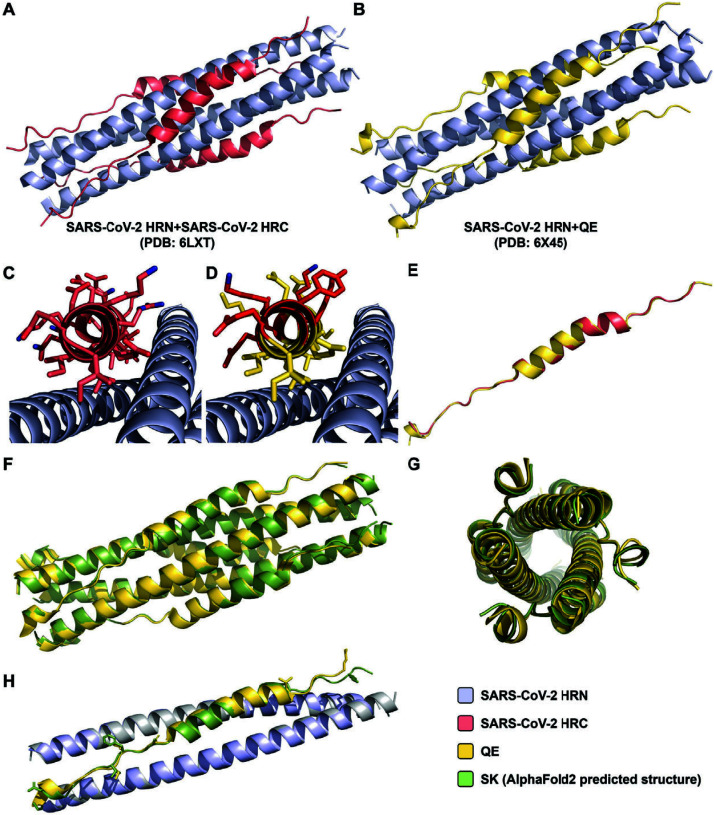
X-ray crystal structure of QE bound to the HRN domain of SARS-CoV-2 S. (**A**) 6HB co-assembly formed between SARS-CoV-2 HRN (periwinkle) and HRC (salmon) (PDB 6LXT). (**B**) 6HB co-assembly formed between SARS-CoV-2 HRN (periwinkle) and QE (yellow) (PDB 6X45). (**C**) Alternate view of PDB 6LXT. (**D**) Alternate view of PDB 6X45 with changed residues shown in bright red. (**E**) Overlay of SARS-CoV-2 HRC (salmon) and QE (yellow). (**F-G**) Overlays of 6HB co-assemblies formed between SARS-CoV-2 HRN with either QE (yellow) or SK (green; AlphaFold2). (**H**) Overlays of 6HB co-assemblies formed between SARS-CoV-2 HRN with either QE (yellow) or SK (green; AlphaFold2) with changed sidechains illustrated. Legend shown in bottom right.

**QE** and the native HRN peptide were co-crystallized. The resulting structure (PDB 6X45; 2.20 Å resolution; Fig. 4A through D) revealed a six-helix bundle nearly identical to the assembly formed by **HRC** and the native HRN peptide (PDB 6LXT; Fig. 4A) (36), with a root mean square deviation (RMSD) of 0.68 Å for the C_α_ atoms. Three copies of the HRN peptide form a parallel trimer around which three copies of **QE** are arrayed (Fig. 4B). The central α-helical portion of each **QE** peptide, spanning residues I1181 to L1199 (S protein numbering), displays a bend similar to that in the native assembly. The residues altered in **QE** relative to **HRC** (highlighted in red in Fig. 4D) are exposed to the solvent, as expected. Non-helical segments are observed at both ends of **QE**, spanning residues N1159–N1180 and I1200–1205L. An overlay of **HRC** and **QE** (Fig. 4E) highlights the conformational similarity of these two peptides. In our new crystal structure, all three copies of **QE** in the helix-bundle assembly are crystallographically non-equivalent, which matches the non-equivalence of the three **HRC** molecules in the previously reported structure. **QE** vs **HRC** comparisons involving different versions of these molecules gave RMSD values of 0.5 to 1.0 Å for the C_α_ atoms. The relatively high R_free_ value of 0.278 is consistent with other coil–coil crystal structures (14, 23, 36).

Extensive efforts to co-crystallize **SK** and the native HRN peptide were unsuccessful. We therefore turned to AlphaFold 2 (ColabFOLD V1.5.5) (49) to predict the structure of this assembly (Fig. 4F through H). The predicted structure was nearly indistinguishable from the six-helix bundle formed by **QE** and the HRN peptide (Fig. 4D) with an RMSD of 0.36 Å for the C_α_ atoms.

### Inhibition of fusion mediated by SARS-CoV-2 S

We compared the efficacy of the lipopeptide derivatives of **HRC**, **QE**, and **SK** for inhibiting fusion mediated by SARS-CoV-2 S using a cell–cell fusion assay. The lipopeptides were generated from derivatives of **HRC**, **QE**, and **SK** bearing a Gly-Ser-Gly-Ser-Gly-Cys linker segment at the C-terminus, and the cysteine side chain was used as an attachment point for the tetra-ethylene glycol–cholesterol (PEG_4_–Chol) appendage (Fig. 1C). The resulting lipopeptides are designated **HRC-L-PEG_4_-Chol, QE-L-PEG_4_-Chol**, and **SK-L-PEG_4_-Chol**. In the assay, cells expressing human angiotensin-converting enzyme 2 (hACE2), the receptor for SARS-CoV-2 S, and the N-terminal portion of β-galactosidase (β-gal) were mixed with cells expressing either the D614G or BA.5 SARS-CoV-2 S protein and the C-terminal portion of β-gal. When fusion mediated by S occurs, the two portions of β-gal combine to generate a catalytically active species, and fusion is detected via the luminescence that results from substrate processing by the reconstituted β-gal.

**HRC-L-PEG_4_-Chol, QE-L-PEG_4_-Chol**, and **SK-L-PEG_4_-Chol** inhibited D614G S-mediated fusion comparably, with 50% inhibitory concentrations (IC_50_) of 33, 25, and 48 nM, respectively (Table 1). All three peptides were significantly more effective toward SARS-CoV-2 BA.5 relative to SARS-CoV-2 D614G (Table 1). For the BA.5 variant, **HRC-L-PEG_4_-Chol** (IC_50_ of 0.81 nM) was moderately but significantly more potent than either **QE-L-PEG_4_-Chol** (IC_50_ of 7.0 nM) or **SK-L-PEG_4_-Chol** (IC_50_ of 6.2 nM). Each of the lipopeptides displayed little or no toxicity in a cell-based assay (Fig. S4).

**TABLE 1:** IC_50_ and IC_90_ from inhibition of SARS-CoV-2 spike (S)-mediated cell–cell fusion

Lipopeptides	SARS-CoV-2 D614G		SARS-CoV-2 BA.5	
	IC_50_ (nM)	IC_90_ (nM)	IC_50_ (nM)	IC_90_ (nM)
HRC	25 ±± 4	34 ± 9	0.81 ± 0.10	1.9 ± 0.2
QE	33 ± 5	70 ± 10	7.0 ± 2.1	14 ± 4
SK	48 ± 8	96 ± 20	6.2 ± 1.1	14 ± 2
QE-EXT	51 ± 8	90 ± 20	5.6 ± 0.72	13 +/ 2

The final step in the synthesis of the lipopeptide derivatives involves attachment of the PEG_4_–Chol unit to the Cys side chain via nucleophilic displacement. The desired product contains a thioether at the attachment site (Fig. 1C); however, the reaction process inevitably generates a significant amount of the sulfoxide derivative, which apparently results from spontaneous oxidation of the thioether in air. Each sulfoxide lipopeptide by-product could be readily separated from the corresponding thioether lipopeptide via HPLC. It seems possible that the sulfoxide form would be generated in the lung if a lipopeptide were administered via inhalation. We therefore asked whether the sulfoxide form of a lipopeptide (presumably a pair of diastereomers at sulfur) differs in antiviral activity relative to the thioether form. Thioether vs sulfoxide comparisons revealed no impact of this oxidation on fusion-inhibitory activity in any of the three cases (Fig. S5). This result is encouraging from the perspective of *in vivo* applications.

**Fig 5:**
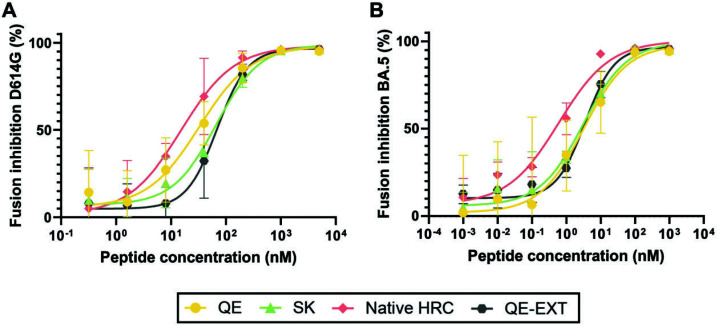
Inhibition of SARS-CoV-2 spike (S)-mediated cell–cell fusion. Fusion inhibitory activity of different peptides against SARS-CoV-2 S variants D614G (**A**) and BA.5 (**B**). The parainfluenza 3 (HPIV3) HRC control was published in references 4 and 1 and is ineffective vs SARS-CoV-2. The percent inhibition is depicted for four SARS-CoV-2 inhibitory peptides at increasing concentrations. The percent fusion inhibition was calculated as the ratio of luminescence in the presence of peptide at a specific concentration (X) to the luminescence in the absence of inhibitor. Percent inhibition = 100 × [1 − (luminescence at X)/(luminescence in the absence of peptide)]. Data in (**A**) and (**B**) are mean ± standard error of the mean (SEM) from three distinct experiments with a four-parameter variable curve.

It was recently reported that an N-terminally extended SARS-CoV-2 HRC peptide, corresponding to S residues 1162–1203, was significantly more potent at inhibiting SARS-CoV-2 fusion in a cell–cell fusion assay and a VSV–SARS-CoV-2 chimera infection assay relative to the HRC peptide we previously employed (S residues 1168–1203) (50). This report motivated us to assess the fusion inhibitory activity of the lipopeptide derived from the N-terminally extended version of **QE**, i.e., **QE-EXT-L-PEG_4_-Chol**. The fusion inhibitory efficacies of **QE-EXT-L-PEG_4_-Chol** and **QE-L-PEG_4_-Chol** were statistically indistinguishable vs D614G (IC_50_ of 51 nM compared with IC_50_ of 33 nM) (Fig. 5A) or BA.5 (IC_50_ of 5.6 nM compared with IC_50_ of 7.0 nM) (Fig. 5B) (Table 1). The control lipopeptide corresponding to the parainfluenza 3 (HPIV3) HRC was shown in References (4) and (1) to be ineffective vs SARS-CoV-2. In contrast to the observation that the extension of the native SARS-CoV-2 HRC peptide increased fusion inhibitor activity (50), we did not observe an analogous effect for the QE HRC peptide (**Fig. S10**).

### Inhibition of SARS-CoV-2 and MERS infection by the designed peptides

**Fig 6:**
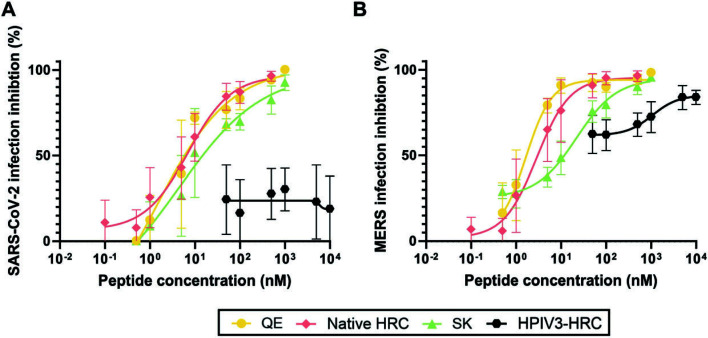
Inhibition of SARS-CoV-2 and MERS viral infection by the designed peptides. Plaque reduction assay with SARS-CoV-2 virus (**A**) or MERS virus (**B**). The QE and SK lipopeptides were assessed alongside the native HRC lipopeptide. This data set included the native HRC and the negative control parainfluenza 3 (HPIV3) HRC published in reference 4 and reproduced here.

We compared the efficacy of the lipopeptides **HRC-L-PEG_4_-Chol**, **QE-L-PEG_4_-Chol**, and **SK-L-PEG_4_-Chol** at inhibiting entry of SARS-CoV-2 into Vero E6 cells using a plaque reduction neutralization test (PRNT). **HRC-L-PEG_4_-Chol** (IC_50_ 6.3 nM; IC_90_ 15 nM; Table 2), **QE-L-PEG_4_-Chol** (IC_50_ 5.9 nM; IC_90_ 11 nM), and **SK-L-PEG_4_-Chol** (IC_50_ 7.7 nM; IC_90_ 14 nM) were similar in potency at inhibiting entry (Fig. 6A). We tested these three lipopeptide also for inhibition of MERS entry. We have previously shown that a lipopeptide derived from the SARS-CoV-2 HRC is effective at inhibiting MERS (4). Figure 6B shows that **HRC-L-PEG_4_-Chol** and **QE-L-PEG_4_-Chol** had similar efficacy against MERS, while **SK-L-PEG_4_-Chol** was modestly less potent. The control lipopeptide HPIV3 HRC published in Reference (4) was shown here in parallel to be relatively ineffective vs SARS-CoV-2 (50% inhibition not obtained) and MERS (IC_50_ ~70 nM).

**TABLE 2:** IC_50_ and IC_90_ from live virus plaque reduction neutralization test

Lipopeptides	SARS-CoV-2		MERS	
	IC_50_ (nM)	IC_90_ (nM)	IC_50_ (nM)	IC_90_ (nM)
HRC	6.3 ± 0.6	15 ± 2	3.5 ± 0.4	7.9 ± 1
QE	5.9 ± 0.6	11 ± 1	2.3 ± 0.3	5.6 ± 0.7
SK	7.7 ± 0.8	14 ± 2	13 ± 2	61 ± 8

## DISCUSSION

This work focused on improving the solubility of peptides used to generate inhibitors of SARS-CoV-2 infection. As discussed above, lipopeptide inhibitors of viral entry could represent a useful contribution in the struggle to protect vulnerable populations from COVID-19. This effort was motivated by the importance of peptide solubility in manufacturing and delivery (51). Our long-term goal is to develop SARS-CoV-2 fusion inhibitors containing backbone modifications that suppress proteolysis, which should lead to prolonged activity *in vivo*. This strategy has been successful with HPIV3 fusion inhibitors (23), and the first step toward this goal was to identify variants of the native HRC sequence with improved solubility.

We employed a design strategy inspired by the antiviral peptide **EK1**, which was derived from the HRC domain of the human coronavirus OC43 (37). Xia et al. generated **EK1** by modifying the native OC43 sequence at positions that are not expected to engage the HRN domain in the six-helix bundle assemble of the post-fusion S state. These modifications enhanced the complement of polar/charged side chains in **EK1** relative to the native OC43 HRC, which was expected to increase solubility. However, the native OC43 HRC residues that are predicted to contact the HRN core in the six-helix bundle assembly differ at many positions from the corresponding residues in the SARS-CoV-2 HRC domain. These differences presumably explain why we found a lipopeptide derived from **EK1** to be substantially less potent at inhibiting fusion mediated by SARS-CoV-2 S relative to the analogous lipopeptide derived from the native SARS-CoV-2 HRC (i.e., **HRC-L-PEG_4_-Chol**) (4).

Our design strategy generated two novel analogues of the native SARS-CoV-2 HRC domain, **QE**, and **SK**. Both contain the native SARS-CoV-2 residues at all positions that contact the trimeric HRN core in the six-helix bundle assembly (PDB 6LXT) (36). However, **QE** contains eight substitutions at solvent-exposed positions relative to **HRC**, and **SK** contains 15 substitutions. When evaluated with derivatives containing a short C-terminal linker segment, both of the designed sequences display significantly enhanced solubility relative to the native HRC. Despite the considerable number of sequence changes in **QE** and **SK** relative to **HRC**, these three peptides form assemblies of comparable stability with the native HRN, presumably six-helix bundles, according to VT-CD data. We solved a **QE** + HRN co-crystal structure that reveals an assembly very similar to that reported for the native HRC + HRN pair (36).

Two assay formats were employed to evaluate the functional consequences of the new designs, one involving cell–cell fusion mediated by SARS-CoV-2 S, and the other involving viral infection of Vero E6 cells. These studies employed lipopeptides derived from **QE**, **SK**, or **HRC**. The fusion assays were conducted with either of two S variants; with D614G, the three lipopeptides were indistinguishable, while with BA.5, the **HRC** lipopeptide displayed a modest advantage relative to the **QE** or **SK** lipopeptide. The three lipopeptides were indistinguishable in their ability to inhibit Vero E6 cell infection (Wuhan strain). The **HRC** and **QE** lipopeptides were comparably potent inhibitors of MERS infection as well, while the **SK** lipopeptide was moderately less potent against this virus. Overall, these results show that the native SARS-CoV-2 HRC domain can be substantially modified to enhance solubility while retaining potent antiviral activity.

The final step in our lipopeptide synthesis method creates a thioether linkage between the peptide and the cholesterol unit, and this process invariably generates the corresponding sulfoxide as a by-product (Fig. S12). This unintended oxidation seems likely to occur *in vivo* as well (52). It is therefore significant that the sulfoxide forms of all three lipopeptides, derived from **HRC**, **QE** or **SK**, match the fusion-inhibitory potencies of the thioether analogues. We used the **QE** system to evaluate another type of modification in which the HRC domain is lengthened by six residues at the N-terminus. Such an extension might lead to additional favorable contacts in the six-helix bundle assembly and therefore enhance antiviral potency; however, in distinction to a recent report that extension of the native HRC segment resulted in dramatically improved antiviral potency (50), we observed no change in the fusion-inhibitory activity for the extended **QE** lipopeptide.

The **QE** sequence introduced here appears to represent a favorable basis for further development intended to deliver a prophylactic agent that could protect vulnerable patient populations from COVID-19. The next step will be to incorporate backbone modifications into the **QE** sequence in an effort to inhibit degradation by proteases *in vivo*. Success in this effort could lead to a significant public health advance.

## MATERIALS AND METHODS

### Peptide synthesis

All peptides were produced by standard Fmoc-based solid-phase synthetic methods using a Liberty Blue microwave-assisted peptide synthesizer. The cholesterol moiety was attached to peptides as previously described (4, 7, 8) via a chemoselective reaction between the thiol group of the C-terminal cysteine residue and a bromoacetyl derivative of cholesterol. More synthetic details and characterization data can be found in the supplemental information (Fig. S15 to S29).

### B-Gal complementation cell-cell fusion assay

To evaluate cell–cell fusion, we modified a fusion assay relying on alpha complementation of B-galactosidase (B-gal) (8). For this assay, cells co-expressing the hACE2 receptor and the omega peptide of B-gal are mixed with cells bearing SARS-Cov-2 glycoprotein S and the alpha peptide of B-gal. The fusion of cells allows for complementation of the alpha and omega peptides of B-gal, and this process continues until fusion is terminated by lysing the cells. The substrate (The Tropix Galacto-Star chemiluminescent reporter assay system, Applied Biosystem) is introduced, and luminescence is quantified on a Cytation 5 (BioTek) or a Tecan M1000. All data were plotted in GraphPad Prism. A nonlinear regression model was applied to the data, yielding a fitted curve.

### *In vitro* cytotoxicity assessment

An MTT (3-[4,5-dimethylthiazole-2-yl]−2,5-diphenyltetrazolium bromide) assay was used to determine the cytotoxicity of lipopeptides **HRC-L-PEG_4_-Chol**, **QE-L-PEG_4_-Chol**, or **SK-L-PEG_4_-Chol** toward HEK 293T cells. The toxicity observed for each lipopeptide was <20% at 5 µM.

### Peptide concentration determination

**QE-L** contains a single residue with an aromatic side chain (Tyr), and **SK-L** contains two Tyr residues. For these peptides, concentration was calculated based on absorbance at 280 nm and a well-established extinction coefficient (ε_280_ = 1,490 M^−1^ cm^−1^ for QE; 2,980 M^−1^ cm^−1^ for SK) (43). The **HRC-L** does not contain an aromatic side chain, and concentration was calculated based on absorbance at 205 nm from the peptide backbone (ε_205_ = 118,820 M^−1^ cm^−1^) (44). It should be noted that absorbance at 205 nm could not be used for measurements with **QE-L** or **SK-L** because there is signal contribution from the UV absorbance of the Tyr side chain (44). Solubility over time was measured in phosphate-buffered saline (PBS) at room temperature by adapting the saturation shake-flask method (53). A 12.5 mg/mL mixture of **HRC-L**, **QE-L**, or **SK-L** in PBS was placed in an Eppendorf tube, vortexed, sonicated, and centrifuged. The tube was allowed to stand at room temperature for 1 h, and then a large portion of the supernatant was transferred to another tube. The absorbance at the appropriate wavelength (280 nm for **QE-L** or **SK-L**, or 205 nm for **HRC-L**) was measured at this point, and again at 18, 72, 144, and 168 h after sample preparation. At each time point, the sample was centrifuged before the absorbance measurement. UPLC analysis demonstrated that all three peptides remained intact during the solubility study (Fig. S13 and 14).

### GRAVY

GRAVY scores are calculated by summing the individual hydropathy scores for each residue, then dividing by the total number of residues in the peptide or protein sequence (42).

### Structure determination

Described in the supplemental information. Details shown in Table S1.

### Circular dichroism

All circular dichroism (CD) experiments were performed on a JASCO J-1500 CD spectrometer. Samples were prepared in 1 mm quartz cuvettes with 10 mM phosphate buffer, pH 7.4. Wavelength scans were collected from 190 to 260 nm with a 1 nm bandwidth, 4 s averaging time, and scanning speed of 50 nm/min. Individual peptides were prepared at 25 µM, and peptide combinations were prepared at 50 µM (25 µM each component). For variable temperature experiments, ellipticity was measured at 222 nm as temperature was raised from 5 °C to 95 °C in 5°C increments with a 5 min equilibration time at each new temperature and a 5 second averaging time for each measurement. All experiments were repeated in triplicate. Data are presented as mean ± standard error.

### Cells

Human embryonic kidney (HEK) 293T cells were grown in Dulbecco’s modified Eagle’s medium (DMEM; Invitrogen; Thermo Fisher Scientific) supplemented with 10% fetal bovine serum (FBS) and antibiotics in 5% CO2. Vero E6 cells (ATCC CRL-1586) were grown in minimum essential medium with Earle’s salts (EMEM; Gibco) supplemented with 6% FBS and antibiotics in 5% CO2.

### Plasmids

The cDNAs coding for hACE2 fused to the fluorescent protein Venus, SARS-CoV-2 S D614G, and SARS-CoV-2 S BA.5 (codon optimized for mammalian expression) were cloned in a modified version of the pCAGGS (with puromycin resistance for selection).

### Viral titration and plaque reduction neutralization assay

As performed previously (4), titers of virus stocks were determined by plaque assay in Vero E6 cells grown in six-well tissue culture plates. Virus stocks were serially diluted 10-fold in PBS, and 0.2 mL of each dilution was inoculated into quadruplicate wells and allowed to adsorb at 37°C for 1 h with rocking every 15 min. Monolayers were rinsed with Dulbecco’s phosphate-buffered saline (DPBS; Corning) and then overlaid with a semisolid medium containing MEM, 5% FBS, antibiotics, and ME agarose (0.6%). Cultures were incubated at 37°C for 3  days and overlaid with DPBS containing neutral red (3.33 g/L; Thermo Fisher Scientific) as a stain (10%), and plaques were counted after 4 to 5 h.

Peptides were tested for inhibitory activity against SARS-CoV-2 and MERS-CoV by plaque reduction neutralization assay. Peptides were serially diluted in molecular biology grade water (1,000  nM or 500 nM through 0.5  nM), each peptide dose was mixed with an equal volume of virus containing 500 particle-forming units (PFU)/mL in MEM, and the peptide/virus mixtures were incubated at 37°C for 1  h. Each peptide dose/virus mixture was inoculated into triplicate wells of Vero E6 cells in six-well plates (0.2  ml per well) and allowed to adsorb at 37°C for 1 h with rocking every 15 min. Monolayers were rinsed with DPBS prior to the addition of medium overlay containing MEM, 5% FBS, antibiotics, and ME agarose (0.6%). Cultures were incubated at 37°C for 2 (SARS-CoV-2) to 3 (MERS CoV) days and overlaid with medium containing neutral red as a stain, and plaques were counted after 4 to 5  h. Virus controls were mixed with sterile water instead of peptide.

### Statistical analysis

Inhibitory concentrations 50% and 90% (IC_50_ and IC_90_ respectively) in fusion assays were calculated with a log variable slope dose versus response curve. Data are mean ± standard error of the mean (SEM) from three distinct experiments with a four-parameter variable curve. All line graphs were compared by two-way ANOVA repeated measures. All statistics were performed with GraphPad Prism V9.

## Data Availability

The crystal PDB file 6X45 is available at https://www.rcsb.org/structure/6X45. All other data are available in the article or supplemental material.

## SUPPLEMENTAL MATERIAL

File S1 jvi.02159-24-s0001.pdf X-ray RMSD calculations.

Supplemental material jvi.02159-24-s0002.docx Supplemental methods, Fig. S1 to S29, and Table S1.

ASM does not own the copyrights to Supplemental Material that may be linked to, or accessed through, an article. The authors have granted ASM a non-exclusive, world-wide license to publish the Supplemental Material files. Please contact the corresponding author directly for reuse.
