# Supplementary material for: Enhancing the solubility of SARS-CoV-2 inhibitors to increase future prospects for clinical development
Source: J Virol. 2025 Feb 4;99(3):e02159-24. doi: 10.1128/jvi.02159-24 (PMC11915835; doi:10.1128/jvi.02159-24)
Supplement: File S1 — X-ray RMSD calculations. [file jvi.02159-24-s0001.pdf]

kuhn_6lx6_6x45_alignment

June 17, 2024

[590]:
```
import gemmi
import matplotlib.pyplot as plt
import matplotlib.image as mpimg
from fuzzysearch import find_near_matches
from Bio.Align import PairwiseAligner
from pprint import pprint
from collections import OrderedDict
```

[355]:
```
!wget https://files.rcsb.org/download/6x45.pdb
!wget https://files.rcsb.org/download/6lxt.pdb
```

```
--2024-06-16 16:55:11--  https://files.rcsb.org/download/6x45.pdb
Resolving proxy.sdcc.bnl.local (proxy.sdcc.bnl.local)… 130.199.148.95
Connecting to proxy.sdcc.bnl.local
(proxy.sdcc.bnl.local)|130.199.148.95|:3128… connected.
Proxy request sent, awaiting response… 200 OK
Length: unspecified [application/octet-stream]
Saving to: ‘6x45.pdb.8’

6x45.pdb.8              [ <=>                   ] 507.44K  --.-KB/s    in 0.05s

2024-06-16 16:55:11 (10.9 MB/s) - ‘6x45.pdb.8’ saved [519615]

--2024-06-16 16:55:11--  https://files.rcsb.org/download/6lxt.pdb
Resolving proxy.sdcc.bnl.local (proxy.sdcc.bnl.local)… 130.199.148.95
Connecting to proxy.sdcc.bnl.local
(proxy.sdcc.bnl.local)|130.199.148.95|:3128… connected.
Proxy request sent, awaiting response… 200 OK
Length: unspecified [application/octet-stream]
Saving to: ‘6lxt.pdb.6’

6lxt.pdb.6              [ <=>                   ] 899.70K  --.-KB/s    in 0.06s

2024-06-16 16:55:11 (15.6 MB/s) - ‘6lxt.pdb.6’ saved [921294]
```

1 6x45 (6 chains, peptide coiled coil bundle) -> 6lxt (3 chains, hairpin dimer)

1.1 Overview

We need to map the CHR and NHR peptides from 6x45 to corresponding segments in 6lxt. 6lxt is a trimer of hairpin dimers and 6x45 is a bundle of 6 peptides. Therefore a 6x45 chr and 6x45 nhr will map to one 6lxt chain, we'll use a sequence alignment and map segments that give the lowest RMSD. The 6HB assembly has three-fold symmetry so there are technically 3 sets of comparisons that could be made. This mapping is required to define correspondences between atoms to compute the RMSD between these similar assemblies. We will use the following RMSD definition which is the default in pymol and the QCP algorithm (https://doi.org/10.1002/jcc.21439). ### RMSD RMSD $= \sqrt{\frac{\sum_i^N (x_i - x_i')^2 + (y_i - y_i')^2 + (z_i - z_i')^2}{N}}$

1.1.1 Results

For mapping 6x45 6HB -> 6lxt 6HB (chains D,E,F) RMSD = 0.68. For mapping 6x45 CHR -> 6lxt 6HB (chains D,E,F) RMSD = {0.51, 0.69, 1.02} Angstroms.

[426]:
```
x45 = gemmi.read_structure('/nsls2/users/dkreitler/6x45.pdb')
lxt = gemmi.read_structure('/nsls2/users/dkreitler/6lxt.pdb')
```

[559]:
```
for c in x45[0]: print(f'6x45 chain {c.name} has {len(c)} residues')
```

```
6x45 chain E has 34 residues
6x45 chain D has 39 residues
6x45 chain C has 54 residues
6x45 chain F has 34 residues
6x45 chain B has 57 residues
6x45 chain A has 51 residues
```

[555]:
```
def superimpose_atoms(atoms1: list, atoms2: list):
    return gemmi.superpose_positions(atoms1, atoms2).rmsd

def match_seq(
    chain1: gemmi.ResidueSpan,
    chain2: gemmi.ResidueSpan,
    atom_names: list=['CA'],
):

    if len(chain1) <= len(chain2):
        short_chain = chain1
        long_chain = chain2
    else:
        short_chain = chain2
        long_chain = chain1
```

```python
    subseq = short_chain.get_polymer().make_one_letter_sequence().
 ↪replace('-','')
    seq = long_chain.get_polymer().make_one_letter_sequence().replace('-','')
    aligner = PairwiseAligner()
    aligner.mode = 'global'
    aligner.extend_gap_score = 0.2
    aligner.open_gap_score = -1
    aligner.match_score = 2
    alignments = aligner.align(seq, subseq)

    # there should be no gaps in target or query
    for alignment in alignments:
        if alignment.aligned.size == 4:
            print(alignment)
            alignment_ = alignment.aligned.reshape(2,2)
            short_atoms = [
                res.sole_atom(a) for res in short_chain[alignment_[1,0]:
 ↪(alignment_[1,1])] for a in atom_names
            ]
            long_atoms = [
                res.sole_atom(a) for res in long_chain[alignment_[0,0]:
 ↪(alignment_[0,1])] for a in atom_names
            ]
            break

    if alignment_ is None:
        raise ValueError('fragmented alignment')

    assert len(short_atoms) == len(long_atoms)
    return [a.pos for a in short_atoms], [a.pos for a in long_atoms]

def find_correspondence(set1: set, set2: set):

    #arbitrarily define chain 1 as reference and best fit in list2 as first␣
 ↪correspondence
    rmsd = 1E6
    chain_map = OrderedDict()
    ref_chain = set1.pop()
    for c in set2:
        rmsd_ = superimpose_atoms(*match_seq(ref_chain, c))
        if rmsd_ < rmsd:
            rmsd = rmsd_
            print(rmsd)
            chain_map[ref_chain] = c
    while set1:
        new_chain = set1.pop()
        m1_mapped, m2_mapped = [], []
```

```
        for k in chain_map.keys():
            m1, m2 = match_seq(chain_map[k], k)
            m1_mapped += m1
            m2_mapped += m2

        rmsd = 1E6
        for c in set2:
            m1, m2 = match_seq(new_chain, c)
            m1 = m1 + m1_mapped
            m2 = m2 + m2_mapped
            rmsd_ = superimpose_atoms(m1, m2)
            if rmsd_ < rmsd:
                rmsd = rmsd_
                print(f'RMSD: {rmsd}, matched {len(m1)} atoms with {len(m2)}␣
↪atoms')
                chain_map[new_chain] = c

    return chain_map

chain_map = find_correspondence([x45[0][k] for k in ['A','B','C','E','D','F']],␣
↪[lxt[0][k] for k in ['D','E','F']])
#kwargs = {'max_substitutions': 15, 'max_insertions': 15, 'max_deletions':0,␣
↪'max_l_dist':20}
#m = match_seq(x45[0]['E'], lxt[0]['D'])
#print(m.rmsd)
```

```
target            0 TQNVLYENQKLIANQFNSAIGKIQDSLSSTASALGKLQDVVNQNAQALNTLVKQLSSNFG
                  0 ------------------------------------------------------------
query             0 ------------------------------------------------------------

target           60 AISSVLNDILSRLDKDVDLGDISGINASVVNIQKEIDRLNEVAKNLNESL---- 110
                 60 --------------------|||.||||||||..||..|.||||.|.|||---- 114
query             0 --------------------DISQINASVVNIEYEIKKLEEVAKKLEESLIDLQ  34

0.5136615622801634
target            0 VLYENQKLIANQFNSAIGKIQDSLSSTASALGKLQDVVNQNAQALNTLVKQLSSNFGAIS
                  0 ------------------------------------------------------------
query             0 ------------------------------------------------------------

target           60 SVLNDILSRLDKVEDVDLGDISGINASVVNIQKEIDRLNEVAKNLNESLIDLQE 114
                 60 -------------------|||.||||||||..||..|.||||.|.|||||||- 114
query             0 -------------------DISQINASVVNIEYEIKKLEEVAKKLEESLIDLQ-  34

target            0 QNVLYENQKLIANQFNSAIGKIQDSLSSTASALGKLQDVVNQNAQALNTLVKQLSSNFGA
                  0 ------------------------------------------------------------
query             0 ------------------------------------------------------------
```

```
target           60 ISSVLNDILSRLDKVELGDISGINASVVNIQKEIDRLNEVAKNLNESLIDLQE 113
                 60 ------------------|||.||||||||..||..|.||||.|.|||||||- 113
query             0 ------------------DISQINASVVNIEYEIKKLEEVAKKLEESLIDLQ-  34

target            0 TQNVLYENQKLIANQFNSAIGKIQDSLSSTASALGKLQDVVNQNAQALNTLVKQLSSNFG
                  0 ------------------------------------------------------------
query             0 ------------------------------------------------------------

target           60 AISSVLNDILSRLDKDVDLGDISGINASVVNIQKEIDRLNEVAKNLNESL---- 110
                 60 --------------------|||.||||||||..||..|.||||.|.|||---- 114
query             0 --------------------DISQINASVVNIEYEIKKLEEVAKKLEESLIDLQ  34

target            0 TQNVLYENQKLIANQFNSAIGKIQDSLSSTASALGKLQDVVNQNAQALNTLVKQLSSNFG
                  0 ------------------------------------------------------------
query             0 ------------------------------------------------------------

target           60 AISSVLNDILSRLDKDVDLGDISGINASVVNIQKEIDRLNEVAKNLNESL-------
110
                 60 --------------------|||.||||||||..||..|.||||.|.|||-------
117
query             0 --------------------DISQINASVVNIEYEIKKLEEVAKKLEESLIDLQELX
37

RMSD: 9.441870162869805, matched 60 atoms with 60 atoms
target            0 VLYENQKLIANQFNSAIGKIQDSLSSTASALGKLQDVVNQNAQALNTLVKQLSSNFGAIS
                  0 ------------------------------------------------------------
query             0 ------------------------------------------------------------

target           60 SVLNDILSRLDKVEDVDLGDISGINASVVNIQKEIDRLNEVAKNLNESLIDLQE-- 114
                 60 -------------------|||.||||||||..||..|.||||.|.||||||||-- 116
query             0 -------------------DISQINASVVNIEYEIKKLEEVAKKLEESLIDLQELX  37

RMSD: 0.6556797074729303, matched 65 atoms with 65 atoms
target            0 QNVLYENQKLIANQFNSAIGKIQDSLSSTASALGKLQDVVNQNAQALNTLVKQLSSNFGA
                  0 ------------------------------------------------------------
query             0 ------------------------------------------------------------

target           60 ISSVLNDILSRLDKVELGDISGINASVVNIQKEIDRLNEVAKNLNESLIDLQE-- 113
                 60 ------------------|||.||||||||..||..|.||||.|.||||||||-- 115
query             0 ------------------DISQINASVVNIEYEIKKLEEVAKKLEESLIDLQELX  37

target            0 TQNVLYENQKLIANQFNSAIGKIQDSLSSTASALGKLQDVVNQNAQALNTLVKQLSSNFG
                  0 ------------------------------------------------------------
query             0 ------------------------------------------------------------

target           60 AISSVLNDILSRLDKDVDLGDISGINASVVNIQKEIDRLNEVAKNLNESL---- 110
                 60 --------------------|||.||||||||..||..|.||||.|.|||---- 114
query             0 --------------------DISQINASVVNIEYEIKKLEEVAKKLEESLIDLQ  34
```

```
target            0 VLYENQKLIANQFNSAIGKIQDSLSSTASALGKLQDVVNQNAQALNTLVKQLSSNFGAIS
                  0 ------------------------------------------------------------
query             0 ------------------------------------------------------------

target           60 SVLNDILSRLDKVEDVDLGDISGINASVVNIQKEIDRLNEVAKNLNESLIDLQE-- 114
                 60 -------------------|||.||||||||..||..|.||||.|.||||||||-- 116
query             0 -------------------DISQINASVVNIEYEIKKLEEVAKKLEESLIDLQELX  37

target            0 TQNVLYENQKLIANQFNSAIGKIQDSLSSTASALGKLQDVVNQNAQALNTLVKQLSSNFG
                  0 ------------------------------------------------------------
query             0 ------------------------------------------------------------

target           60 AISSVLNDILSRLDKDVDLGDISGINASVVNIQKEIDRLNEVAKNLNESL---- 110
                 60 --------------------|||.||||||||..||..|.||||.|.|||---- 114
query             0 --------------------DISQINASVVNIEYEIKKLEEVAKKLEESLIDLQ  34

RMSD: 7.668643213744839, matched 95 atoms with 95 atoms
target            0 VLYENQKLIANQFNSAIGKIQDSLSSTASALGKLQDVVNQNAQALNTLVKQLSSNFGAIS
                  0 ------------------------------------------------------------
query             0 ------------------------------------------------------------

target           60 SVLNDILSRLDKVEDVDLGDISGINASVVNIQKEIDRLNEVAKNLNESLIDLQE 114
                 60 -------------------|||.||||||||..||..|.||||.|.|||||||- 114
query             0 -------------------DISQINASVVNIEYEIKKLEEVAKKLEESLIDLQ-  34

target            0 QNVLYENQKLIANQFNSAIGKIQDSLSSTASALGKLQDVVNQNAQALNTLVKQLSSNFGA
                  0 ------------------------------------------------------------
query             0 ------------------------------------------------------------

target           60 ISSVLNDILSRLDKVELGDISGINASVVNIQKEIDRLNEVAKNLNESLIDLQE 113
                 60 ------------------|||.||||||||..||..|.||||.|.|||||||- 113
query             0 ------------------DISQINASVVNIEYEIKKLEEVAKKLEESLIDLQ-  34

RMSD: 0.8335147934598741, matched 99 atoms with 99 atoms
target            0 TQNVLYENQKLIANQFNSAIGKIQDSLSSTASALGKLQDVVNQNAQALNTLVKQLSSNFG
                  0 ------------------------------------------------------------
query             0 ------------------------------------------------------------

target           60 AISSVLNDILSRLDKDVDLGDISGINASVVNIQKEIDRLNEVAKNLNESL---- 110
                 60 --------------------|||.||||||||..||..|.||||.|.|||---- 114
query             0 --------------------DISQINASVVNIEYEIKKLEEVAKKLEESLIDLQ  34

target            0 VLYENQKLIANQFNSAIGKIQDSLSSTASALGKLQDVVNQNAQALNTLVKQLSSNFGAIS
                  0 ------------------------------------------------------------
query             0 ------------------------------------------------------------

target           60 SVLNDILSRLDKVEDVDLGDISGINASVVNIQKEIDRLNEVAKNLNESLIDLQE-- 114
```

```
                 60 -------------------|||.||||||||..||..|.||||.|.||||||||-- 116
query             0 -------------------DISQINASVVNIEYEIKKLEEVAKKLEESLIDLQELX  37

target            0 QNVLYENQKLIANQFNSAIGKIQDSLSSTASALGKLQDVVNQNAQALNTLVKQLSSNFGA
                  0 ------------------------------------------------------------
query             0 ------------------------------------------------------------

target           60 ISSVLNDILSRLDKVELGDISGINASVVNIQKEIDRLNEVAKNLNESLIDLQE 113
                 60 ------------------|||.||||||||..||..|.||||.|.|||||||- 113
query             0 ------------------DISQINASVVNIEYEIKKLEEVAKKLEESLIDLQ-  34

target            0 TQNVLYENQKLIANQFNSAIGKIQDSLSSTASALGKLQDVVNQNAQALNTLVKQLSSNFG
                  0 --||||||||||||||||||||||||||||||||||||||||||||||||||||------
query             0 --NVLYENQKLIANQFNSAIGKIQDSLSSTASALGKLQDVVNQNAQALNTLVKQ------

target           60 AISSVLNDILSRLDKDVDLGDISGINASVVNIQKEIDRLNEVAKNLNESL 110
                 60 -------------------------------------------------- 110
query            52 --------------------------------------------------  52

RMSD: 0.7446450513962493, matched 151 atoms with 151 atoms
target            0 -VLYENQKLIANQFNSAIGKIQDSLSSTASALGKLQDVVNQNAQALNTLVKQLSSNFGAI
                  0 -|||||||||||||||||||||||||||||||||||||||||||||||||||--------
query             0 NVLYENQKLIANQFNSAIGKIQDSLSSTASALGKLQDVVNQNAQALNTLVKQ--------

target           59 SSVLNDILSRLDKVEDVDLGDISGINASVVNIQKEIDRLNEVAKNLNESLIDLQE 114
                 60 ------------------------------------------------------- 115
query            52 -------------------------------------------------------  52

target            0 QNVLYENQKLIANQFNSAIGKIQDSLSSTASALGKLQDVVNQNAQALNTLVKQLSSNFGA
                  0 -||||||||||||||||||||||||||||||||||||||||||||||||||||-------
query             0 -NVLYENQKLIANQFNSAIGKIQDSLSSTASALGKLQDVVNQNAQALNTLVKQ-------

target           60 ISSVLNDILSRLDKVELGDISGINASVVNIQKEIDRLNEVAKNLNESLIDLQE 113
                 60 ----------------------------------------------------- 113
query            52 -----------------------------------------------------  52

target            0 TQNVLYENQKLIANQFNSAIGKIQDSLSSTASALGKLQDVVNQNAQALNTLVKQLSSNFG
                  0 ------------------------------------------------------------
query             0 ------------------------------------------------------------

target           60 AISSVLNDILSRLDKDVDLGDISGINASVVNIQKEIDRLNEVAKNLNESL---- 110
                 60 --------------------|||.||||||||..||..|.||||.|.|||---- 114
query             0 --------------------DISQINASVVNIEYEIKKLEEVAKKLEESLIDLQ  34

target            0 VLYENQKLIANQFNSAIGKIQDSLSSTASALGKLQDVVNQNAQALNTLVKQLSSNFGAIS
                  0 ------------------------------------------------------------
query             0 ------------------------------------------------------------
```

```
target           60 SVLNDILSRLDKVEDVDLGDISGINASVVNIQKEIDRLNEVAKNLNESLIDLQE-- 114
                 60 -------------------|||.||||||||..||..|.||||.|.||||||||-- 116
query             0 -------------------DISQINASVVNIEYEIKKLEEVAKKLEESLIDLQELX  37

target            0 QNVLYENQKLIANQFNSAIGKIQDSLSSTASALGKLQDVVNQNAQALNTLVKQLSSNFGA
                  0 ------------------------------------------------------------
query             0 ------------------------------------------------------------

target           60 ISSVLNDILSRLDKVELGDISGINASVVNIQKEIDRLNEVAKNLNESLIDLQE 113
                 60 ------------------|||.||||||||..||..|.||||.|.|||||||- 113
query             0 ------------------DISQINASVVNIEYEIKKLEEVAKKLEESLIDLQ-  34

target            0 TQNVLYENQKLIANQFNSAIGKIQDSLSSTASALGKLQDVVNQNAQALNTLVKQLSSNFG
                  0 --||||||||||||||||||||||||||||||||||||||||||||||||||||------
query             0 --NVLYENQKLIANQFNSAIGKIQDSLSSTASALGKLQDVVNQNAQALNTLVKQ------

target           60 AISSVLNDILSRLDKDVDLGDISGINASVVNIQKEIDRLNEVAKNLNESL 110
                 60 -------------------------------------------------- 110
query            52 --------------------------------------------------  52

target            0 TQNVLYENQKLIANQFNSAIGKIQDSLSSTASALGKLQDVVNQNAQALNTLVKQLSSNFG
                  0 --|||||||||||||||||||||||||||||||||||||||||||||||||||||-----
query             0 --NVLYENQKLIANQFNSAIGKIQDSLSSTASALGKLQDVVNQNAQALNTLVKQL-----

target           60 AISSVLNDILSRLDKDVDLGDISGINASVVNIQKEIDRLNEVAKNLNESL 110
                 60 -------------------------------------------------- 110
query            53 --------------------------------------------------  53

RMSD: 5.589007876247789, matched 204 atoms with 204 atoms
target            0 -VLYENQKLIANQFNSAIGKIQDSLSSTASALGKLQDVVNQNAQALNTLVKQLSSNFGAI
                  0 -||||||||||||||||||||||||||||||||||||||||||||||||||||-------
query             0 NVLYENQKLIANQFNSAIGKIQDSLSSTASALGKLQDVVNQNAQALNTLVKQL-------

target           59 SSVLNDILSRLDKVEDVDLGDISGINASVVNIQKEIDRLNEVAKNLNESLIDLQE 114
                 60 ------------------------------------------------------- 115
query            53 -------------------------------------------------------  53

RMSD: 5.28856198109047, matched 203 atoms with 203 atoms
target            0 QNVLYENQKLIANQFNSAIGKIQDSLSSTASALGKLQDVVNQNAQALNTLVKQLSSNFGA
                  0 -|||||||||||||||||||||||||||||||||||||||||||||||||||||||------
query             0 -NVLYENQKLIANQFNSAIGKIQDSLSSTASALGKLQDVVNQNAQALNTLVKQL------

target           60 ISSVLNDILSRLDKVELGDISGINASVVNIQKEIDRLNEVAKNLNESLIDLQE 113
                 60 ----------------------------------------------------- 113
query            53 -----------------------------------------------------  53

RMSD: 0.6796115552751139, matched 204 atoms with 204 atoms
target            0 TQNVLYENQKLIANQFNSAIGKIQDSLSSTASALGKLQDVVNQNAQALNTLVKQLSSNFG
```

```
                 0 ------------------------------------------------------------
query            0 ------------------------------------------------------------

target          60 AISSVLNDILSRLDKDVDLGDISGINASVVNIQKEIDRLNEVAKNLNESL---- 110
                60 --------------------|||.||||||||..||..|.||||.|.|||---- 114
query            0 --------------------DISQINASVVNIEYEIKKLEEVAKKLEESLIDLQ  34

target           0 VLYENQKLIANQFNSAIGKIQDSLSSTASALGKLQDVVNQNAQALNTLVKQLSSNFGAIS
                 0 ------------------------------------------------------------
query            0 ------------------------------------------------------------

target          60 SVLNDILSRLDKVEDVDLGDISGINASVVNIQKEIDRLNEVAKNLNESLIDLQE-- 114
                60 -------------------|||.||||||||..||..|.||||.|.||||||||-- 116
query            0 -------------------DISQINASVVNIEYEIKKLEEVAKKLEESLIDLQELX  37

target           0 QNVLYENQKLIANQFNSAIGKIQDSLSSTASALGKLQDVVNQNAQALNTLVKQLSSNFGA
                 0 ------------------------------------------------------------
query            0 ------------------------------------------------------------

target          60 ISSVLNDILSRLDKVELGDISGINASVVNIQKEIDRLNEVAKNLNESLIDLQE 113
                60 ------------------|||.||||||||..||..|.||||.|.|||||||- 113
query            0 ------------------DISQINASVVNIEYEIKKLEEVAKKLEESLIDLQ-  34

target           0 TQNVLYENQKLIANQFNSAIGKIQDSLSSTASALGKLQDVVNQNAQALNTLVKQLSSNFG
                 0 --||||||||||||||||||||||||||||||||||||||||||||||||||||------
query            0 --NVLYENQKLIANQFNSAIGKIQDSLSSTASALGKLQDVVNQNAQALNTLVKQ------

target          60 AISSVLNDILSRLDKDVDLGDISGINASVVNIQKEIDRLNEVAKNLNESL 110
                60 -------------------------------------------------- 110
query           52 --------------------------------------------------  52

target           0 QNVLYENQKLIANQFNSAIGKIQDSLSSTASALGKLQDVVNQNAQALNTLVKQLSSNFGA
                 0 -|||||||||||||||||||||||||||||||||||||||||||||||||||||------
query            0 -NVLYENQKLIANQFNSAIGKIQDSLSSTASALGKLQDVVNQNAQALNTLVKQL------

target          60 ISSVLNDILSRLDKVELGDISGINASVVNIQKEIDRLNEVAKNLNESLIDLQE 113
                60 ----------------------------------------------------- 113
query           53 -----------------------------------------------------  53

target           0 TQNVLYENQKLIANQFNSAIGKIQDSLSSTASALGKLQDVVNQNAQALNTLVKQLSSNFG
                 0 ----|||||||||||||||||||||||||||||||||||||||||||||||||||-----
query            0 ----LYENQKLIANQFNSAIGKIQDSLSSTASALGKLQDVVNQNAQALNTLVKQL-----

target          60 AISSVLNDILSRLDKDVDLGDISGINASVVNIQKEIDRLNEVAKNLNESL 110
                60 -------------------------------------------------- 110
query           51 --------------------------------------------------  51

RMSD: 4.870395625973949, matched 255 atoms with 255 atoms
```

```
target            0 VLYENQKLIANQFNSAIGKIQDSLSSTASALGKLQDVVNQNAQALNTLVKQLSSNFGAIS
                  0 -|||||||||||||||||||||||||||||||||||||||||||||||||||--------
query             0 -LYENQKLIANQFNSAIGKIQDSLSSTASALGKLQDVVNQNAQALNTLVKQL--------

target           60 SVLNDILSRLDKVEDVDLGDISGINASVVNIQKEIDRLNEVAKNLNESLIDLQE 114
                 60 ------------------------------------------------------ 114
query            51 ------------------------------------------------------  51

RMSD: 0.6807631189786063, matched 255 atoms with 255 atoms
target            0 QNVLYENQKLIANQFNSAIGKIQDSLSSTASALGKLQDVVNQNAQALNTLVKQLSSNFGA
                  0 ---|||||||||||||||||||||||||||||||||||||||||||||||||||------
query             0 ---LYENQKLIANQFNSAIGKIQDSLSSTASALGKLQDVVNQNAQALNTLVKQL------

target           60 ISSVLNDILSRLDKVELGDISGINASVVNIQKEIDRLNEVAKNLNESLIDLQE 113
                 60 ----------------------------------------------------- 113
query            51 -----------------------------------------------------  51
```

[565]:
```python
chain_map
```

[565]:
```
OrderedDict([(<gemmi.Chain F with 34 res>, <gemmi.Chain D with 110 res>),
             (<gemmi.Chain D with 39 res>, <gemmi.Chain E with 114 res>),
             (<gemmi.Chain E with 34 res>, <gemmi.Chain F with 115 res>),
             (<gemmi.Chain C with 54 res>, <gemmi.Chain D with 110 res>),
             (<gemmi.Chain B with 57 res>, <gemmi.Chain F with 115 res>),
             (<gemmi.Chain A with 51 res>, <gemmi.Chain E with 114 res>)])
```

2 mappings

6x45 / 6lxt ## F -> D, chr ## D -> E, chr ## E -> F, chr ## C -> D, nhr ## B -> F, nhr ## A -> E, nhr

2.1 for 255 C-alpha atoms in 6HB, RMSD = 0.68

2.2 for found correspondences

get sets of atoms that are being aligned, designated m1 and m2

[566]:
```python
m1, m2 = [], []
for k in chain_map.keys():
    m1_, m2_ = match_seq(chain_map[k], k)
    m1 += m1_
    m2 += m2_
```

```
target            0 TQNVLYENQKLIANQFNSAIGKIQDSLSSTASALGKLQDVVNQNAQALNTLVKQLSSNFG
                  0 ------------------------------------------------------------
query             0 ------------------------------------------------------------
```

```
target           60 AISSVLNDILSRLDKDVDLGDISGINASVVNIQKEIDRLNEVAKNLNESL---- 110
                 60 --------------------|||.||||||||..||..|.||||.|.|||---- 114
query             0 --------------------DISQINASVVNIEYEIKKLEEVAKKLEESLIDLQ  34

target            0 VLYENQKLIANQFNSAIGKIQDSLSSTASALGKLQDVVNQNAQALNTLVKQLSSNFGAIS
                  0 ------------------------------------------------------------
query             0 ------------------------------------------------------------

target           60 SVLNDILSRLDKVEDVDLGDISGINASVVNIQKEIDRLNEVAKNLNESLIDLQE-- 114
                 60 -------------------|||.||||||||..||..|.||||.|.||||||||-- 116
query             0 -------------------DISQINASVVNIEYEIKKLEEVAKKLEESLIDLQELX  37

target            0 QNVLYENQKLIANQFNSAIGKIQDSLSSTASALGKLQDVVNQNAQALNTLVKQLSSNFGA
                  0 ------------------------------------------------------------
query             0 ------------------------------------------------------------

target           60 ISSVLNDILSRLDKVELGDISGINASVVNIQKEIDRLNEVAKNLNESLIDLQE 113
                 60 ------------------|||.||||||||..||..|.||||.|.|||||||- 113
query             0 ------------------DISQINASVVNIEYEIKKLEEVAKKLEESLIDLQ-  34

target            0 TQNVLYENQKLIANQFNSAIGKIQDSLSSTASALGKLQDVVNQNAQALNTLVKQLSSNFG
                  0 --||||||||||||||||||||||||||||||||||||||||||||||||||||------
query             0 --NVLYENQKLIANQFNSAIGKIQDSLSSTASALGKLQDVVNQNAQALNTLVKQ------

target           60 AISSVLNDILSRLDKDVDLGDISGINASVVNIQKEIDRLNEVAKNLNESL 110
                 60 -------------------------------------------------- 110
query            52 --------------------------------------------------  52

target            0 QNVLYENQKLIANQFNSAIGKIQDSLSSTASALGKLQDVVNQNAQALNTLVKQLSSNFGA
                  0 -|||||||||||||||||||||||||||||||||||||||||||||||||||||------
query             0 -NVLYENQKLIANQFNSAIGKIQDSLSSTASALGKLQDVVNQNAQALNTLVKQL------

target           60 ISSVLNDILSRLDKVELGDISGINASVVNIQKEIDRLNEVAKNLNESLIDLQE 113
                 60 ----------------------------------------------------- 113
query            53 -----------------------------------------------------  53

target            0 VLYENQKLIANQFNSAIGKIQDSLSSTASALGKLQDVVNQNAQALNTLVKQLSSNFGAIS
                  0 -|||||||||||||||||||||||||||||||||||||||||||||||||||--------
query             0 -LYENQKLIANQFNSAIGKIQDSLSSTASALGKLQDVVNQNAQALNTLVKQL--------

target           60 SVLNDILSRLDKVEDVDLGDISGINASVVNIQKEIDRLNEVAKNLNESLIDLQE 114
                 60 ------------------------------------------------------ 114
query            51 ------------------------------------------------------  51
```

[571]:

```
#generate superposition
sup = gemmi.superpose_positions(m2, m1)
```

[572]:
```
sup.rmsd
```

[572]: 0.680763118978774

[573]:
```
sup.transform.mat
```

[573]:
```
<gemmi.Mat33 [0.841254, -0.539309, 0.0379086]
             [-0.540273, -0.836035, 0.0956605]
             [-0.0198976, -0.100956, -0.994692]>
```

[574]:
```
x45_copy = x45.clone()
```

[575]:
```
x45_copy[0].transform_pos_and_adp(sup.transform)
```

[576]:
```
x45_copy.write_pdb('6x45_transform.pdb')
```

[592]:
```
transformed_6x45 = mpimg.imread('6x45_transform.png')
plt.imshow(transformed_6x45)
```

[592]: <matplotlib.image.AxesImage at 0x2af503888f10>

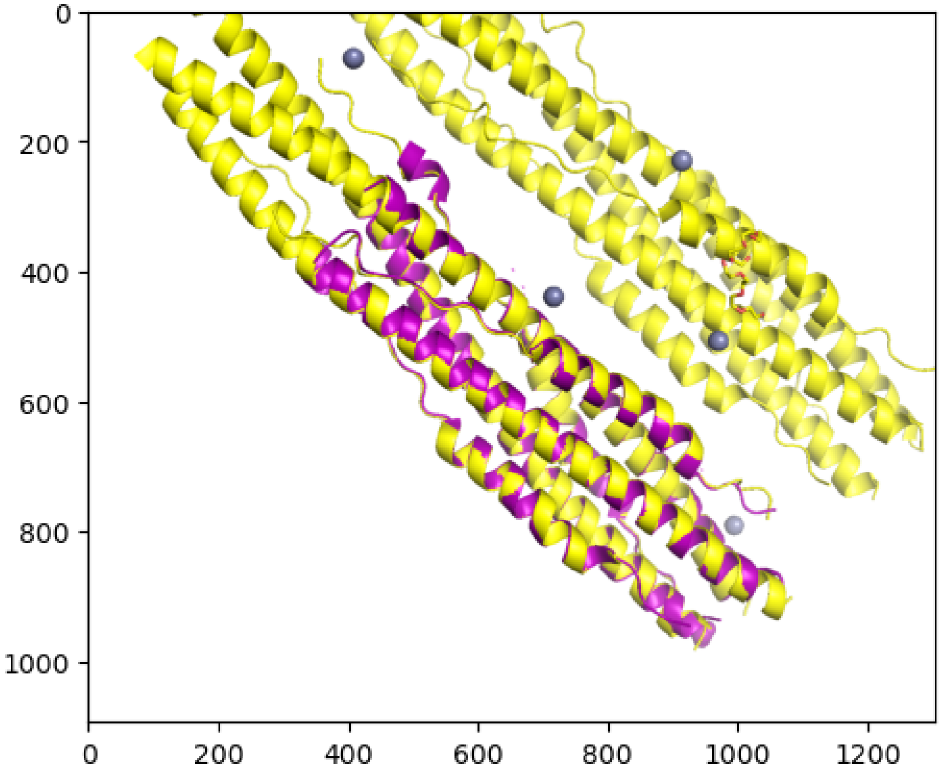

2.3 Now match CHR

2.4 6x45 chain F -> 6lxt chain D

2.4.1 RMSD = 0.51 Angstroms

2.5 6x45 chain D -> 6lxt chain E

2.5.1 RMSD = 0.69 Angstroms

2.6 6x45 chain E -> 6lxt chain F

2.6.1 RMSD = 1.02 Angstroms

[593]:
```
find_correspondence(set([x45[0]['F']]),set([lxt[0][k] for k in ['D','E','F']]))
```

```
target            0 TQNVLYENQKLIANQFNSAIGKIQDSLSSTASALGKLQDVVNQNAQALNTLVKQLSSNFG
                  0 ------------------------------------------------------------
query             0 ------------------------------------------------------------

target           60 AISSVLNDILSRLDKDVDLGDISGINASVVNIQKEIDRLNEVAKNLNESL---- 110
                 60 --------------------|||.||||||||..||..|.||||.|.|||---- 114
query             0 --------------------DISQINASVVNIEYEIKKLEEVAKKLEESLIDLQ  34

0.5136615622801634
target            0 QNVLYENQKLIANQFNSAIGKIQDSLSSTASALGKLQDVVNQNAQALNTLVKQLSSNFGA
                  0 ------------------------------------------------------------
query             0 ------------------------------------------------------------

target           60 ISSVLNDILSRLDKVELGDISGINASVVNIQKEIDRLNEVAKNLNESLIDLQE 113
                 60 ------------------|||.||||||||..||..|.||||.|.|||||||- 113
query             0 ------------------DISQINASVVNIEYEIKKLEEVAKKLEESLIDLQ-  34

target            0 VLYENQKLIANQFNSAIGKIQDSLSSTASALGKLQDVVNQNAQALNTLVKQLSSNFGAIS
                  0 ------------------------------------------------------------
query             0 ------------------------------------------------------------

target           60 SVLNDILSRLDKVEDVDLGDISGINASVVNIQKEIDRLNEVAKNLNESLIDLQE 114
                 60 -------------------|||.||||||||..||..|.||||.|.|||||||- 114
query             0 -------------------DISQINASVVNIEYEIKKLEEVAKKLEESLIDLQ-  34
```

[593]: OrderedDict([(<gemmi.Chain F with 34 res>, <gemmi.Chain D with 110 res>)])

[602]:
```
chr1, chr2 = match_seq(x45[0]['F'], lxt[0]['D'])
print(f'{len(chr1)} atoms aligned')
superimpose_atoms(chr1, chr2)
```

```
target            0 TQNVLYENQKLIANQFNSAIGKIQDSLSSTASALGKLQDVVNQNAQALNTLVKQLSSNFG
                  0 ------------------------------------------------------------
query             0 ------------------------------------------------------------
```

```
target            60 AISSVLNDILSRLDKDVDLGDISGINASVVNIQKEIDRLNEVAKNLNESL---- 110
                  60 --------------------|||.||||||||..||..|.||||.|.|||---- 114
query              0 --------------------DISQINASVVNIEYEIKKLEEVAKKLEESLIDLQ  34

30 atoms aligned
```

[602]: 0.5136615622801634

[600]:
```python
chr1, chr2 = match_seq(x45[0]['D'], lxt[0]['E'])
print(f'{len(chr1)} atoms aligned')
superimpose_atoms(chr1, chr2)
```

```
target             0 VLYENQKLIANQFNSAIGKIQDSLSSTASALGKLQDVVNQNAQALNTLVKQLSSNFGAIS
                   0 ------------------------------------------------------------
query              0 ------------------------------------------------------------

target            60 SVLNDILSRLDKVEDVDLGDISGINASVVNIQKEIDRLNEVAKNLNESLIDLQE-- 114
                  60 -------------------|||.||||||||..||..|.||||.|.||||||||-- 116
query              0 -------------------DISQINASVVNIEYEIKKLEEVAKKLEESLIDLQELX  37

35 atoms aligned
```

[600]: 0.6903185830276566

[601]:
```python
chr1, chr2 = match_seq(x45[0]['E'], lxt[0]['F'])
print(f'{len(chr1)} atoms aligned')
superimpose_atoms(chr1, chr2)
```

```
target             0 QNVLYENQKLIANQFNSAIGKIQDSLSSTASALGKLQDVVNQNAQALNTLVKQLSSNFGA
                   0 ------------------------------------------------------------
query              0 ------------------------------------------------------------

target            60 ISSVLNDILSRLDKVELGDISGINASVVNIQKEIDRLNEVAKNLNESLIDLQE 113
                  60 ------------------|||.||||||||..||..|.||||.|.|||||||- 113
query              0 ------------------DISQINASVVNIEYEIKKLEEVAKKLEESLIDLQ-  34

34 atoms aligned
```

[601]: 1.0270533745363397

2.7 Sanity check, verify RMSD calculation with 4 atom example

[578]:
```python
m1_ = np.array([np.array([p.x,p.y,p.z]) for p in m1[:4]])
m2_ = np.array([np.array([p.x,p.y,p.z]) for p in m2[:4]])
```

[579]:
```
s = gemmi.superpose_positions(m2[:4],m1[:4])
print(s.rmsd)
```

```
0.5009230747740723
```

[580]:
```
m1_new = np.array(s.transform.mat)@(m1_.T) + np.array(s.transform.vec.tolist()).
↪T[:,np.newaxis]
#m2_new = (np.array(s.transform.mat)@((m2_ + np.array(s.transform.vec.
↪tolist())).T)).T
```

[581]:
```
print(m1_new)
```

```
[[-22.61314338 -23.59787179 -26.38141146 -23.71457336]
 [ 21.69045804  19.13483965  21.45595698  23.55274532]
 [ 14.76544482  12.11262489  10.93004837   9.17788193]]
```

[582]:
```
print(m2_.T)
```

```
[[-22.547 -23.412 -26.468 -23.88 ]
 [ 21.314  19.489  21.602  23.429]
 [ 15.367  12.061  10.89    8.668]]
```

[583]:
```
d = m1_new - m2_.T
print(np.sqrt(d**2))
print(d.shape)
np.sqrt(np.sum((d**2))/4)
```

```
[[0.06614338 0.18587179 0.08658854 0.16542664]
 [0.37645804 0.35416035 0.14604302 0.12374532]
 [0.60155518 0.05162489 0.04004837 0.50988193]]
(3, 4)
```

[583]: 0.5009230747740747
