# Supplementary material for: Enhancing the solubility of SARS-CoV-2 inhibitors to increase future prospects for clinical development
Source: J Virol. 2025 Feb 4;99(3):e02159-24. doi: 10.1128/jvi.02159-24 (PMC11915835; doi:10.1128/jvi.02159-24)
Supplement: Supplemental material — Supplemental methods, Fig. S1 to S29, and Table S1. [file jvi.02159-24-s0002.docx]

**Supporting Information**

**Enhancing the solubility of lipopeptide SARS inhibitors to increase clinical potential**

**Ariel J. Kuhn***^1^**, Victor K. Outlaw***^1^**, Tara C. Marcink***^2,3^**, Zhen Yu**^1^**, Megan C. Mears**^4,5^, **Maria N. Cajimat**^4^**, Dale F. Kreitler**^6^**, Payton R. Cleven**^1^**, Jee Ching Mook**^1^**, Dennis A. Bente**^4,7^**, Matteo Porotto**^2,3,8^**, Samuel H. Gellman**^1^**, Anne Moscona**^2,3,9,10^

^1^Department of Chemistry, University of Wisconsin, Madison, WI, USA

^2^Center for Host–Pathogen Interaction, Columbia University Medical Center, New York, NY, USA

^3^Department of Pediatrics, Columbia University Medical Center, New York, NY, USA

^4^Galveston National Laboratory, University of Texas Medical Branch, Galveston, TX, USA

^5^U.S. Department of Agriculture, Athens, GA

^6^Center for BioMolecular Structure, NSLS-LL, Brookhaven National Laboratory, Upton, NY, USA

^7^Department of Experimental Pathology, University of Texas Medical Branch, Galveston, TX, USA,

^8^Department of Experimental Medicine, University of Campania “Luigi Vanvitelli”, 81100 Caserta, Italy

^9^Department of Microbiology & Immunology, Columbia University Medical Center, New York, NY, USA

^10^Department of Physiology & Cellular Biophysics, Columbia University Medical Center, New York, NY, USA

**Table of Contents**

Peptide synthesis and purification………………………………………………………………………3

Instrumentation………………………………………………………………………………..…3

General procedures…………………………………………………………………….……..…3

Synthesis of lipopeptides……………………………………………………………………..…4

Solubility……………………………………………………………………………………………..…….5

Circular dichroism…………………………………………………………………………………………7

Cytotoxicity…………………………………………………………………………………….………..…8

Cell fusion assay of oxidized peptides……………………………………………………………….…9

Circular dichroism………………………………………………………………………………………10

Cell fusion assay of QE-A12………………………………………………………………..………….15

Sulfoxide formation scheme………………………………………………………………..…………..16

X-ray crystallography……………………………………………………………………………………18

Solubility UPLC Analysis………………………………………………………………………………..19

Peptide characterization…………………………………………………………….………………….21

References ………………………………………………………………………………………………39

**Peptide Synthesis and Purification**

**Instrumentation**

Solid-phase peptide synthesis was performed on a CEM Liberty Blue 1.0 microwave-assisted peptide synthesizer. Preparative HPLC was performed on an Agilent 1260 Infinity II Instrument. Peptide purity measurements were performed on a Waters Acquity H-Class UPLC. Mass spectra were obtained on a Bruker microflex LRF MALDI-TOF-MS. MSMS data were collected on a Bruker Impact II. Circular dichroism experiments were performed on a JASCO J-1500 CD spectrometer.

**General Procedures for Synthesis and Purification**

Peptides were prepared on Rink amide resin using microwave-assisted solid-phase peptide synthesis (MA-SPPS) procedures via a Liberty Blue 1.0. Resin was purchased from Millipore–Sigma. Fmoc-amino acids and coupling reagents were purchased from Chem-Impex International. Protected Fmoc-α-amino acids included: Asp(t-Bu ester), Glu(t-Bu ester), His(trityl), Lys(Boc), Asn(trityl), Gln(trityl), Arg(Pbf), Ser(t-Bu ether), Thr(t-Bu ether), Trp(Boc), and Tyr(t-Bu ether). Rink amide resin was pre-swelled with 1:1 DCM:DMF. Coupling reactions were performed using solutions comprised of 4 equivalents Fmoc-amino acid, 4 equivalents of OxymaPure and 8 equivalents of DIC in biotechnology-grade dimethylformamide (DMF) at a final concentration of 200 mM Fmoc-amino acid. Coupling reactions were carried out by microwave-assisted synthesis using a 2 min hold at 90 ºC. Deprotection was conducted by addition of 20% (v/v) piperidine in biotechnology-grade DMF. The deprotection reactions were carried out by microwave-assisted synthesis using a 2 min ramp to 80 ºC followed by a 2 min hold at 80 ºC. Each peptide was cleaved with 8.5:1:0.25:0.25 TFA/TIPS/ethanedithiol/phenol and precipitated by addition of cold diethyl ether

Preparative HPLC was performed using an Agilent 1250 Infinity II Prep HPLC equipped with a Waters XSelect CSH Prep C18 column (5 µm OBD, 19x250mm). Peptide purity measurements were performed on a Waters Acquity H-Class UPLC equipped with equipped with an Acquity UPLC CSH C18 (130Å, 1.7 µm, 2.1 x 100 mm) or an Acquity UPLC BEH C4 (300Å, 1.7 µm, 2.1 x 100 mm) column. Mass spectra were obtained on a Bruker microflex LRF-MALDI-TOF-MS. MS-MS data were collected on a Bruker Impact II. MSMS data were analyzed using Bruker Compass DataAnalysis 4.3 and GPMAW 10.

**Synthesis of Lipopeptides**

The cholesterol conjugation was achieved using displacement of an α-bromoamide. In a nitrogen-purged vial, BrAcNH-PEG4-Chol reagent (1.5 equiv.) was dissolved in degassed DMSO. In a separate nitrogen-purged vial outfitted with a stir bar, Ac-peptide-GSGSGC-NH2 (1.0 equiv.) was dissolved in degassed DMSO. The BrAcNH-PEG4-Chol solution was added to the peptide solution dropwise via syringe, followed by addition of N,N-diisopropylethylamine (DIEA). The reaction mixture was stirred for 90 minutes. Tris(2-carboxyethyl)phosphine (2.0 equiv.) was added, and the mixture was stirred for an additional 15 minutes. The resulting peptide-cholesterol conjugate was purified by reverse-phase HPLC on an Agilent Prep HPLC using a gradient of 55-75% acetonitrile+0.1% trifluoroacetic acid in water+0.1% trifluoroacetic acid over 30 minutes at a flow rate of 18 mL/min.


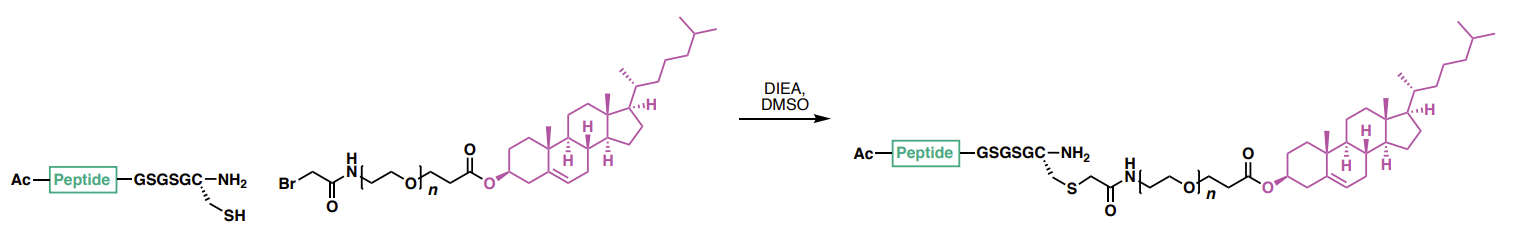


**
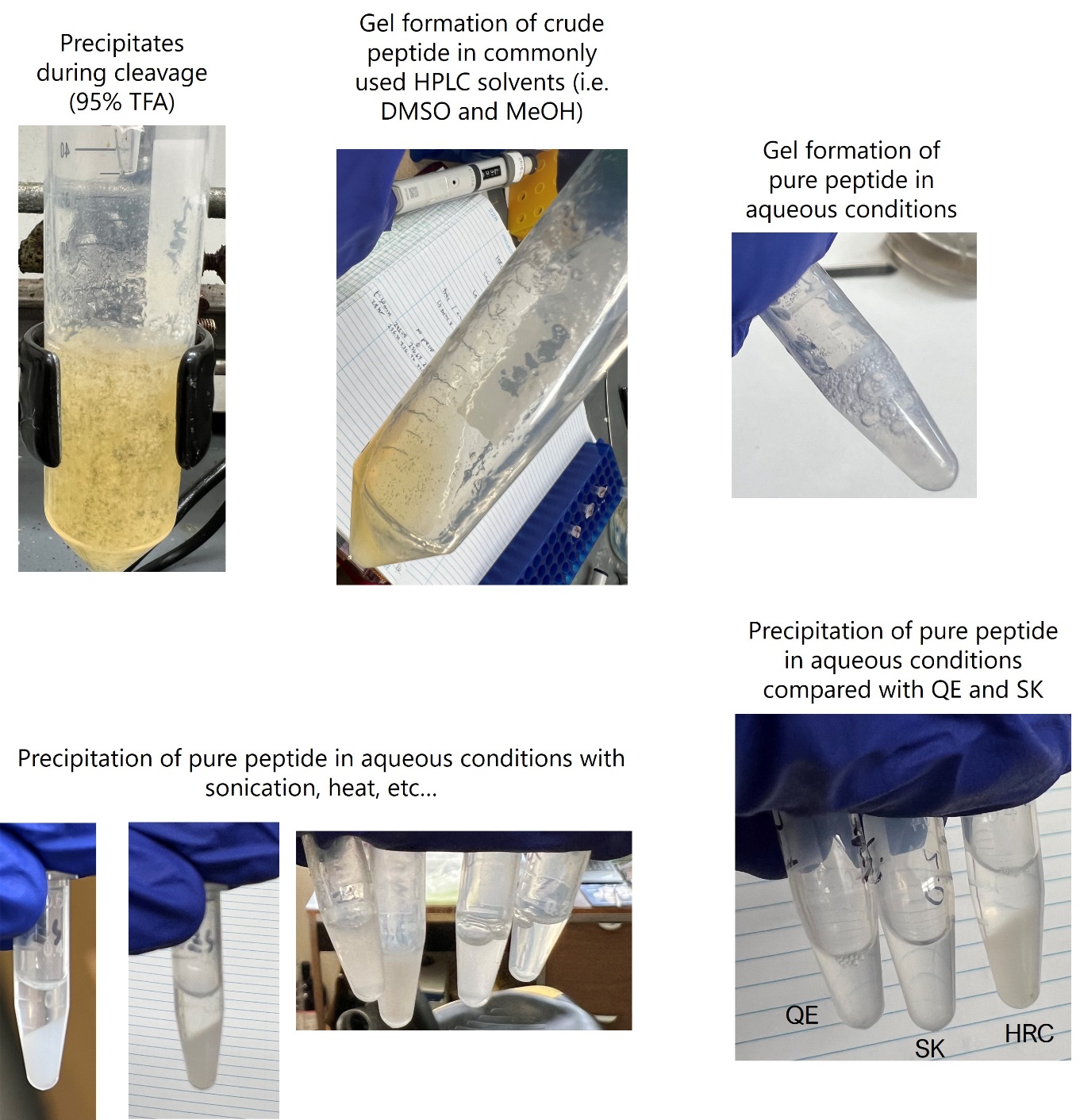
**

**Supplemental Figure 1.** **Technical issues encountered during synthesis, purification, and analysis of HRC and HRC-L peptides.** During cleavage from solid support in 95% trifluoracetic acid (TFA), HRC precipitates out of solution. During dissolution of crude peptide in common HPLC solvents, such as DMSO, MeOH, and acetonitrile, HRC undergoes irreversible gel formation or precipitation. Once pure, HRC precipitates and/or gels in several experimental aqueous conditions, such as when sonicated, agitated, or slightly heated.

**
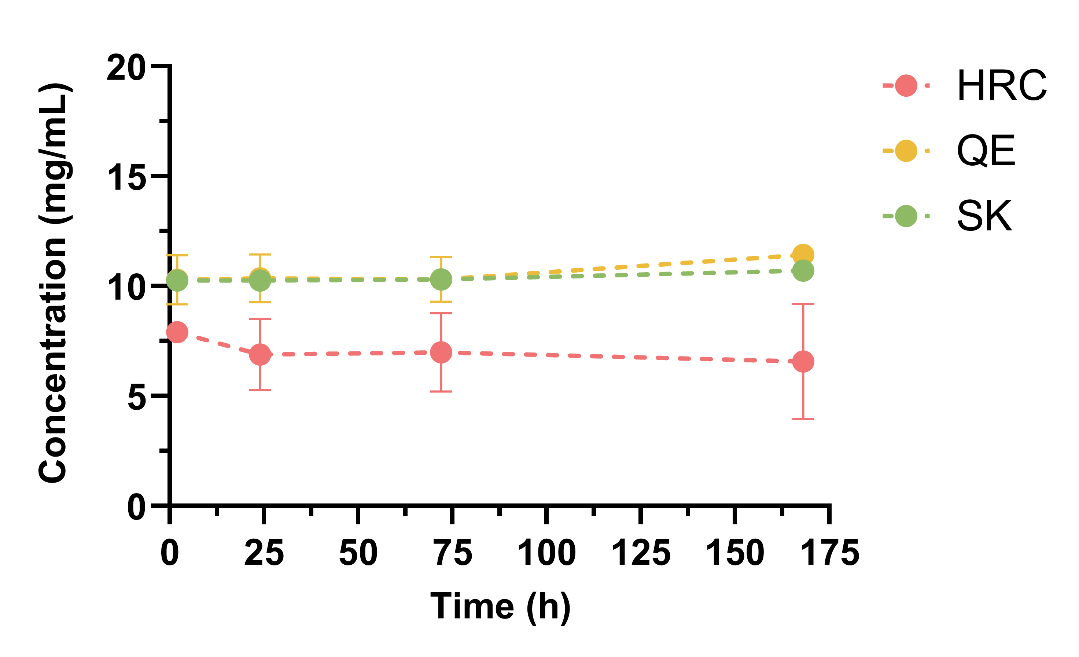
**

**Supplemental Figure 2.** **Solubility assessment of peptides.** Solubility of HRC, QE, and SK peptides in 50 mM phosphate buffer (pH 7.4) as measured by UV absorbance at 280 (QE and SK) and 205 nm (HRC) over time. ε_205_(HRC-L) = 118,820 M^-1^ cm^-1^ and ε_205_(HRC) = 101,450 M^-1^ cm^-1^.

**EK1:** Ac- S L D Q I N V T F L D L E Y E M K K L E E A I K K L E E S Y I D L K E L - NH_2_


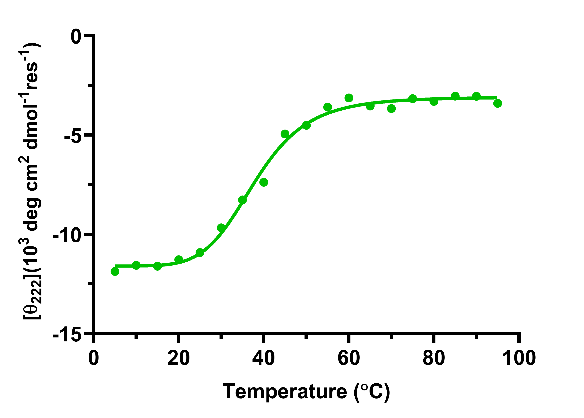


**Supplemental Figure 3.** **Circular dichroism studies of SARS-CoV-2 tHRN + EK1 co-assembly.** Temperature-dependent denaturation of co-assembly formed between a 1:1 mixture of tHRN and EK1. 10 µM total peptide concentration in 10 mM phosphate buffer, pH 7.4.

T_m_ = 38 ± 0.3 °C


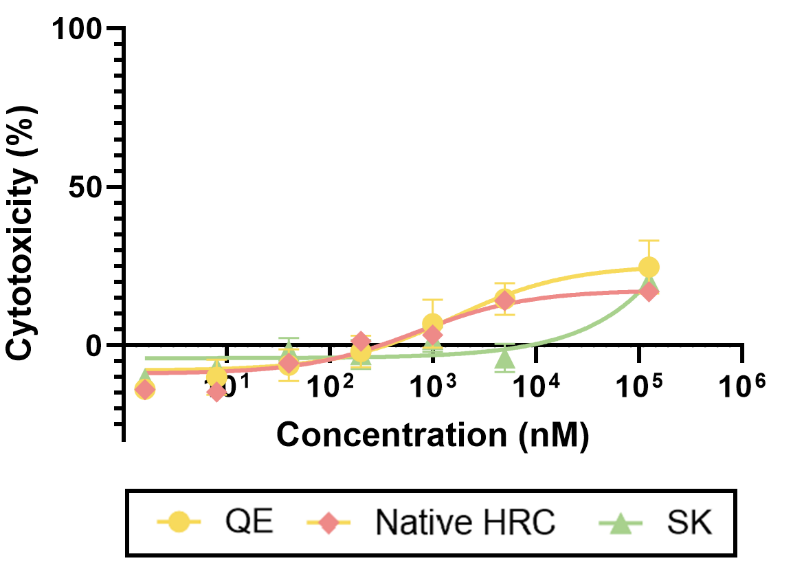


**Supplemental Figure 4: Peptide toxicity.** To determine the toxicity of the new lipopeptides, a 3-(4,5-dimethylthiazol-2-yl)-2,5-diphenyltetrazolium bromide (MTT) assay was performed in parallel with the fusion inhibition studies. Toxicity of the lipopeptides was minimal for each lipopeptide tested (<20% at 100 nM). No toxicity was observed for any of the lipopeptides at the respective IC90s. For **SK**, no toxicity was observed up to 10 µM peptide concentration, while all other lipopeptides exhibited ~20% toxicity at this concentration.


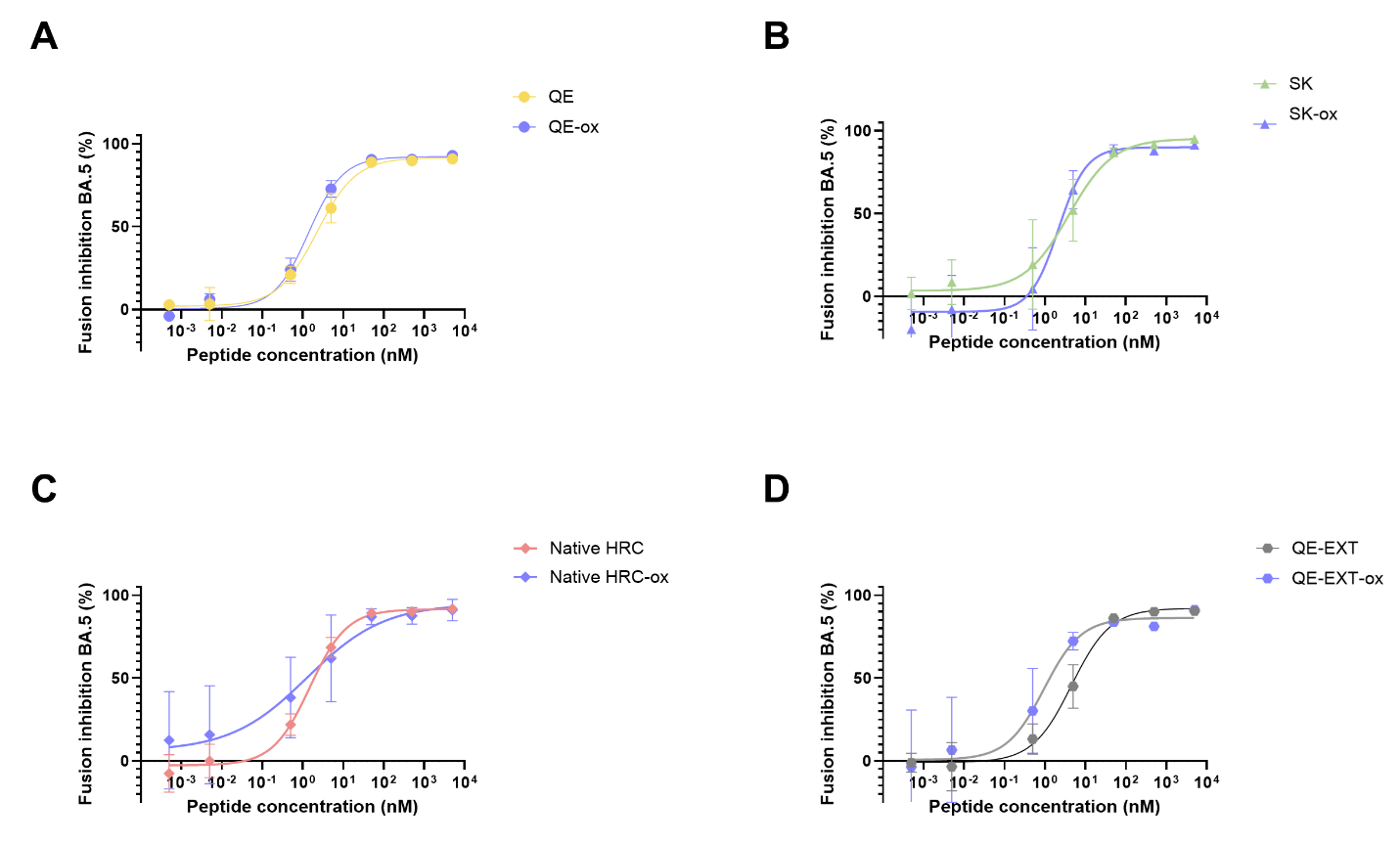


**Supplemental Figure 5: Inhibition of SARS-CoV-2 spike (S)-mediated cell-cell fusion with oxidized peptides.** Fusion inhibitory activity of oxidized (-ox) and non-oxidized peptides against SARS-CoV-2 S variant BA.5. The non-oxidized peptides have a thioether linkage at the C-terminal Cys side chain, while the oxidized peptides contain a sulfoxide. The percent inhibition is depicted for QE (**A**), SK (**B**), Native HRC (**C**), and QE-ext (**D**) at increasing concentrations. The percent fusion inhibition was calculated as the ratio of luminescence in the presence of peptide at a specific concentration (X) to the luminescence in the absence of inhibitor. Percent inhibition = 100 x [1 - (luminescence at X)/(luminescence in absence of peptide)]. Data in (A) and (B) are mean ± standard error of the mean (SEM) from three distinct experiments with a four-parameter variable curve.


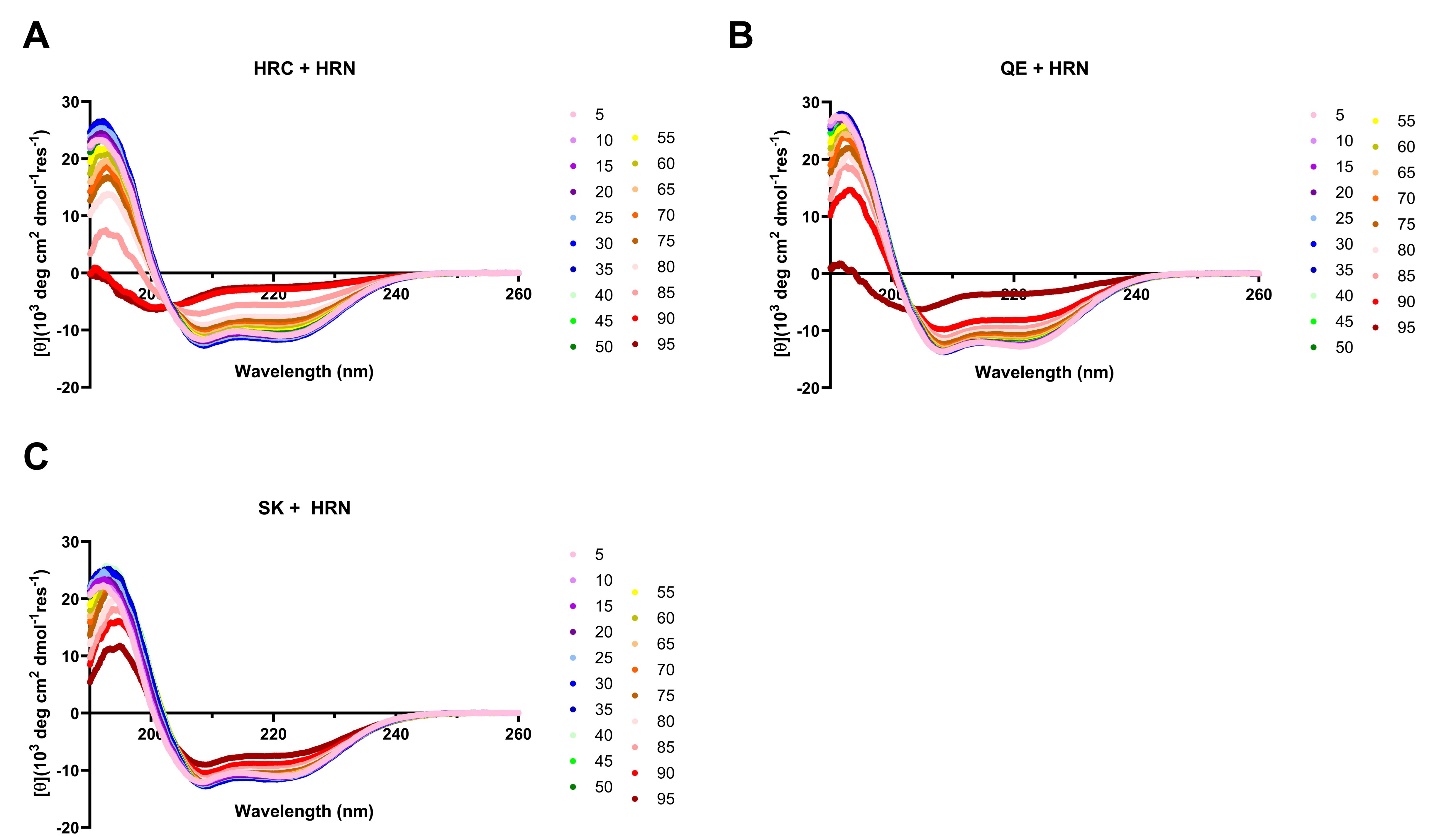


**Supplemental Figure 6.** **Circular dichroism studies of SARS-CoV-2 HRN + HRC hybrid co-assemblies.** Full CD scans from temperature-dependent denaturation of co-assemblies formed by 1:1 mixtures of HRN with (A) **HRC**, (B) **QE** or (C) **SK**. 50 µM total peptide concentration in 10 mM phosphate buffer, pH 7.4. Legends indicate temperature in °C.


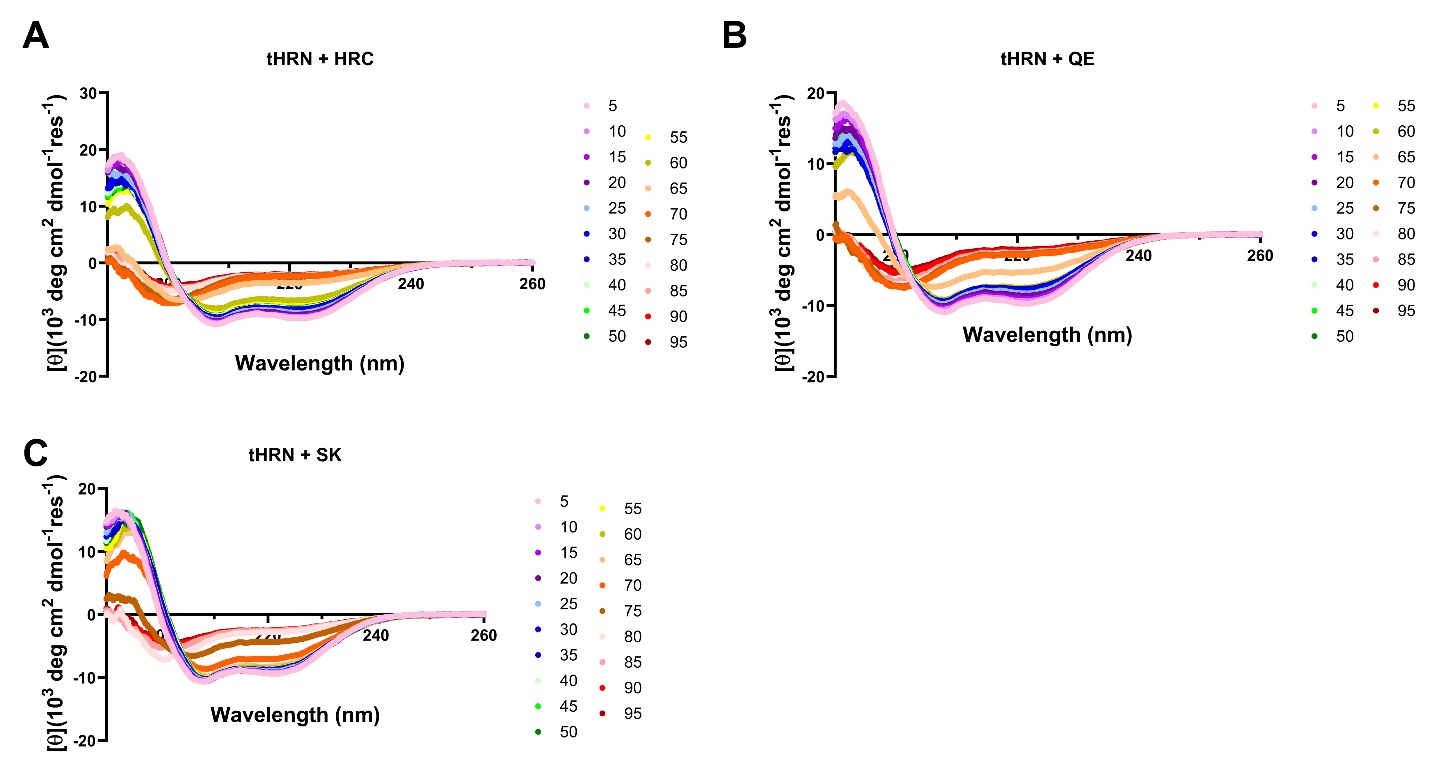


**Supplemental Figure 7.** **Circular dichroism studies of SARS-CoV-2 tHRN + HRC hybrid co-assemblies.** Full CD scans from temperature-dependent denaturation of co-assemblies formed in 1:1 mixtures of tHRN with (A) **HRC**, (B) **QE** or (C) **SK**. 50 µM total peptide concentration in 10 mM phosphate buffer, pH 7.4. Legends indicate temperature in °C.

**QE:** Ac –D I S Q I N A S V V N I E Y E I K K L E E V A K K L E E S L I D L Q E L - NH_2_

**QE-A12:** Ac –D I S Q I N A S V V N **A** E Y E I K K L E E V A K K L E E S L I D L Q E L - NH_2_


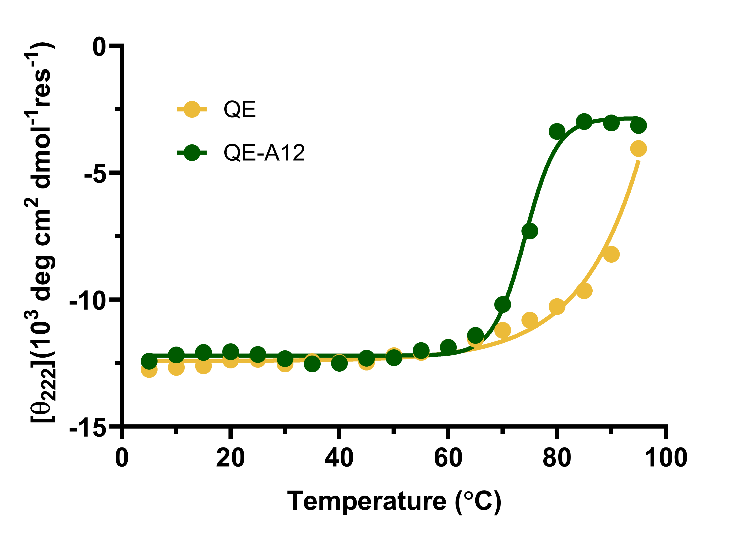


**Supplemental Figure 8. Circular dichroism studies of SARS-CoV-2 HRN + the Ile12-->Ala variant of the native HRC.** Temperature-dependent denaturation of co-assemblies formed in 1:1 mixtures of HRN with **QE** or **QE**-A12. **QE**-A12 T_m_ = 74 °C. 50 µM total peptide concentration in 10 mM phosphate buffer, pH 7.4.

**QE:** Ac –D I S Q I N A S V V N I E Y E I K K L E E V A K K L E E S L I D L Q E L - NH_2_

**QE-25 (fragment of QE):** Ac – I E Y E I K K L E E V A K K L E E S L I D L Q E L - NH_2_


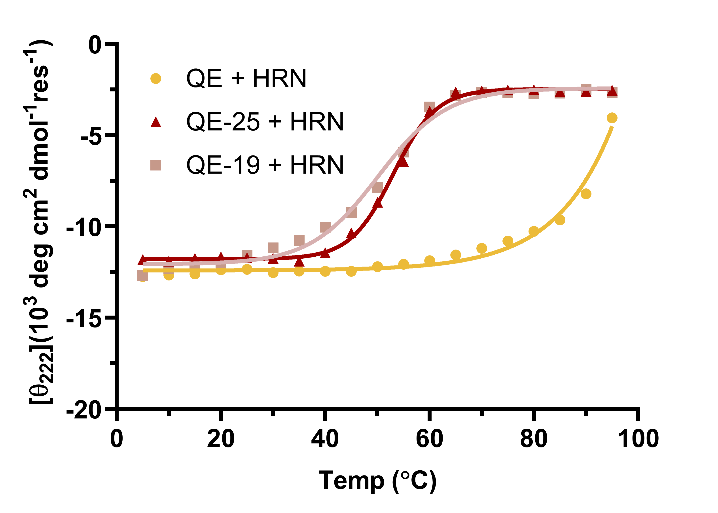
**QE-19 (fragment of QE):** Ac – I E Y E I K K L E E V A K K L E E S L - NH_2_

**Supplemental Figure 9.** **Circular dichroism studies of SARS-CoV-2 HRN + truncated variants of QE.** Temperature-dependent denaturation of co-assemblies formed in 1:1 mixtures of HRN with **QE**, **QE**-25, or **QE**-19. **QE**-25 T_m_ = 53 °C; **QE**-19 T_m_ = 50 °C. 50 µM total peptide concentration in 10 mM phosphate buffer, pH 7.4.

**QE:** Ac –D I S Q I N A S V V N I E Y E I K K L E E V A K K L E E S L I D L Q E L - NH_2_

**QE1-EXT:**

Ac – P D V D L G - D I S Q I N A S V V N I E Y E I K K L E E V A K K L E E S L I D L Q E L - NH_2_


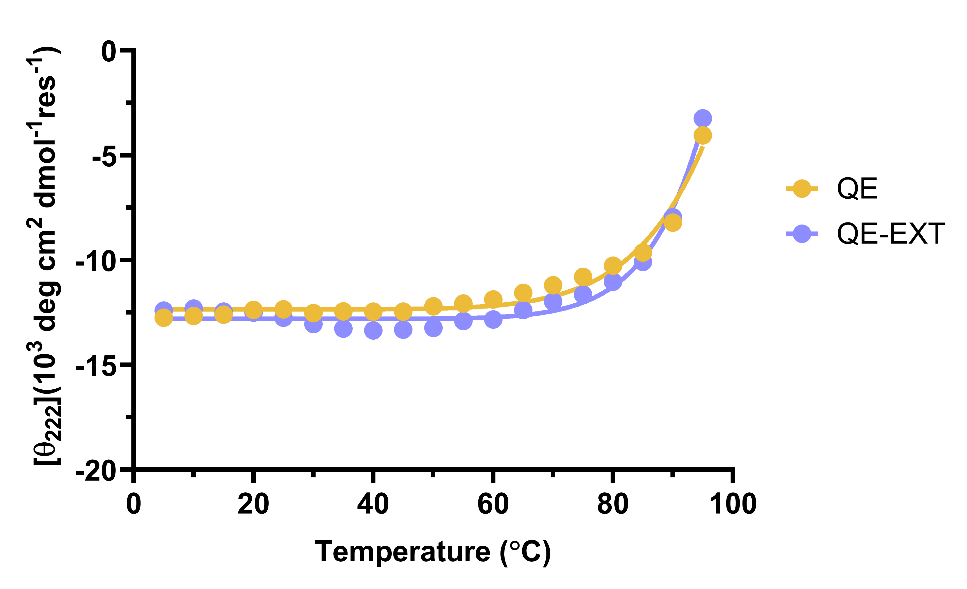


**Supplemental Figure 10.** **Circular dichroism studies of SARS-CoV-2 HRN + QE-EXT.** Temperature-dependent denaturation of co-assemblies formed in 1:1 mixtures of HRN with **QE** or **QE-EXT**. 50 µM total peptide concentration in 10 mM phosphate buffer, pH 7.4.

**HRN:**

Ac – T Q N V L Y E N Q K L I A N Q F N S A I G K I Q D S L S S T A S A L G K L Q D V V N Q N A Q A L N T L V K Q L - NH_2_

**tHRN:**

Ac –L I A N Q F N S A I G K I Q D S L S S T A S A L G K L Q D V V N Q N A Q A L N T L V K Q - NH_2_


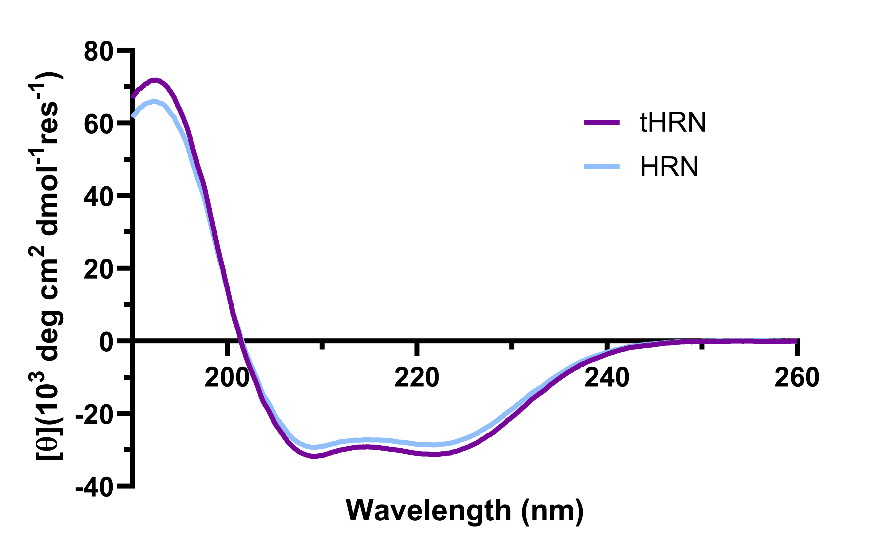


**Supplemental Figure 11.** **Circular dichroism studies of SARS-CoV-2 HRN peptides.** Full CD spectrum scans at 25 °C of HRN or tHRN. 25 µM total peptide concentration in 10 mM phosphate buffer, pH 7.4.

**Supplemental Figure 12.** Oxidation of cysteine thioether to the corresponding sulfoxide, which occurs spontaneously during chemical ligation of the cholesterol moiety. The sulfur atom of a sulfoxide is a stereogenic center; therefore, the sulfoxide form is presumably a mixture of two stereoisomers that differ in terms of the configuration at the sulfur center.

**X-ray Crystallography**

**Crystallization conditions**

Lyophilized powders of HRN and **QE** peptides were dissolved in an aqueous solution of 1% w/v b-D-octylglucoside to final respective concentrations of 4 mg/mL and 3 mg/mL. Peptide crystals were grown with hanging drop vapor diffusion, in which drops comprised of 1 uL peptide solution combined with 1 uL of well solution (0.1 M Tris pH 7.8, 26% w/v PEG3350, 0.3 M MgCl2) were equilibrated against 150 uL of well solution in a VDXm plate at 20°C. Crystals were cryo-protected by sequential soaking for 2-3 minutes each in cryo-solution A (0.1 M Tris pH 7.8, 37% w/v PEG3350, 0.3 M MgCl2) and cryo-solution B (0.1 M Tris pH 7.8, 48% w/v PEG3350, 0.3 M MgCl2) followed by vitrification in liquid nitrogen.

**X-ray Data Collection**

X-ray diffraction data were collected from a single crystal at the AMX beamline (17-ID-1) at NSLS-II at Brookhaven National Laboratory, Upton, NY.

**X-ray Data Processing, Structure Solution, and Refinement**

Diffraction data were indexed and integrated with *XDS* (1), then scaled and merged with *XDS*/*aimless* as implemented in the *autoPROC* software package (2). Initial phases were obtained with molecular replacement via *phenix.phaser* using the corresponding residues in the HRN/HRC hairpin dimer from 6LXT (3-4). Refinement of XYZ, ADP, and TLS parameters was performed with *phenix.refine* in space group *P*2_1_2_1_2 combined with iterative real space refinement in Coot (3,5).

**Supplemental Table 1. SARS-CoV-2 HRN + SARS2-QE**

| PDB ID | 6X45 |
| --- | --- |
| Wavelength (Å) | 0.920087 |
| Resolution Range (Å) | 80.12-2.20 (2.27-2.20) |
| Space group | *P* 2_1_2_1_2 |
| a, b, c (Å) | 53.543 / 54.458 / 80.119 |
| α, β, γ (°) | 90 / 90 / 90 |
| R_merge_ | 0.082 (1.565) |
| R_meas_ | 0.096 (1.705) |
| R_pim_ | 0.038 (0.666) |
| Total number of observations | 79758 |
| Total number unique | 12351 |
| <I/sigI> | 9.0 (1.2) |
| CC_1/2_ | 0.999 (0.475) |
| Completeness (%) | 99.5 (99.2) |
| Multiplicity | 6.5 (6.3) |
| Wilson B value (Å^2^) | 54.79 |
| Refinement resolution (Å) | 45.04-2.20 (2.29-2.20) |
| Reflections used in refinement | 12313 |
| Reflections used for R-free | 1217 |
| R-work | 0.2430 (0.3284) |
| R-free | 0.2780 (0.4037) |
| No. Non-hydrogen atoms (macromolecule) | 1976 |
| No. Non-hydrogen atoms (solvent) | 8 |
| RMS (bonds) | 0.002 |
| RMS (angles) | 0.365 |
| Ramachandran favored (%) | 99.2 |
| Ramachandran outliers (%) | 0.0 |
| Rotamer outliers (%) | 0.5 |
| Mean B value (Å^2^) | 88.63 |
| No. TLS groups | 1 |
| *parentheses indicate statistics corresponding to highest resolution shell | |

**
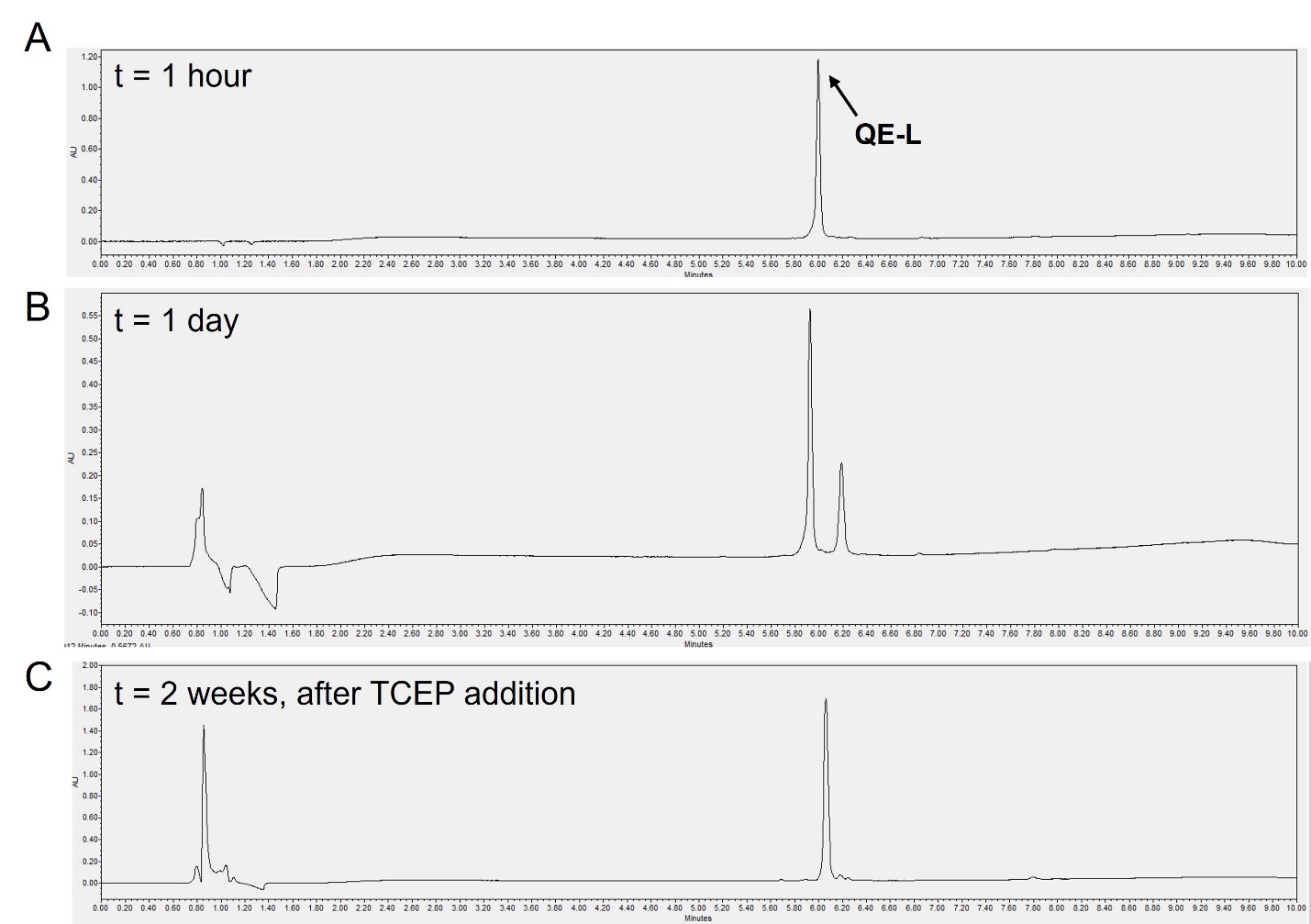
**

**Supplemental Figure 13.** **Solubility Studies.** UPLC analysis of **QE** solubility in PBS after equilibration times of (A) 1 hour, (B) 1 day, and (C) 2 weeks. After one day, a second peak appeared corresponding to disulfide formation between cysteine residues at the C-termini of **QE** (B). Tris(2-carboxyethyl)phosphine (TCEP) addition after 2 weeks eliminated the second peak, as a result of disulfide reduction (C). No peptide degradation was observed after 2 weeks of incubation in PBS at room temperature. UPLC gradient = 10-90% MeCN/H_2_O over 10 minutes (0.3 mL/min; column = Waters Acquity CSH C18, 130Å, 1.7 µm, 2.1 x 100 mm). Detection = 220 nm.

**
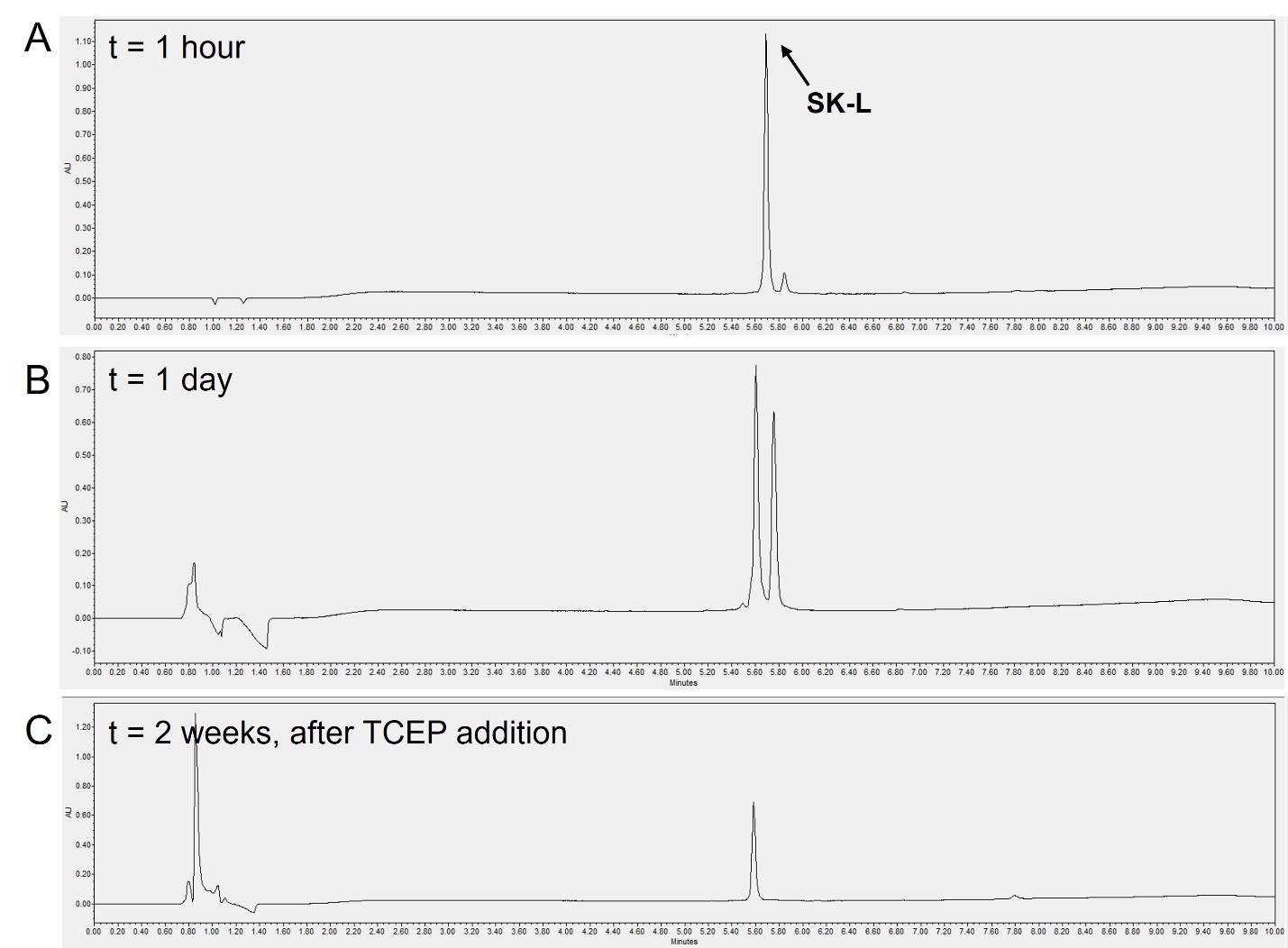
**

**Supplemental Figure 14.** **Solubility Studies.** UPLC analysis of **SK** solubility in PBS after equilibration times of (A) 1 hour, (B) 1 day, and (C) 2 weeks. After one day, a second peak appeared corresponding to disulfide formation between cysteine residues at the C-termini of **SK** (B). TCEP addition after 2 weeks eliminated the second peak, as a result of disulfide reduction (C). No peptide degradation was observed after 2 weeks of incubation in PBS at room temperature. UPLC gradient = 10-90% MeCN/H_2_O over 10 minutes (0.3 mL/min; column = Waters Acquity CSH C18, 130Å, 1.7 µm, 2.1 x 100 mm). Detection = 220 nm.

**Peptide Characterization Data**

**QE:** Ac –D I S Q I N A S V V N I E 0Y. E I K K L E E V A K K L E E S L I D L Q E L - NH_2_

Calculated monoisotopic [M+H]^+^: 4184.2; Observed [M+H]^+^: 4184.1


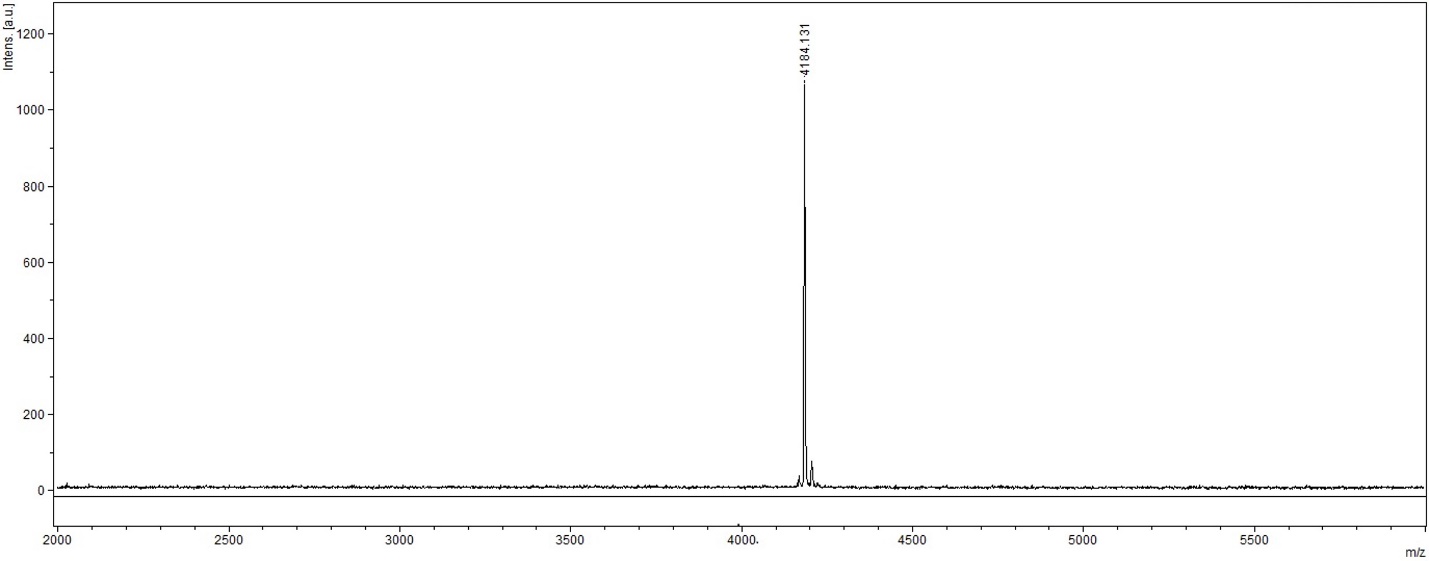


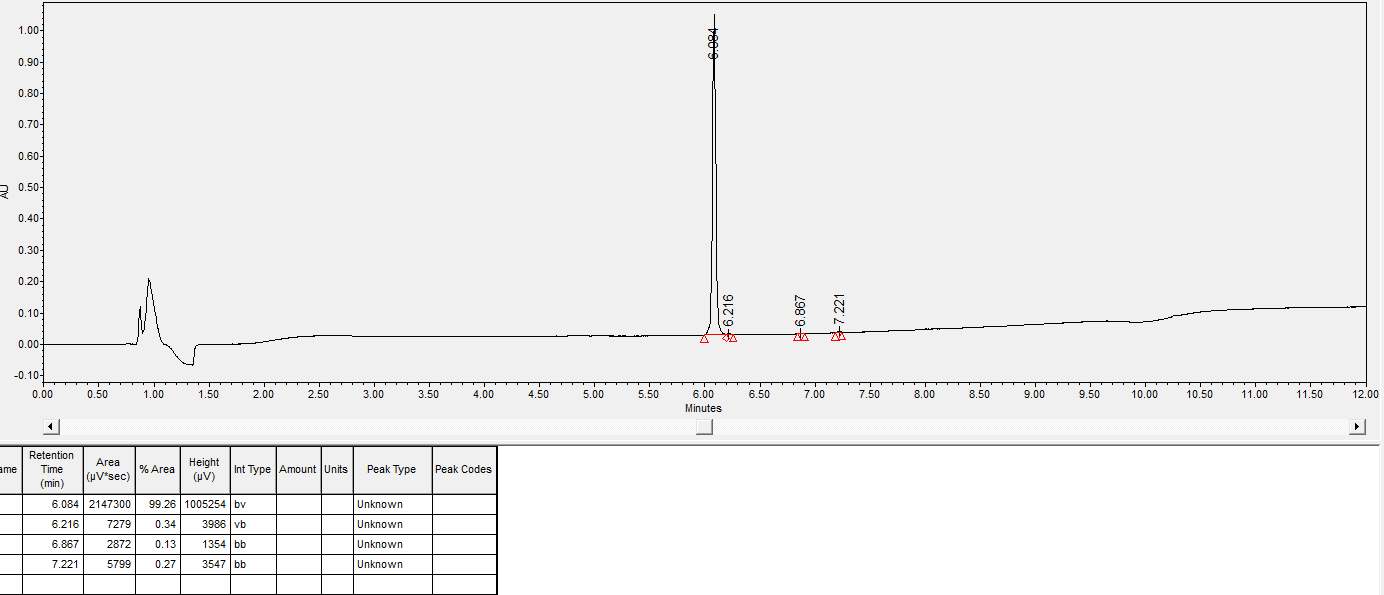


**Supplemental Figure 15.** MALDI-TOF and UPLC analysis of **QE**. Purity = 99.3%. UPLC gradient = 10-90% MeCN/H_2_O over 10 minutes (0.3 mL/min; column = Waters Acquity CSH C18, 130Å, 1.7 µm, 2.1 x 100 mm). Detection = 220 nm.

**Supplemental Figure 16.** **MSMS data for QE. (A)** MS and MSMS spectra of **QE. (B)** Fragmentation data for **QE.** Most b and y’ ions were observed during fragmentation, which strongly supports the existence of the proposed peptide sequence. Analysis was done in GPMAW 10.

**A)**

**
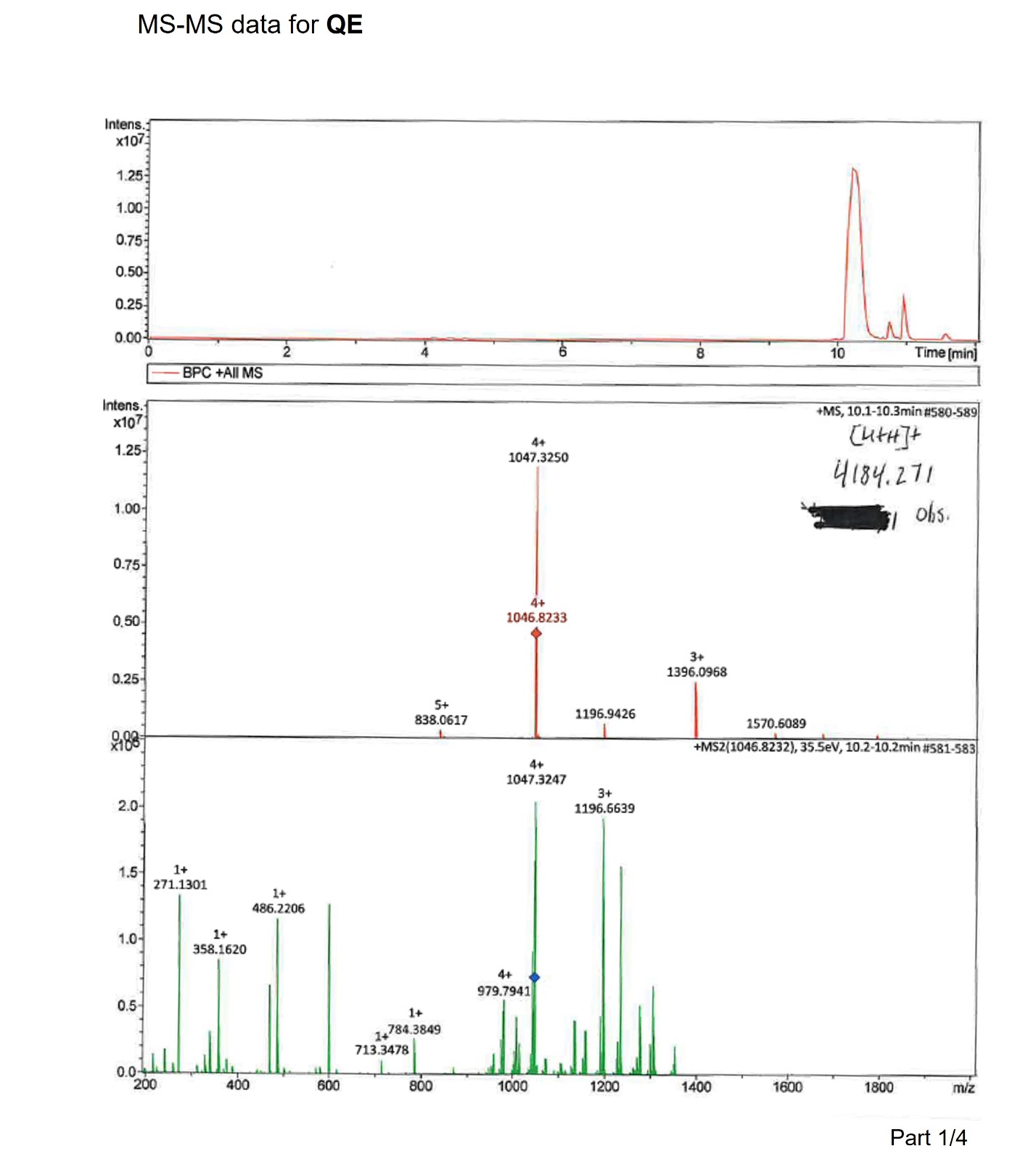
**

**B)**

**
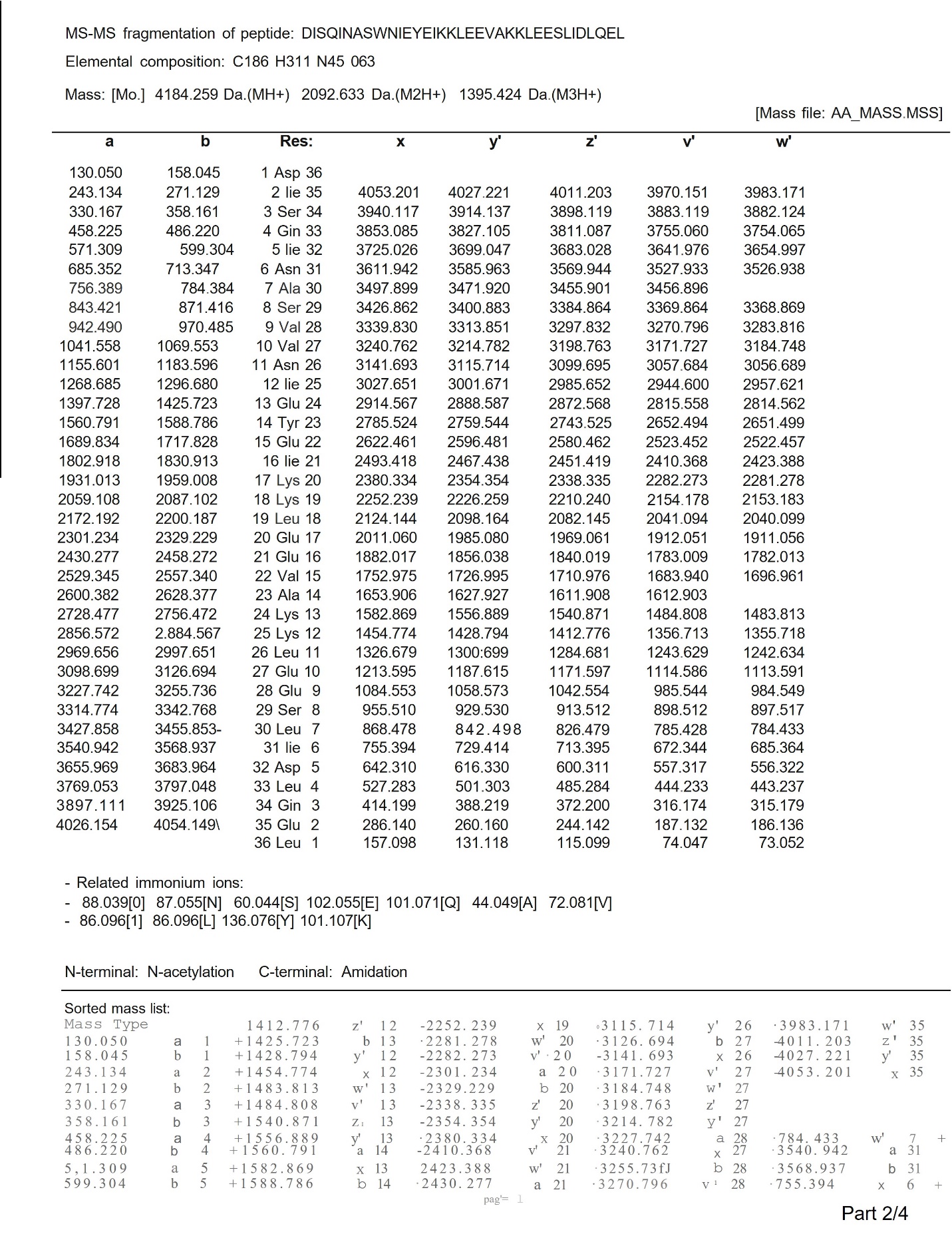
**

**QE-L:** Ac –D I S Q I N A S V V N I E Y E I K K L E E V A K K L E E S L I D L Q E L GSGSGC- NH_2_

Calculated monoisotopic [M+H]^+^: 4632.4; Observed [M+H]^+^: 4632.9


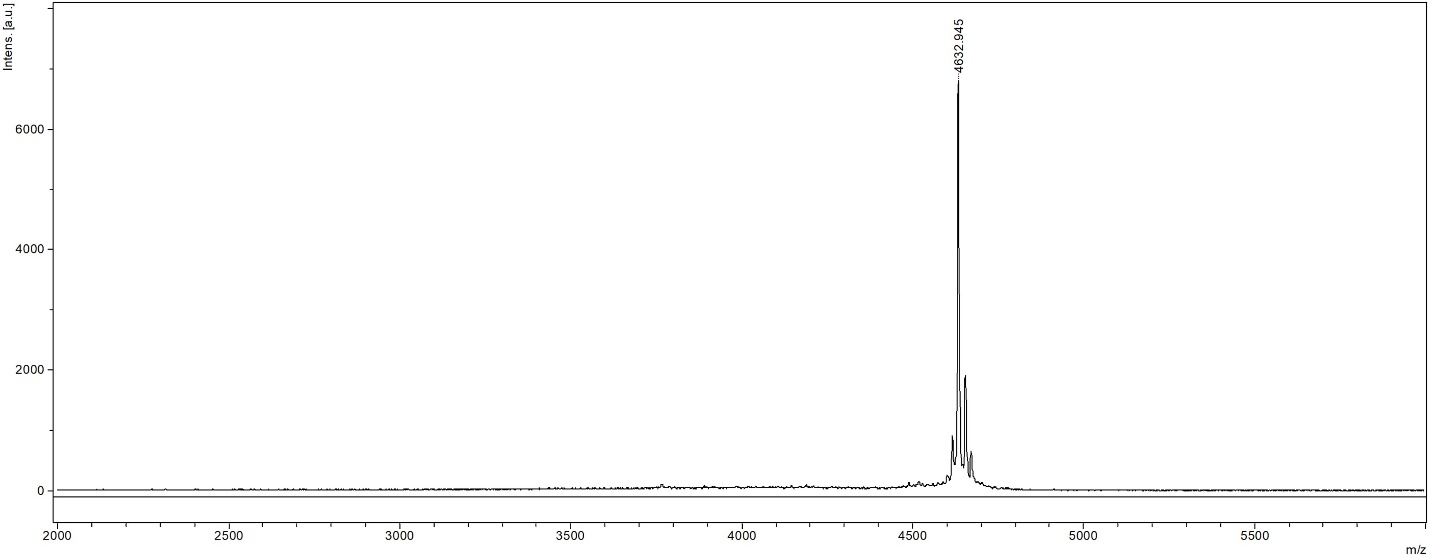


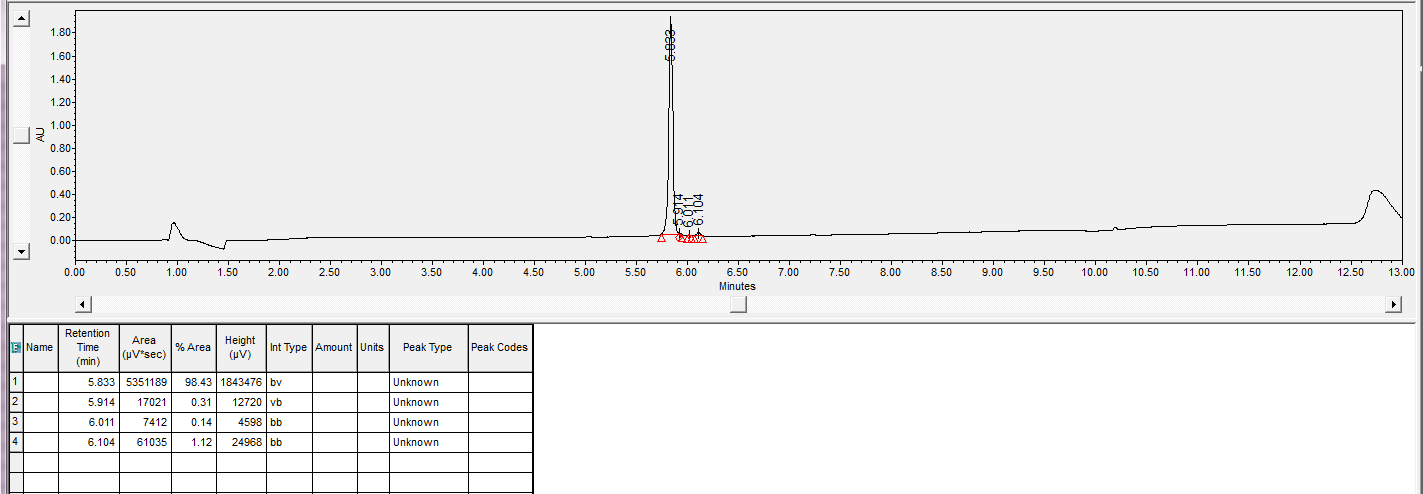


**Supplemental Figure 17.** MALDI-TOF and UPLC analysis of **QE-L**. Purity = 98.4%. UPLC gradient = 10-90% MeCN/H_2_O over 10 minutes (0.3 mL/min; column = Waters Acquity CSH C18, 130Å, 1.7 µm, 2.1 x 100 mm). Detection = 220 nm.

**SK:** Ac-SIDQINATFVDIEYEIKKLEEVAKKLEESYIDLKEL-NH_2_

Calculated monoisotopic [M+H]^+^: 4297.3; Observed [M+H]^+^: 4297.5


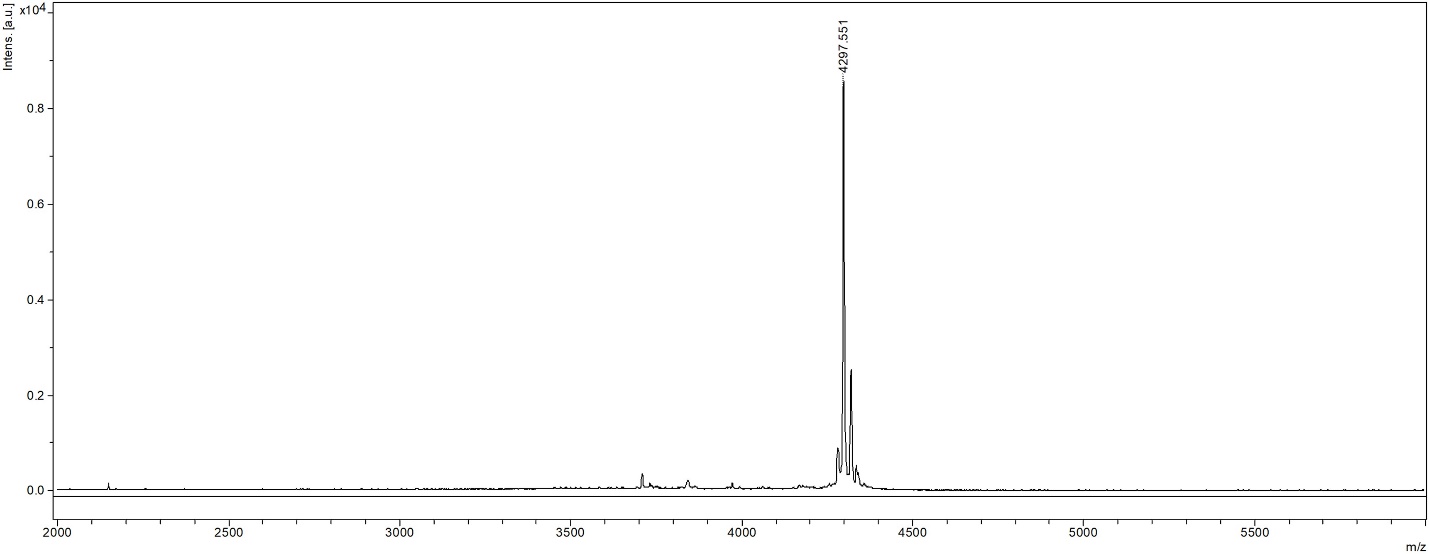


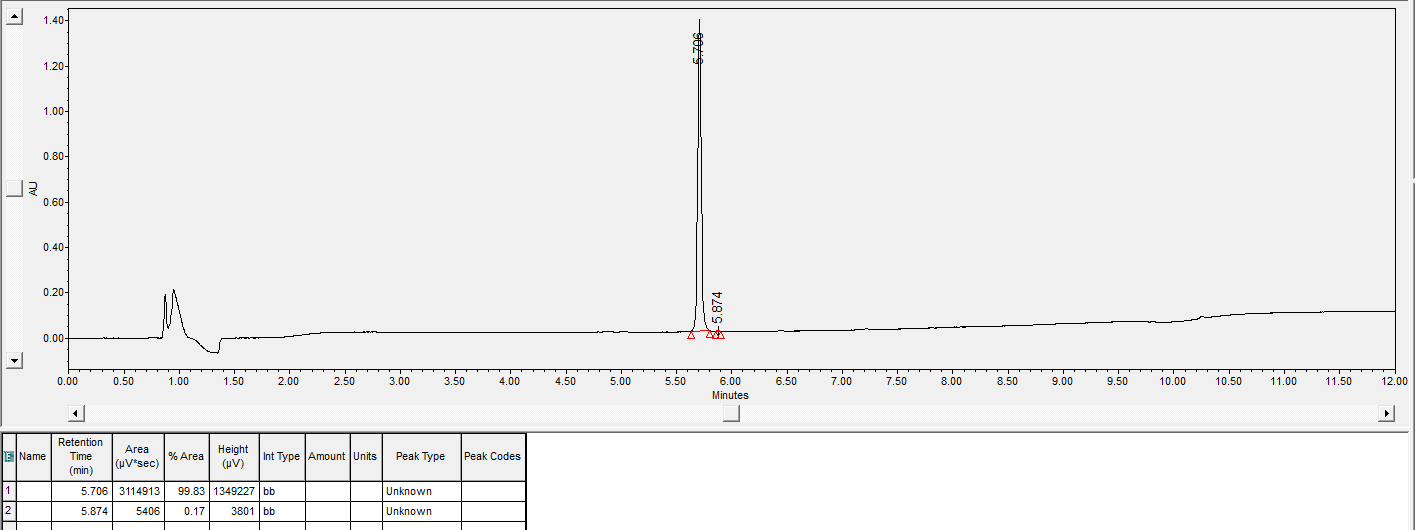


**Supplemental Figure 18.** MALDI-TOF and UPLC analysis of **SK**. Purity = 99.8%. UPLC gradient = 10-90% MeCN/H_2_O over 10 minutes (0.3 mL/min; column = Waters Acquity CSH C18, 130Å, 1.7 µm, 2.1 x 100 mm). Detection = 220 nm.

**Supplemental Figure 19.** **MSMS data for SK. (A)** MS and MSMS spectra of **SK. (B)** Fragmentation data for **SK.** Most b and y’ ions were observed during fragmentation, which strongly supports the existence of the proposed peptide sequence. Analysis was done in GPMAW 10.

**A)**

**
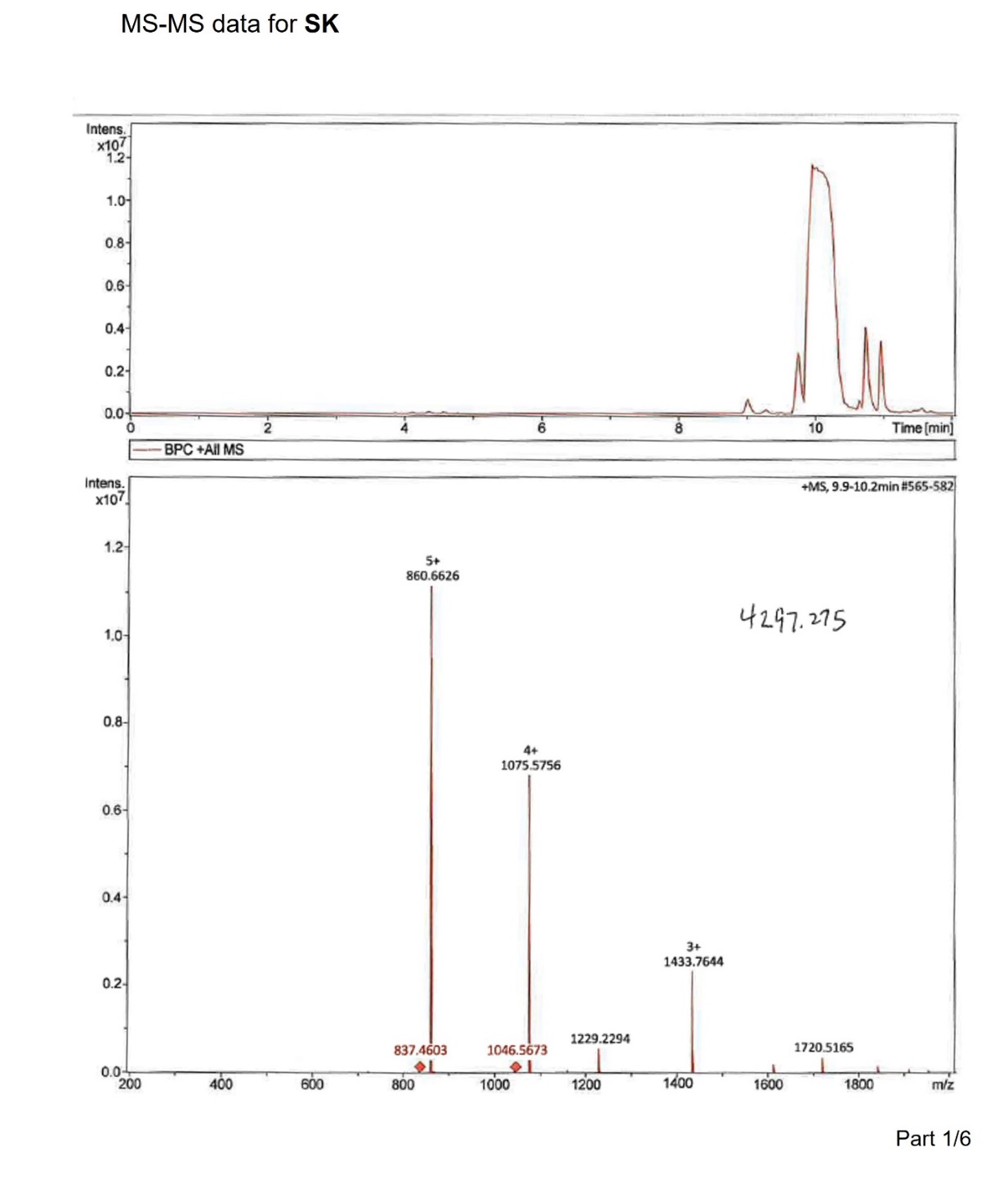
**

**
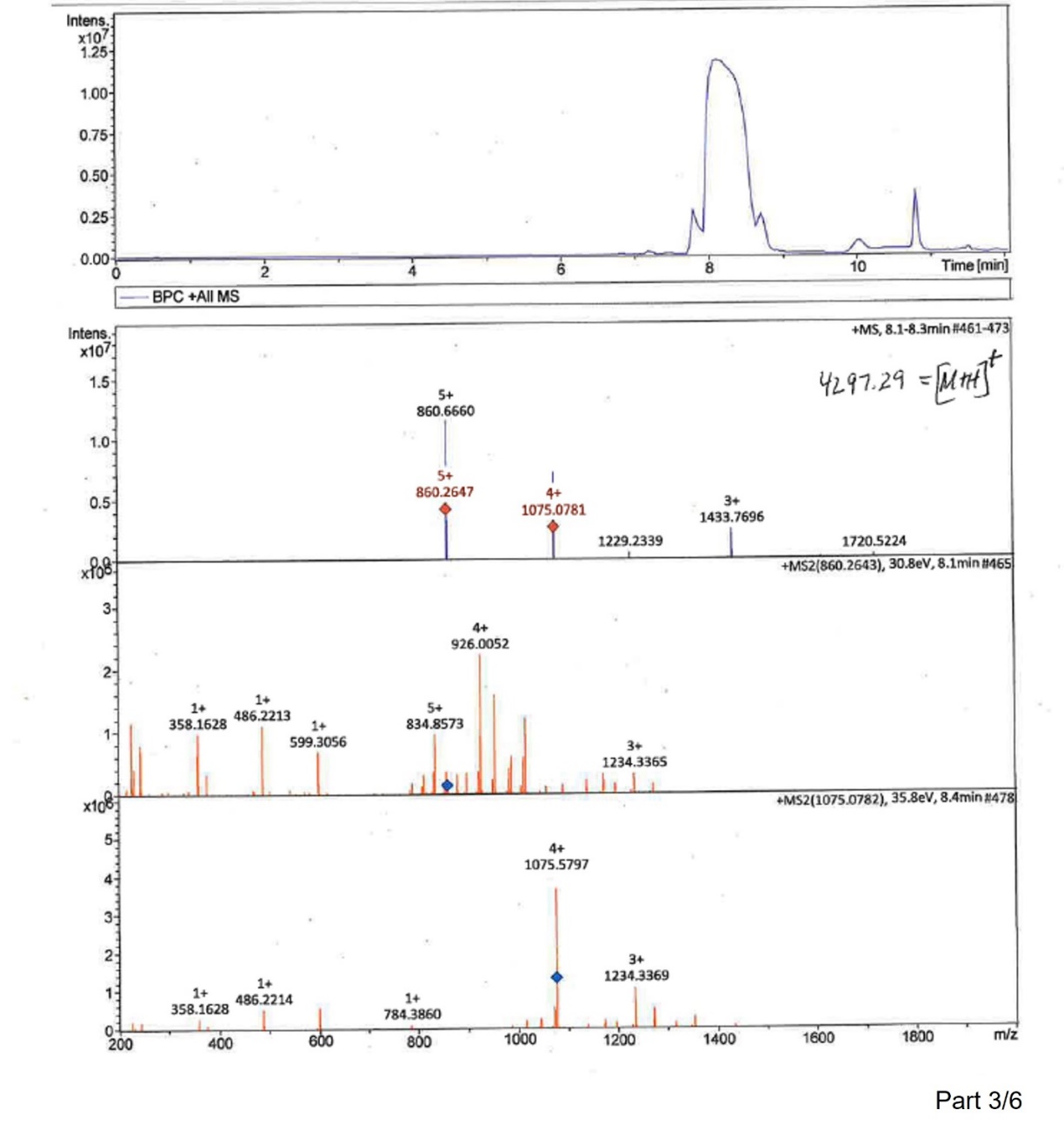
**

**
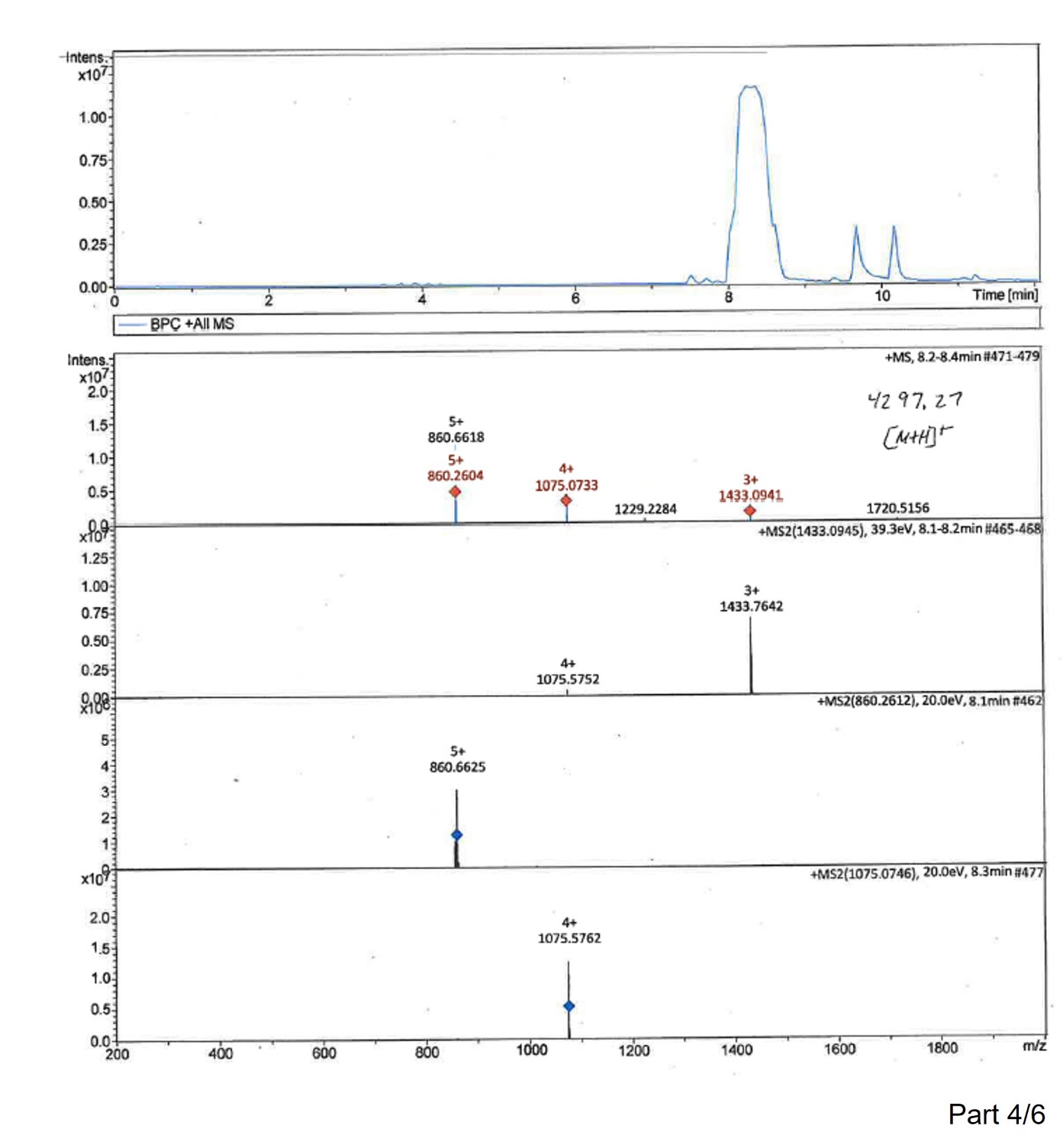
**

**B)**

**
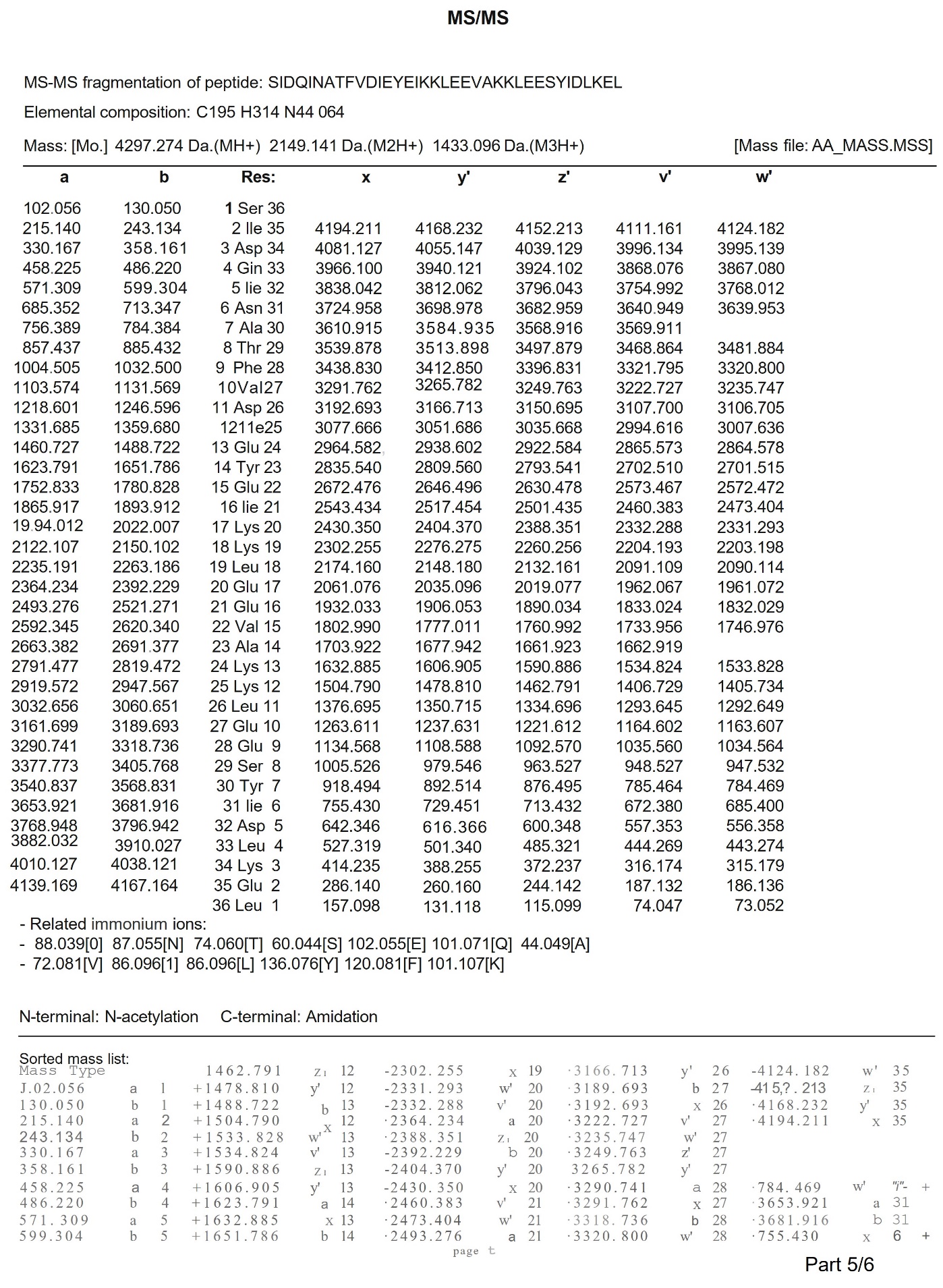
**

**SK-L:** Ac-SIDQINATFVDIEYEIKKLEEVAKKLEESYIDLKEL-GSGSGC-NH_2_

Calculated monoisotopic [M+H]^+^: 4745.4; Observed [M+H]^+^: 4745.4


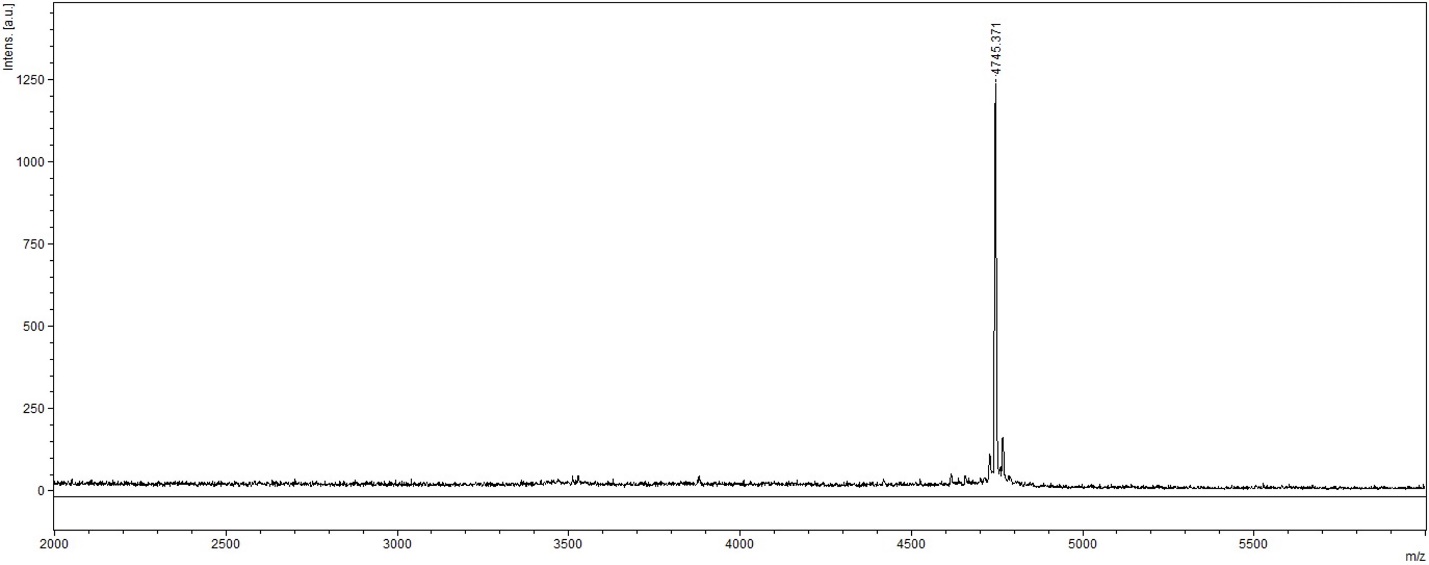


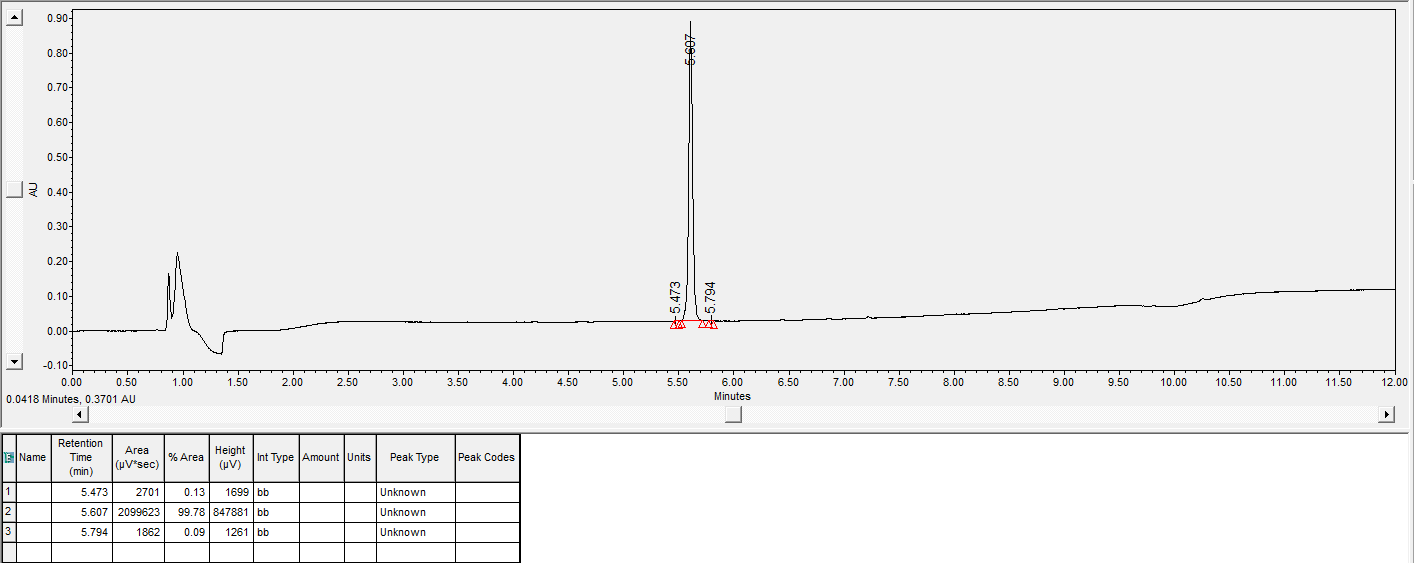


**Supplemental Figure 20.** MALDI-TOF and UPLC analysis of **SK-L**. Purity = 99.8%. UPLC gradient = 10-90% MeCN/H_2_O over 10 minutes (0.3 mL/min; column = Waters Acquity CSH C18, 130Å, 1.7 µm, 2.1 x 100 mm). Detection = 220 nm.

**HRC:** Ac- D I S G I N A S V V N I Q K E I D R L N E V A K N L N E S L I D L Q E L - NH_2_

Calculated monoisotopic [M+H]^+^: 4048.1; Observed [M+H]^+^: 4049.6


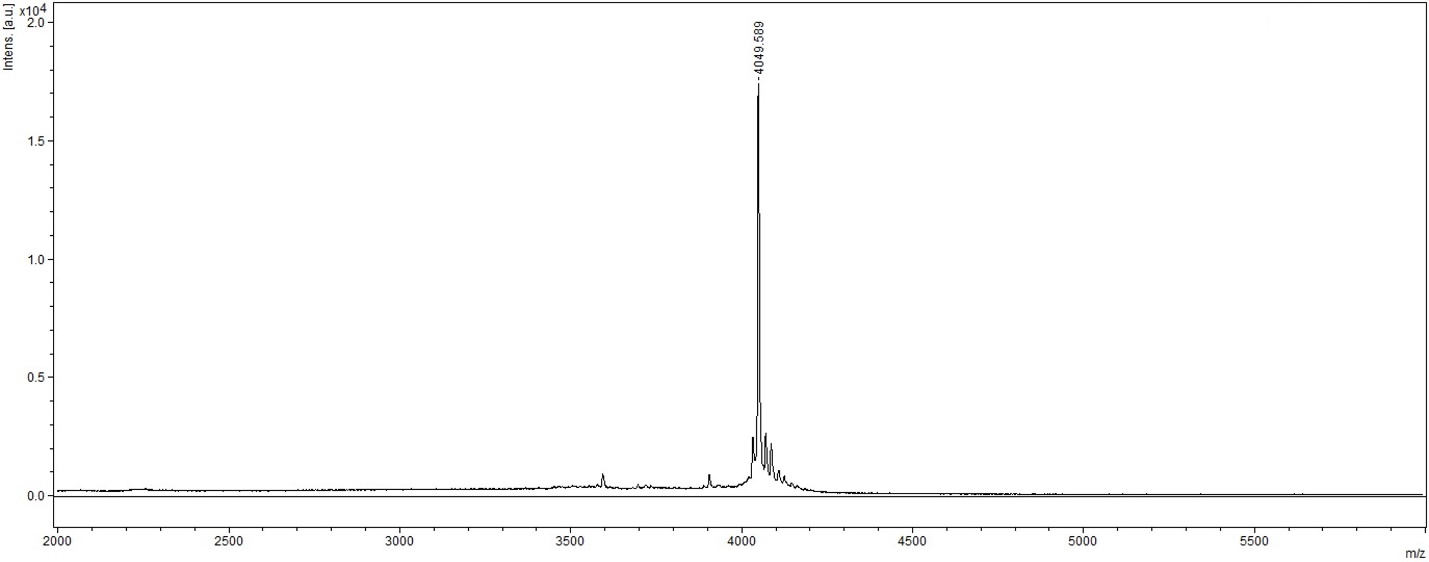


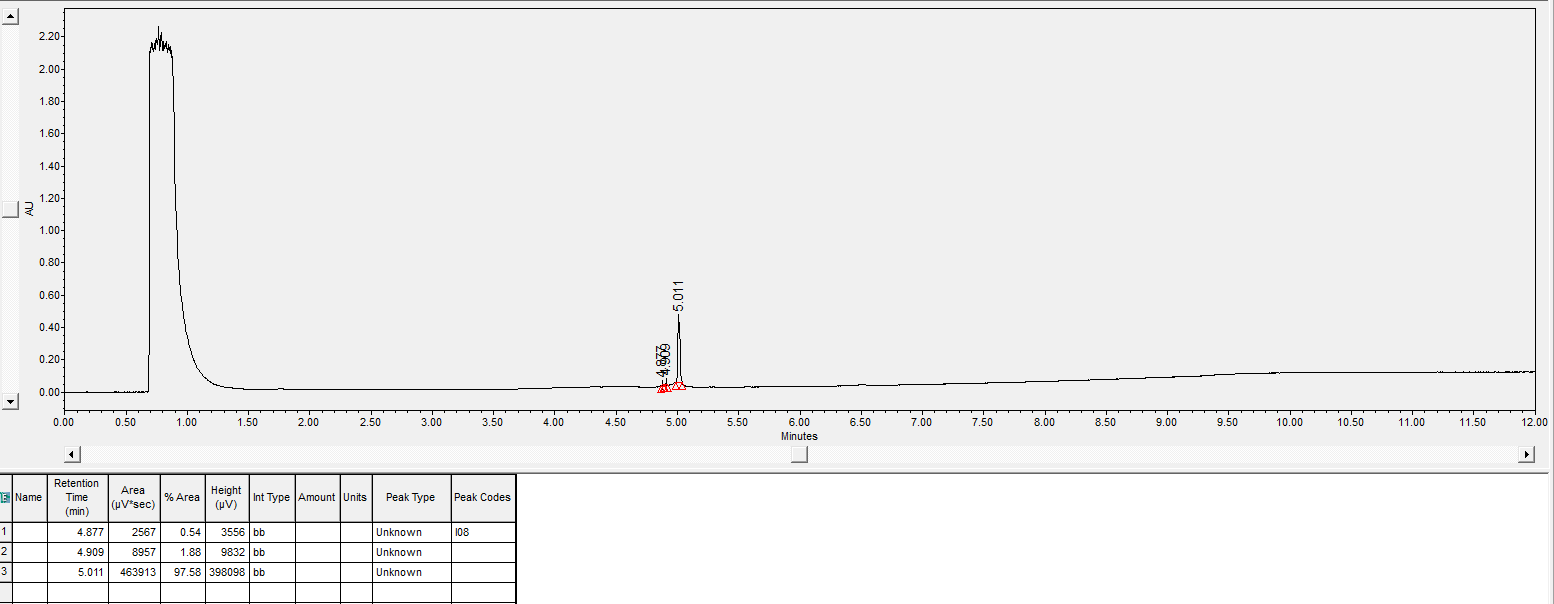


**Supplemental Figure 21.** MALDI-TOF and UPLC analysis of **HRC**. Purity = 97.6%. UPLC gradient = 10-90% MeCN/H_2_O over 10 minutes (0.3 mL/min; column = Waters Acquity BEH C4, 130Å, 1.7 µm, 2.1 x 100 mm). Detection = 220 nm.

**HRC-L:** Ac- D I S G I N A S V V N I Q K E I D R L N E V A K N L N E S L I D L Q E L GSGSGC- NH_2_

Calculated monoisotopic [M+H]^+^: 4496.3; Observed [M+H]^+^: 4496.8


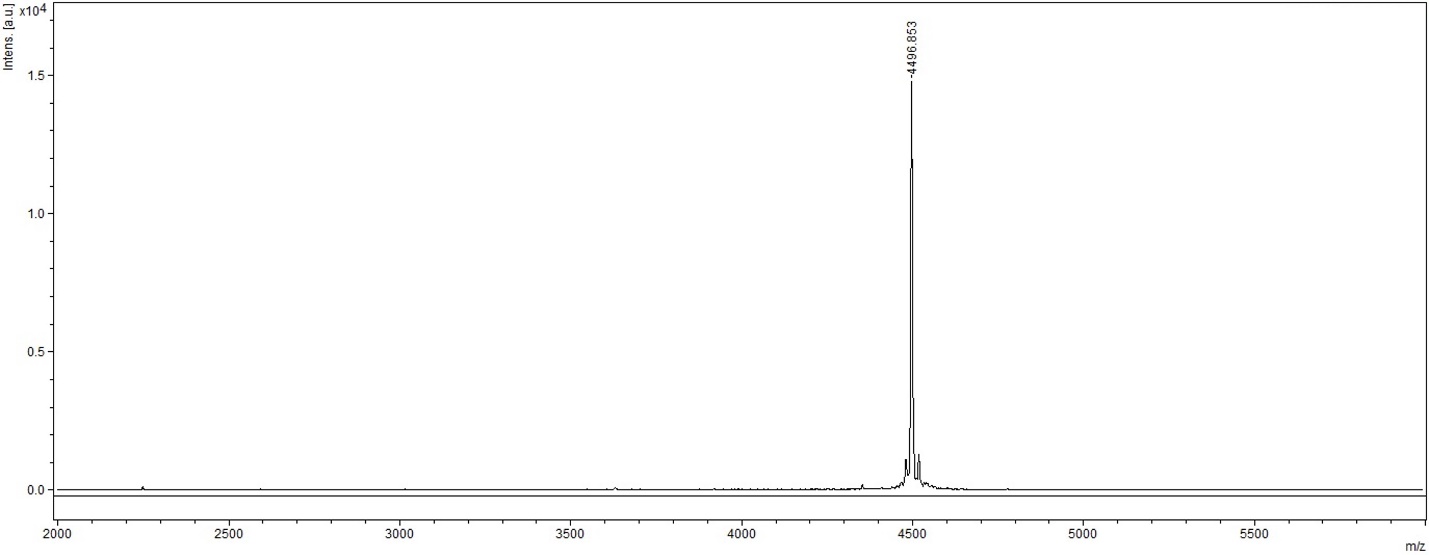


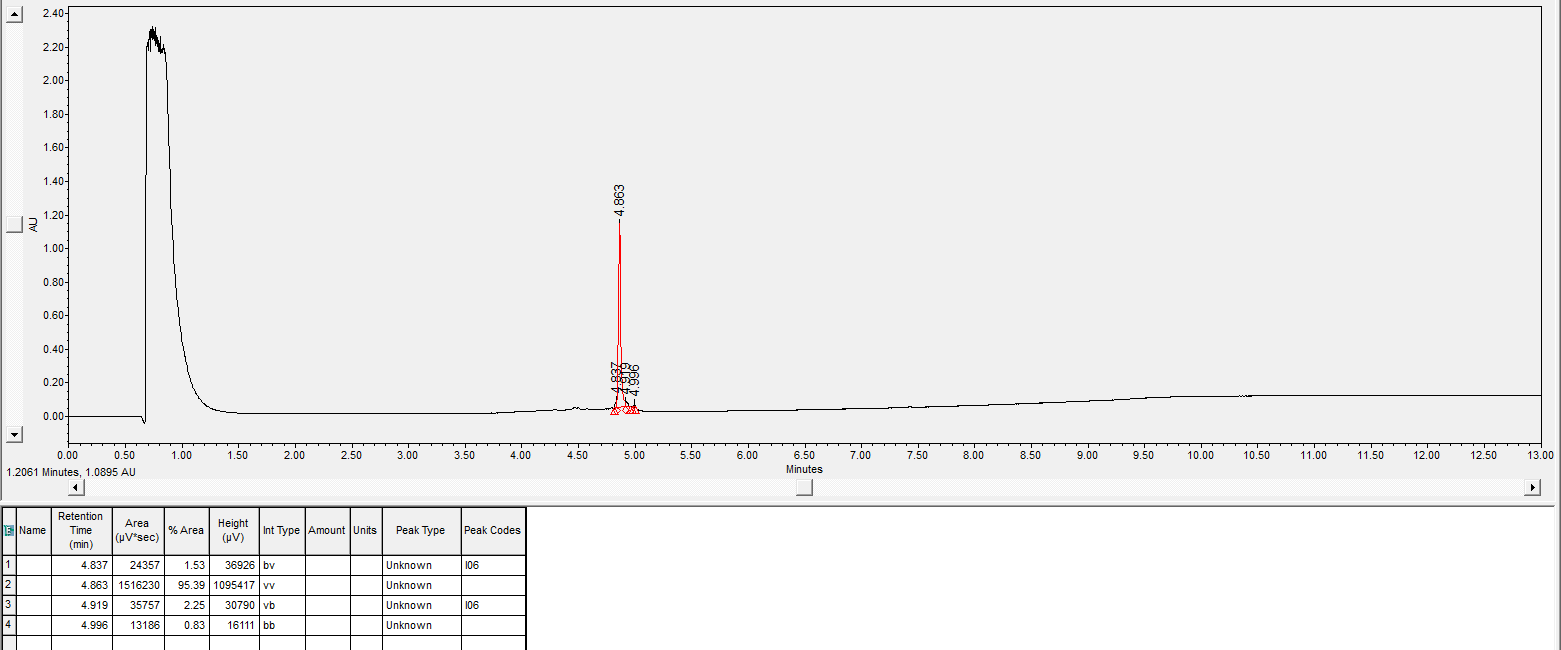


**Supplemental Figure 22.** MALDI-TOF and UPLC analysis of **HRC-L**. Purity = 95.4%. UPLC gradient = 10-90% MeCN/H_2_O over 10 minutes (0.3 mL/min; column = Waters Acquity BEH C4, 130Å, 1.7 µm, 2.1 x 100 mm). Detection = 220 nm.

**HRN-55:** Ac – T Q N V L Y E N Q K L I A N Q F N S A I G K I Q D S L S S T A S A L G K L Q D V V N Q N A Q A L N T L V K Q L - NH_2_

Calculated monoisotopic [M+H]^+^: 5983.2; Observed [M+H]^+^: 5983.0

**
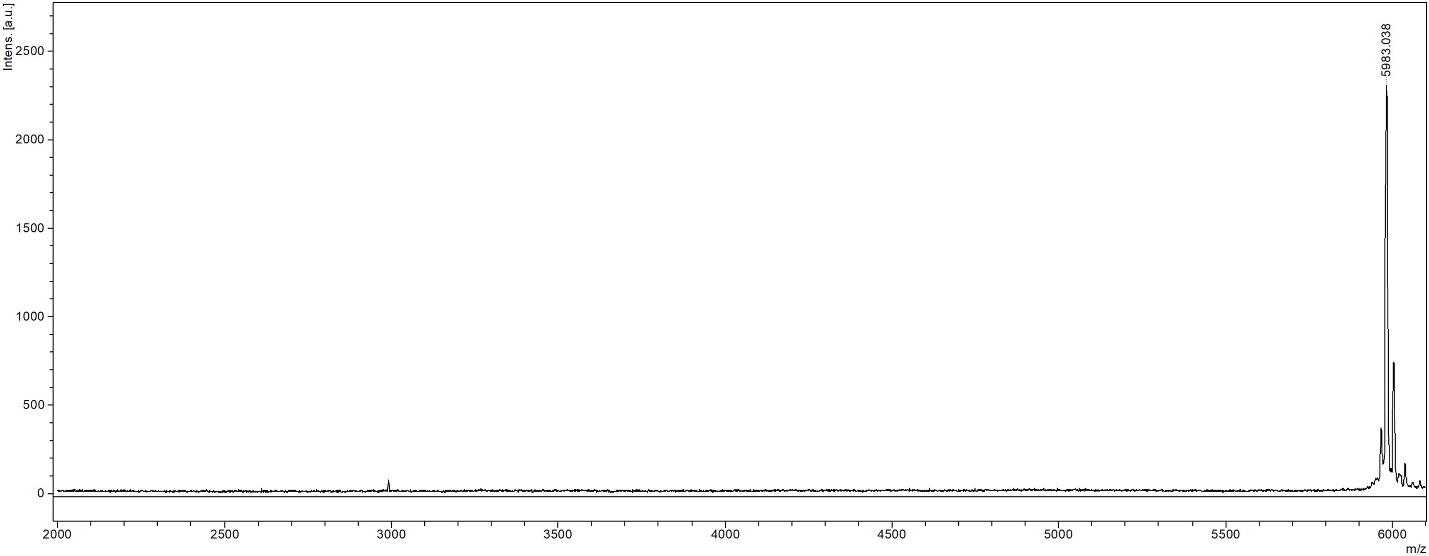
**

**
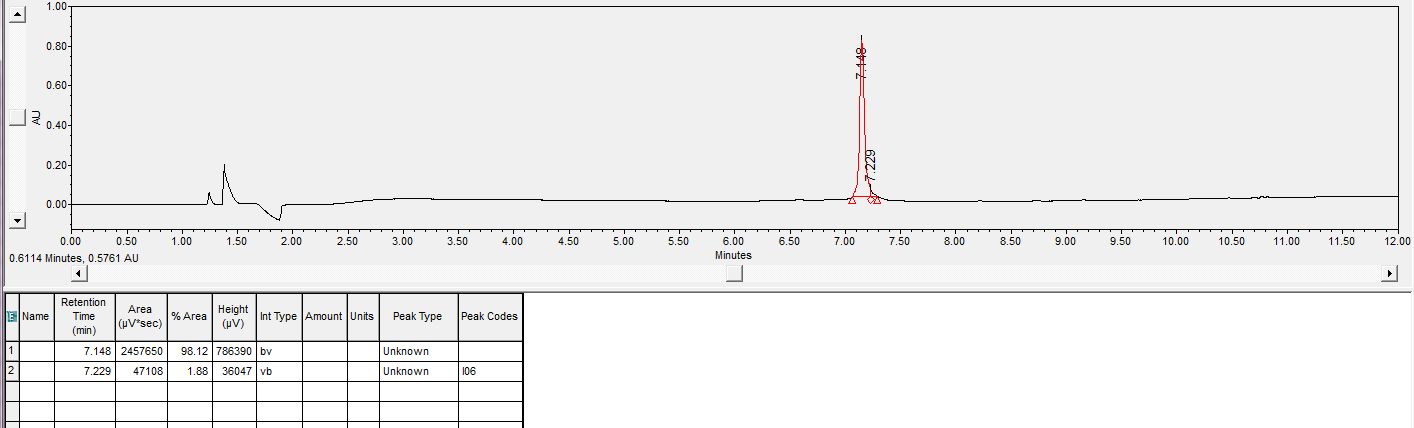
**

**Supplemental Figure 23.** MALDI-TOF and UPLC analysis of **HRN-55**. Purity = 98.1%. UPLC gradient = 10-90% MeCN/H_2_O over 10 minutes (0.3 mL/min; column = Waters Acquity CSH C18, 130Å, 1.7 µm, 2.1 x 100 mm). Detection = 220 nm.

**HRN-44:** Ac –L I A N Q F N S A I G K I Q D S L S S T A S A L G K L Q D V V N Q N A Q A L N T L V K Q - NH_2_

Calculated monoisotopic [M+H]^+^: 4652.5; Observed [M+H]^+^: 4652.6


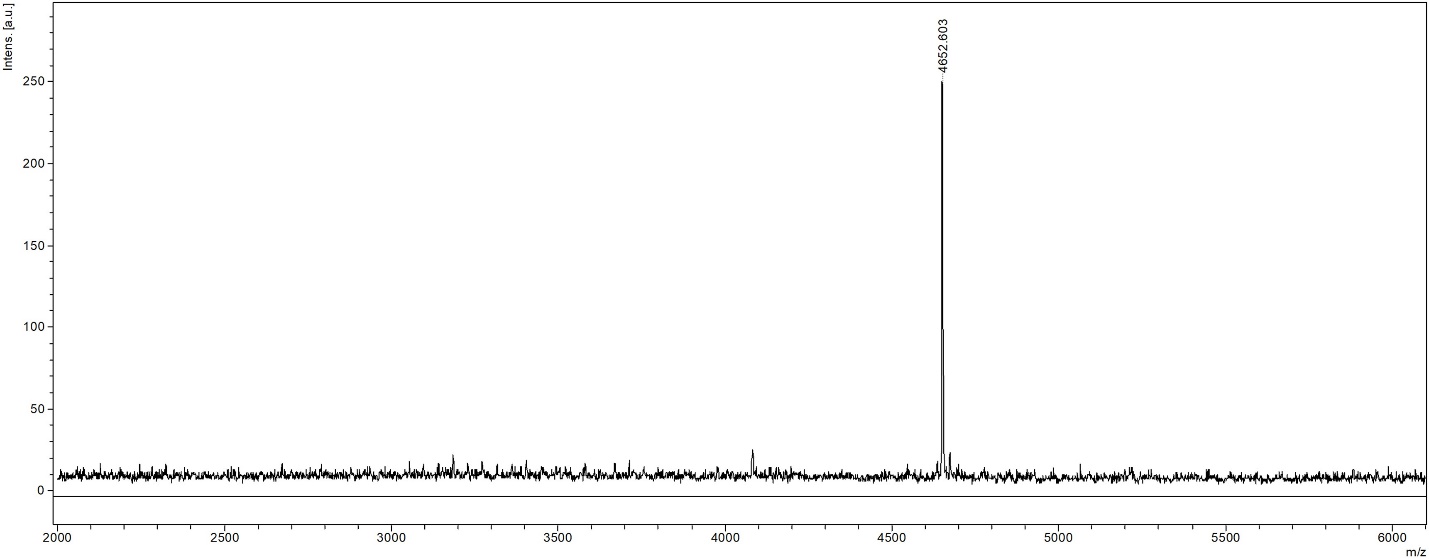


**
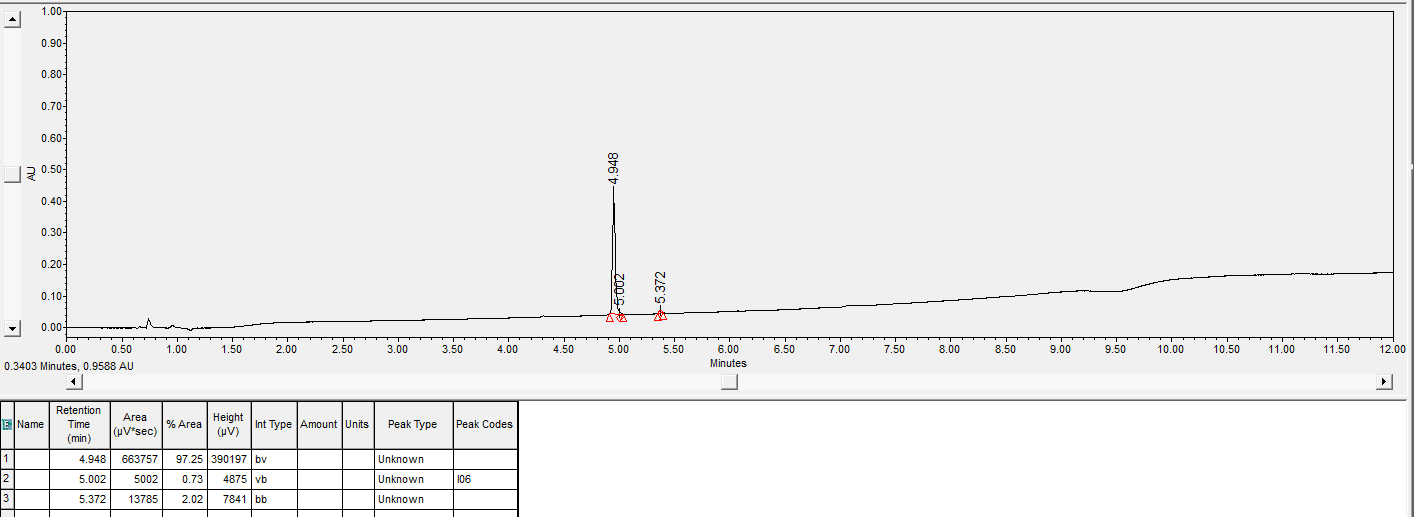
**

**Supplemental Figure 24.** MALDI-TOF and UPLC analysis of **HRN-44**. UPLC gradient = 10-90% MeCN/H_2_O over 10 minutes (0.3 mL/min; column = Waters Acquity CSH C18, 130Å, 1.7 µm, 2.1 x 100 mm). Detection = 220 nm.

**QE-A12:** Ac –D I S Q I N A S V V N A E Y E I K K L E E V A K K L E E S L I D L Q E L - NH_2_

Calculated monoisotopic [M+H]^+^: 4142.2; Observed [M+H]^+^: 4142.5


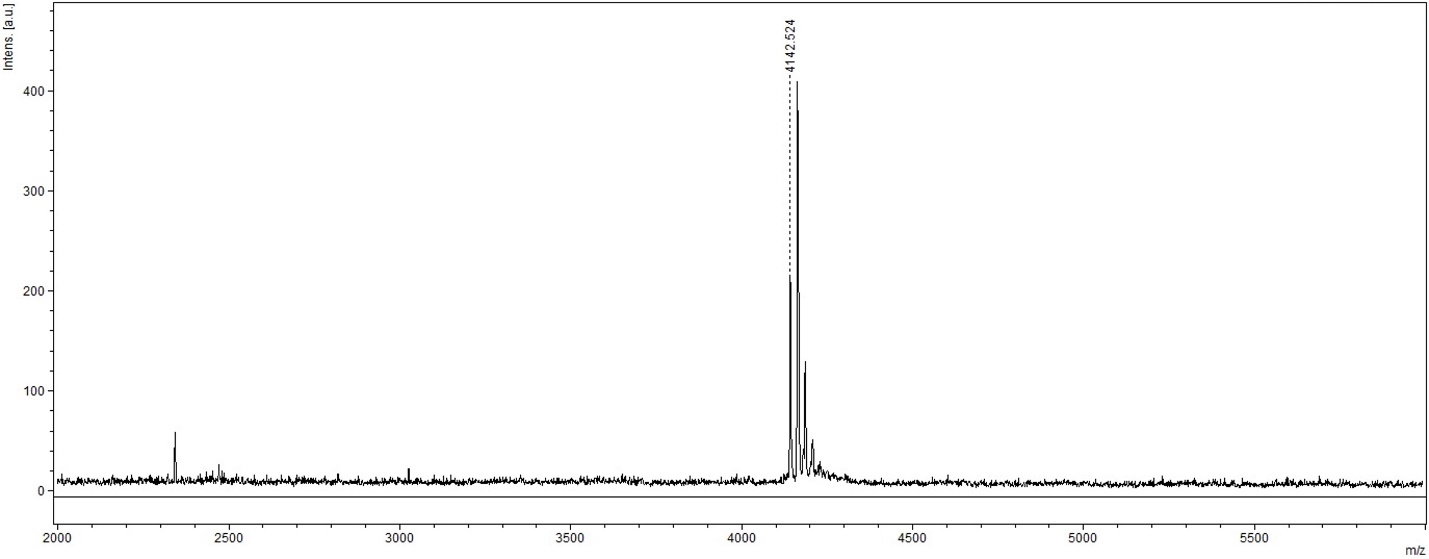


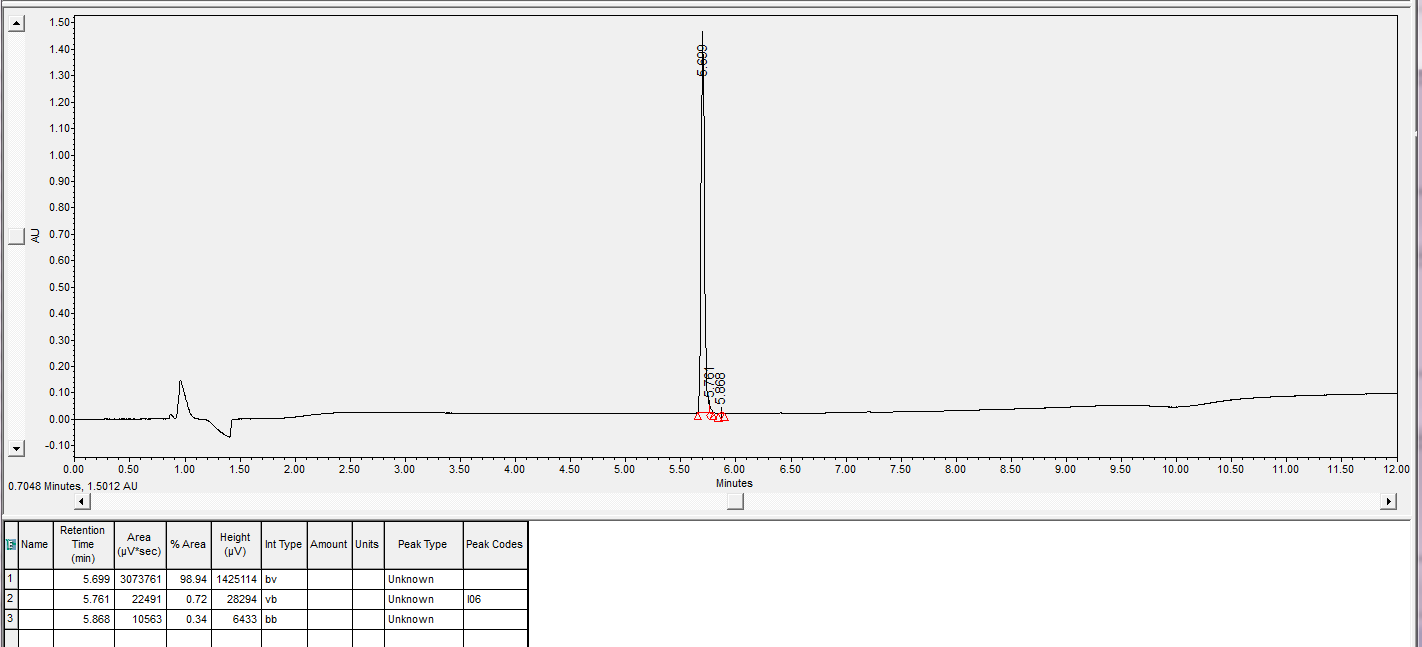


**Supplemental Figure 25.** MALDI-TOF and UPLC analysis of **QE-A12**. Purity = 98.9%. UPLC gradient = 10-90% MeCN/H_2_O over 10 minutes (0.3 mL/min; column = Waters Acquity CSH C18, 130Å, 1.7 µm, 2.1 x 100 mm). Detection = 220 nm.

**QE-25 (fragment of QE):** Ac –I E Y E I K K L E E V A K K L E E S L I D L Q E L - NH_2_

Calculated monoisotopic [M+H]^+^: 3043.7; Observed [M+H]^+^: 3043.4


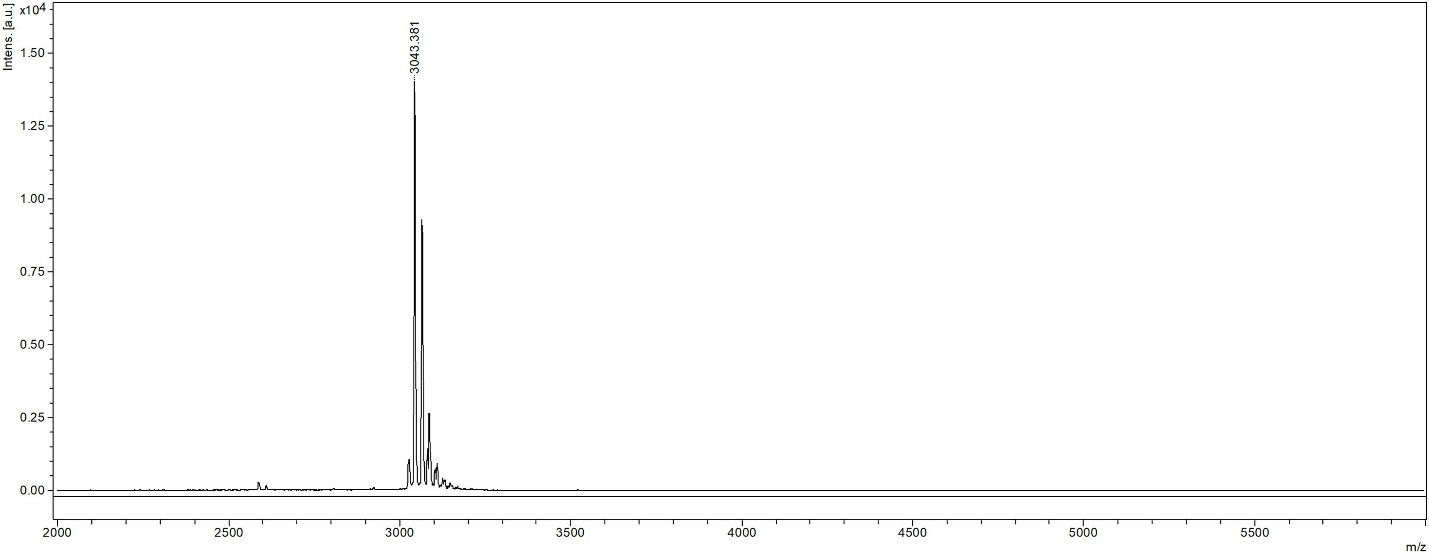


_
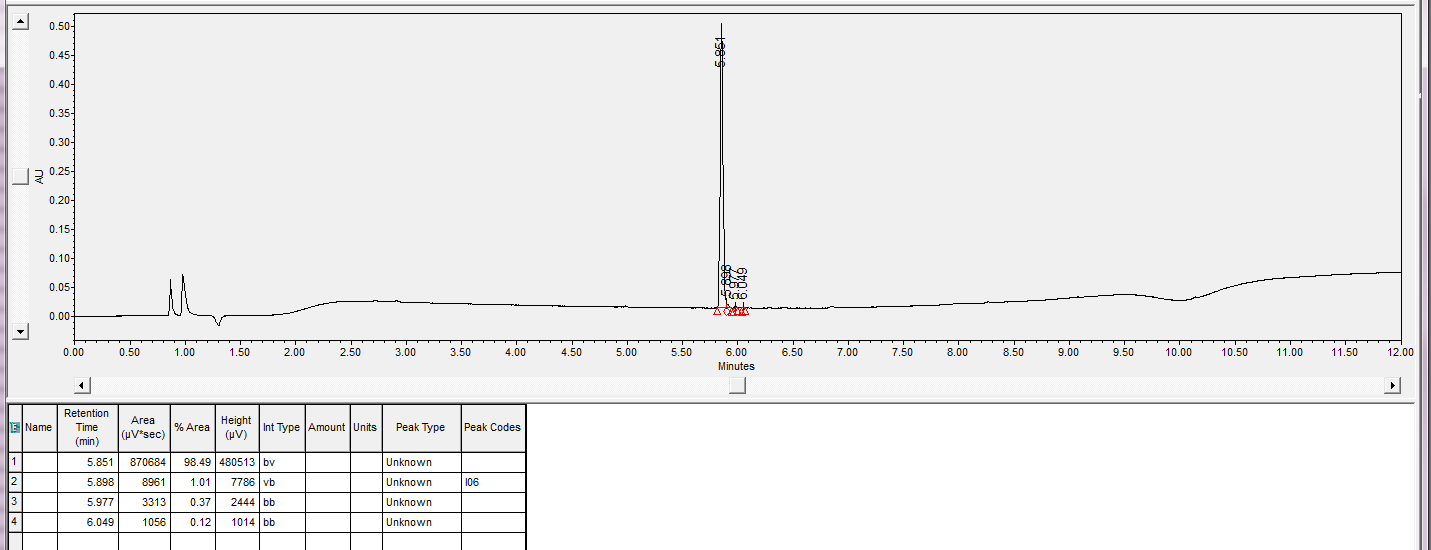
_

**Supplemental Figure 26.** MALDI-TOF and UPLC analysis of **QE-25**. Purity = 98.5%. UPLC gradient = 10-90% MeCN/H_2_O over 10 minutes (0.3 mL/min; column = Waters Acquity CSH C18, 130Å, 1.7 µm, 2.1 x 100 mm). Detection = 220 nm.

**QE-19 (fragment of QE):** Ac –I E Y E I K K L E E V A K K L E E S L - NH_2_

Calculated monoisotopic [M+H]^+^: 2332.3; Observed [M+H]^+^: 2332.0


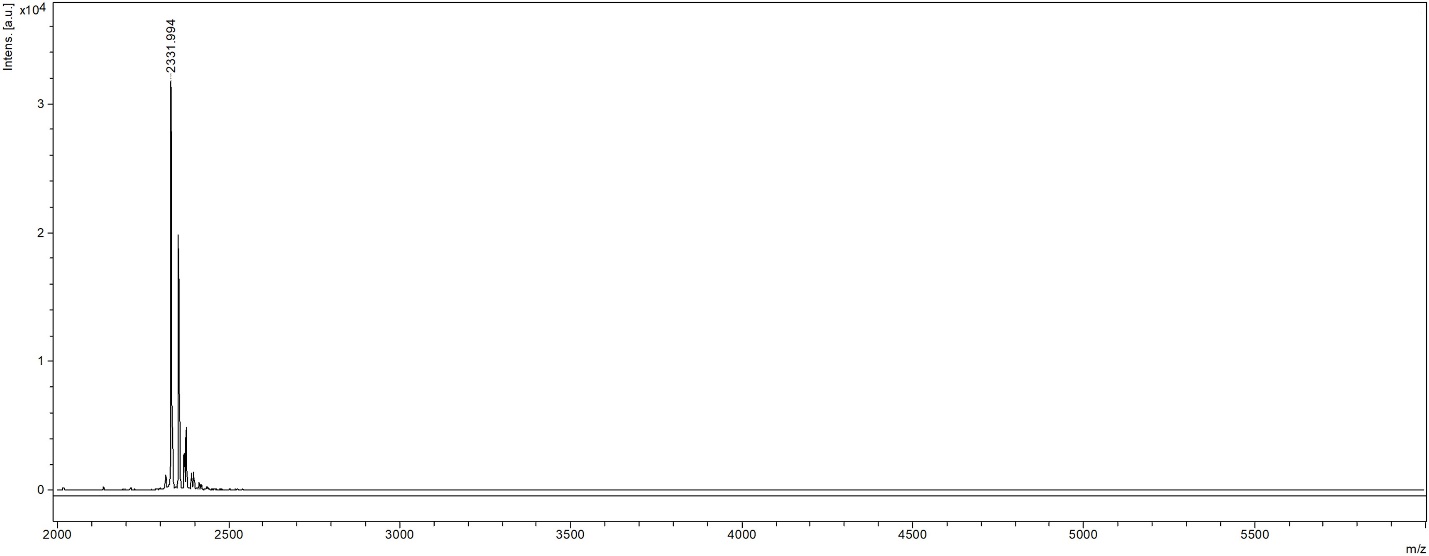


_
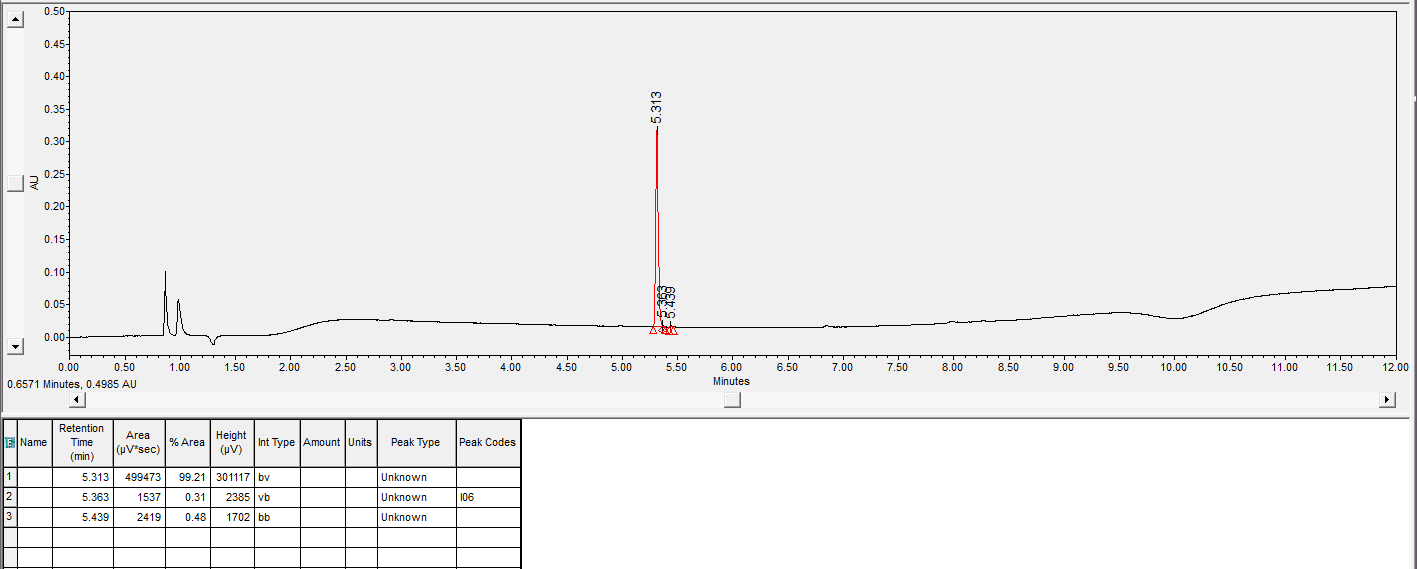
_

**Supplemental Figure 27.** MALDI-TOF and UPLC analysis of **QE-19**. Purity = 99.2%. UPLC gradient = 10-90% MeCN/H_2_O over 10 minutes (0.3 mL/min; column = Waters Acquity CSH C18, 130Å, 1.7 µm, 2.1 x 100 mm). Detection = 220 nm.

**QE-ext (N-terminally extended QE):**

Ac – PDVDLG - D I S Q I N A S V V N I E Y E I K K L E E V A K K L E E S L I D L Q E L - NH_2_

Calculated monoisotopic [M+H]^+^: 4780.5; Observed [M+H]^+^: 4780.3


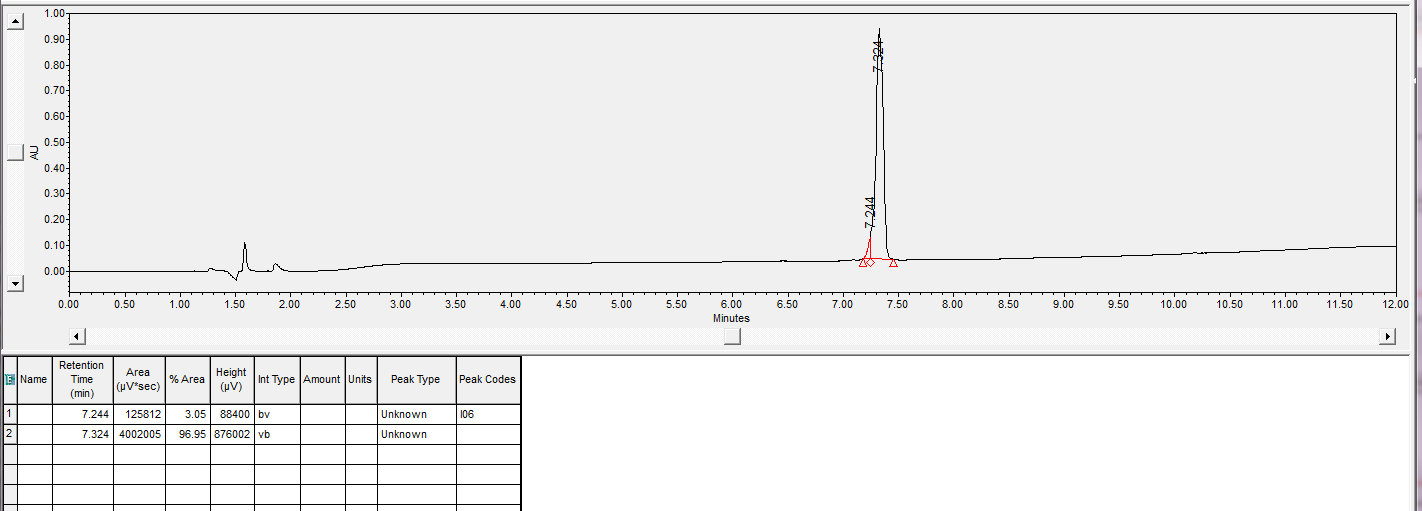

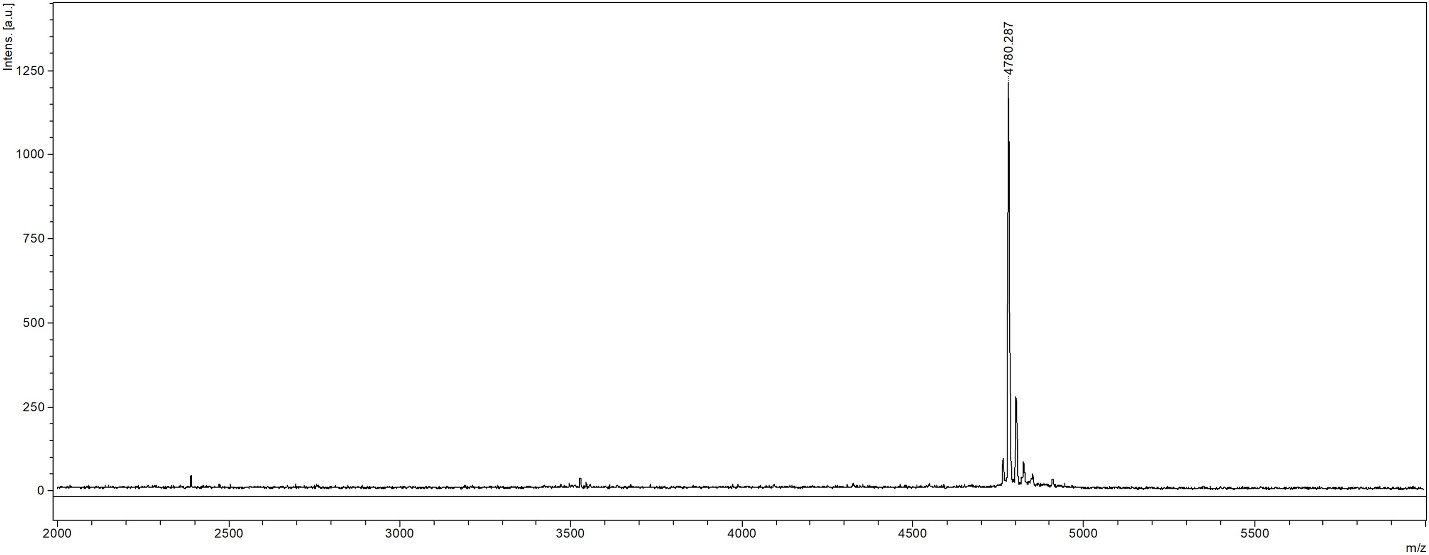


**Supplemental Figure 28.** MALDI-TOF and UPLC analysis of **QE-ext**. Purity = 96.9%. UPLC gradient = 10-90% MeCN/H_2_O over 10 minutes (0.3 mL/min; column = Waters Acquity CSH C18, 130Å, 1.7 µm, 2.1 x 100 mm). Detection = 220 nm.

**EK1:** Ac- S L D Q I N V T F L D L E Y E M K K L E E A I K K L E E S Y I D L K E L - NH_2_

Calculated monoisotopic [M+H]^+^: 4371.3; Observed [M+H]^+^: 4371.7

**
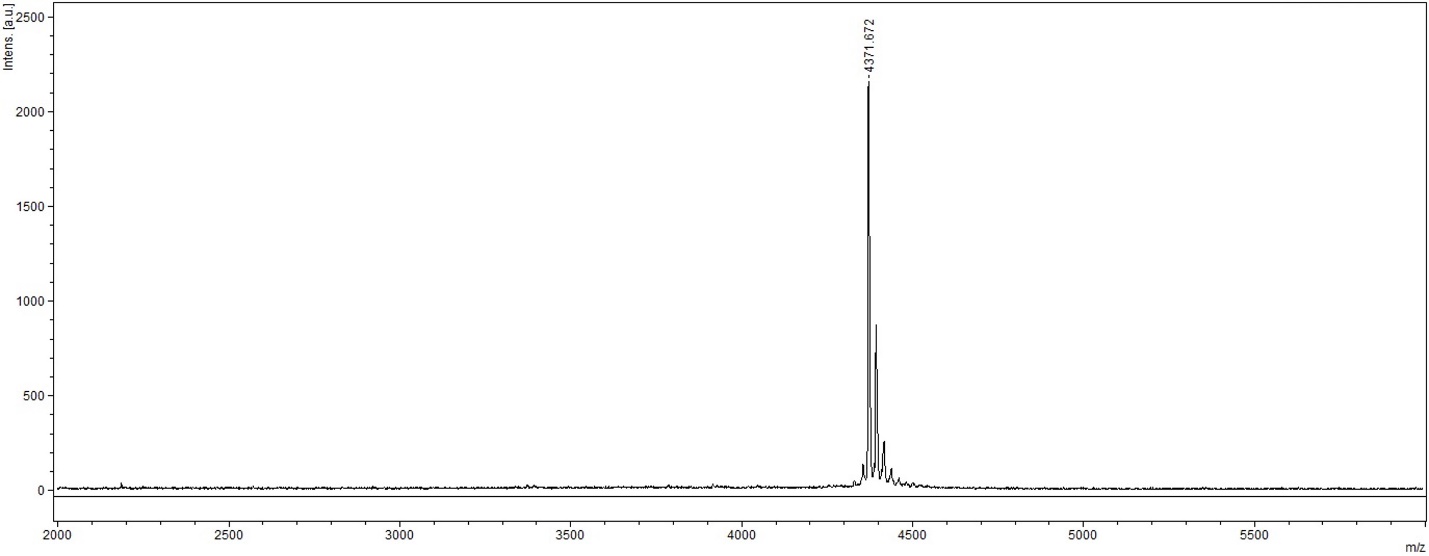
**

**
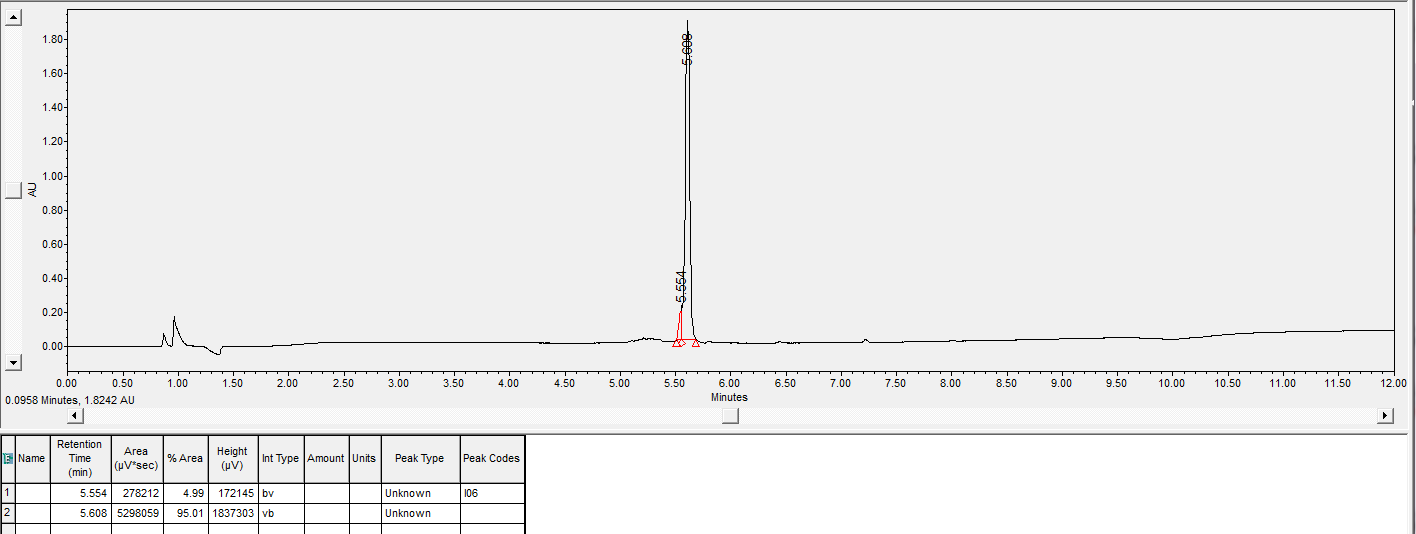
**

**Supplemental Figure 29.** MALDI-TOF and UPLC analysis of **EK1**. Purity = 95.0%. UPLC gradient = 10-90% MeCN/H_2_O over 10 minutes (0.3 mL/min; column = Waters Acquity CSH C18, 130Å, 1.7 µm, 2.1 x 100 mm). Detection = 220 nm.

**References**

1. Kabsch, W. XDS. Acta Crystallographica Section D Biological Crystallography vol. 66 125–132 (2010).
2. Vonrhein, C. et al. Data processing and analysis with theautoPROCtoolbox. Acta Crystallographica Section D Biological Crystallography vol. 67 293–302 (2011).
3. Emsley, P. & Cowtan, K. Coot: model-building tools for molecular graphics. Acta Crystallographica Section D Biological Crystallography vol. 60 2126–2132 (2004).
4. Xia, S. et al. Inhibition of SARS-CoV-2 (previously 2019-nCoV) infection by a highly potent pan-coronavirus fusion inhibitor targeting its spike protein that harbors a high capacity to mediate membrane fusion. Cell Research vol. 30 343–355 (2020).
5. Adams, P. D. et al. PHENIX: a comprehensive Python-based system for macromolecular structure solution. Acta Crystallographica Section D Biological Crystallography vol. 66 213–221 (2010).
